# Design, synthesis, in vitro, in silico, and SAR studies of flavone analogs towards anti-dengue activity

**DOI:** 10.1038/s41598-022-25836-5

**Published:** 2022-12-14

**Authors:** Apinya Patigo, Kowit Hengphasatporn, Van Cao, Wattamon Paunrat, Natthanan Vijara, Thamonwan Chokmahasarn, Phornphimon Maitarad, Thanyada Rungrotmongkol, Yasuteru Shigeta, Siwaporn Boonyasuppayakorn, Tanatorn Khotavivattana

**Affiliations:** 1 Center of Excellence in Natural Products Chemistry, Department of Chemistry, Faculty of Science, Chulalongkorn University, Bangkok, 10330 Thailand; 2 Center for Computational Sciences, University of Tsukuba, 1-1-1 Tennodai, Tsukuba, Ibaraki 305-8577 Japan; 3 Center of Excellence in Applied Medical Virology, Department of Microbiology, Faculty of Medicine, Chulalongkorn University, Bangkok, 10330 Thailand; 4 Interdisciplinary Program in Microbiology, Graduate School, Chulalongkorn University, Bangkok, 10330 Thailand; 5 Da Nang University of Medical Technology and Pharmacy, Da Nang, 50200 Vietnam; 6 Medical Sciences Program, Faculty of Medicine, Chulalongkorn University, Bangkok, 10330 Thailand; 7 Research Center of Nano Science and Technology, Department of Chemistry, College of Sciences, Shanghai University, Shanghai, 200444 People’s Republic of China; 8 Program in Bioinformatics and Computational Biology, Graduate School, Chulalongkorn University, Bangkok, 10330 Thailand; 9 Center of Excellence in Structural and Computational Biology, Department of Biochemistry, Faculty of Science, Chulalongkorn University, Bangkok, 10330 Thailand

**Keywords:** Computational chemistry, Drug discovery and development, Infectious diseases

## Abstract

Flavone has recently been proved as a promising scaffold for the development of a novel drug against dengue fever, one of the major health threats globally. However, the structure–activity relationship study of flavones on the anti-dengue activity remains mostly limited to the natural-occuring analogs. Herein, 27 flavone analogs were successfully synthesized, of which 5 analogs (**5e**, **5h**, **5o**, **5q**, and **5r**) were novel. In total, 33 analogs bearing a diverse range of substituents were evaluated for their efficacy against DENV2-infected LLC/MK2 cells. The introduction of electron-withdrawing groups on ring B such as Br (**5m**) or NO_2_ (**5n** and **5q**) enhanced the activity significantly. In particular, the tri-ester **5d** and di-ester **5e** exhibited low toxicity against normal cell, and exceptional DENV2 inhibition with the EC_50_ as low as 70 and 68 nM, respectively, which is over 300-fold more active compared to the original baicalein reference. The viral targets for these potent flavone analogs were predicted to be NS5 MTase and NS5 RdRp, as suggested by the likelihood ratios from the molecular docking study. The great binding interaction energy of 8-bromobaicalein (**5f**) confirms the anti-dengue activity at atomistic level. The physicochemical property of all the synthetic flavone analogs in this study were predicted to be within the acceptable range. Moreover, the QSAR model showed the strong correlation between the anti-dengue activity and the selected molecular descriptors. This study emphasizes the great potential of flavone as a core structure for further development as a novel anti-dengue agent in the future.

## Introduction

Dengue fever, a mosquito-borne disease caused by the dengue virus, is currently considered as a global public health concern posing threat to half of the world’s population^1^, with over 3.9 billion people at risk of infection^2^, and over 20,000 deaths annually as estimated by the World Health Organization (WHO)^3^. The dengue virus (DENV) is a single-stranded RNA virus of the family *Flaviviridae*, consisting of 4 closely related serotypes (DENV1–4). Although over 80% of the infections are generally mild, some patients may develop a severe dengue which introduce life-threatening complications such as plasma leakage and coagulopathy, leading to circulatory shock and organ impairment^4^. Since there is currently no clinically approved antiviral drug for treating the dengue infection, the treatment is only limited to supportive measures such as judicious fluid administration and close monitoring during the critical phase. This leads to serious economic consequences with the estimated global cost of dengue infection as high as US$8.9 billion per year^5^. Recently, various studies have demonstrated the correlation between the viral load and the severity of the dengue infection^6–8^. Therefore, the development of a new anti-dengue agent that can inhibit viral replication could provide a more reliable treatment for severe dengue infections, which in turn reducing the rate for hospitalization and the mortality rate of such disease^9^.

**Figure 1:**
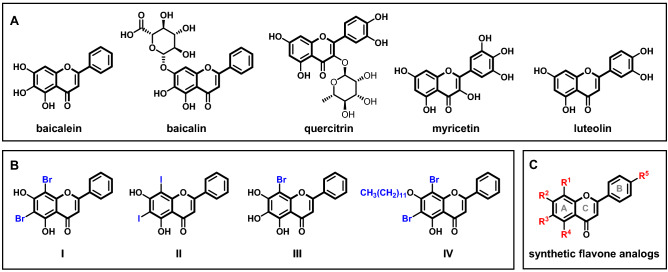
(**A**) Example of natural flavones with anti-dengue activities. (**B**) Previously reported synthetic flavone analogs and (**C**) This work.

Natural products have become an important source of new drugs to target various diseases. Over the past few decades, the screening of compounds through different approaches revealed a range of natural products that possess activity against different serotypes of the dengue virus^10^. Among them, flavone has proved to be a promising scaffold for the development of anti-dengue agents^11^. Several studies revealed that a wide range of naturally-occurring plant flavones such as baicalein^12^, baicalin^13^, quercitrin^14^, isoquercitrin^14^, myricetin^14^, kaempferol^14^, and luteolin^15^, exhibit significant anti-dengue activity, both in vitro, in vivo, and in silico (Fig. 1A). In particular, baicalein was found to inhibit the DENV2 replication in Vero cells (IC_50_ = 23.9 μM) with high selectivity index (SI = 17.8)^11,12^, and also showed interaction with NS3/NS2B, NS5 and envelop protein^16^. Moreover, the in vivo study showed that there was a tenfold reduction of the virus load in mice administered with luteolin^15^.

Despite numerous examples demonstrating the potential of flavone as a chemical scaffold for further development, the range of functional groups on the natural flavones in these studies are only limited to hydroxy, methoxy, and monosaccharides^17^. The anti-dengue activity of synthetic flavone analogs with more structural diversity remains extremely lacking. Very recently, it was shown that the introduction of halogens onto the A-ring of flavones such as in 6,8-dibromochrysin (**I**), 6,8-diiodochrysin (**II**)^18^, and 8-bromobaicalein (**III**)^19^ dramatically improves the inhibitory effect towards dengue and Zika virus, with the EC_50_ in the range of 0.66–2.20 μM (Fig. 1B). However, the introduction of a dodecyl group on 7-*O* (**IV**) as suggested by the MD pharmacophore-based virtual screening did not further improve the activity (EC_50_ = 4.17 μM)^20^. Since there is a large chemical space that remains unexplored; herein, we report a systematic structure–activity relationship study on a range of synthetic flavone analogs for their anti-dengue activity. We aim to expand the structural diversity by varying different substitution patterns on the flavone core structure, including the introduction of substituents on the A-ring via semi-synthetic routes as well as on the B-ring via total synthesis route (Fig. 1C). The knowledge from this study would be crucial for the flavone-based drug discovery towards novel anti-dengue agents.

## Results and discussion

### Synthesis of flavone analogs

**Figure 2:**
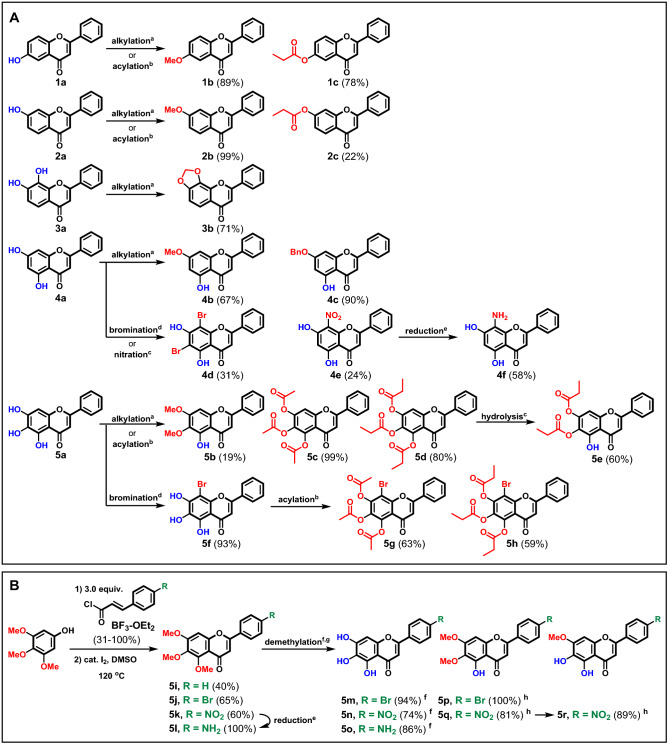
(**A**) Preparation of flavone analogs via semi-synthessis approach and (**B**) preparation of flavone analogs via total synthesis approach; ^a^alkyl halide (1.5–5.0 equiv.), K_2_CO_3_ (2.0–5.0 equiv.), acetone or DMF, 60–100 °C; ^b^acyl anhydride (30 equiv.), pyridine, rt; ^c^HNO_3_ (1.0–2.25 equiv.), AcOH, 60 °C; ^d^NBS (1.1–4.0 equiv.), THF, rt–60 °C; ^e^Sn (5.0–10.0 equiv.) 12 M HCl, EtOH; ^f^BBr_3_ (3.0 equiv), CH_2_Cl_2_, ^g^AlCl_3_ (5.0 equiv), toluene, reflux; ^h^47% HBr, AcOH, reflux.

The flavone analogs were synthesized via semi-synthetic (Fig. 2A) or total-synthetic (Fig. 2B) pathways. First, the commercially available flavones (**1a**–**5a**) were used as starting materials, which were then treated with combinations of standard functionalization conditions such as alkylation with alkyl halides^21^, acylation^22,23^, nitration^24^, reduction of nitro group^25^, and bromination^26^ to provide 17 synthetic flavone analogs (**1b**–**c**, **2b**–**c**, **3b**, **4d**–**e**, and **5b**–**h**) with a diverse range of substitution patterns on the A-ring. In particular, the alkylation of **4a** occurred selectively at the *C*-7 hydroxyl group without reacting at the *C*-5 hydroxyl group to give the products **4b** and **4c** even though the excess amount of alkylating agents were used. This is due to the intramolecular hydrogen bonding with the adjacent carbonyl leading to the decrease in the reactivity of the *C*-5 OH. This selectivity is also observed in the methylation of **5a** to give **5b**. The stability of *C*-5 OH was also observed when **5d** was treated with the aqueous acidic conditions in the attempt to perform nitration. Instead, product **5e** was obtained from the partial hydrolysis of the ester at *C*-5, which was confirmed by the presence of a singlet ^1^H NMR peak at 12.91 ppm corresponding to the intramolecular hydrogen bonded *C*-5 OH, the loss of one propionyl signal in ^1^H and ^13^C NMR, and the [M + H]^+^ signal in the HRMS.

The access to structural modifications on the B-ring requires the use of the diverted total-synthetic protocols. In this work, we selected baicalein as the core structure since 8-bromobaicalein has been proved to be the best candidate to date according to the literatures^19^. The baicalein analogs were synthesized following a previously reported 2-step protocol^27^, starting with the BF_3_-mediated Friedel–Crafts acylation of 3,4,5-trimethoxyphenol with substituted cinnamoyl chlorides to provide the corresponding chalcones, followed by iodine-mediated oxidative cyclization to provide **5i**–**l**. Notably, the oxidative cyclization to form **5j** was previously reported with 20%yield^28^; however, when the exact protocol was followed, we only observed decomposition of the corresponding chalcone. Therefore, we performed a reaction optimization and found that by reducing the amount of iodine from a stoichiometric amount (1.0 equiv.) into a catalytic amount (8 mol%), the yield of **5j** increased dramatically to 91%.

The 4′-nitro analog (**5k**) was then reduced into 4′-amino analog (**5l**) in quantitative yield. These 6,7,8-trimethoxyflavone analogs were then treated with various demethylation conditions. The use of BBr_3_ led to the total demethylation, giving **5m–o** in good yields. Partial demethylation using AlCl_3_ yielded the 6,7-dimethoxyflavone analogs (**5p**–**q**), as confirmed by the loss of one CH_3_ signal and the presence of the characteristic *C*-5 OH in the ^1^H NMR. The dimethyl analog could be further demethylated using conc. HBr to give the 7-methoxyflavone analog (**5r**). In total, 27 flavone analogs were successfully synthesized, of which 5 analogs (**5e**, **5h**, **5o**, **5q**, and **5r**) were novel. The chemical structures of all the synthesized compounds were confirmed by ^1^H and ^13^C NMR, and additionally IR and mass spectrometry analyses for novel compounds.

### Anti-dengue activity, cellular toxicity and SAR analysis

The flavone analogs were preliminary screened for their viral inhibitory activity against DENV2 NGC (accession number NC_001474.2) in a LLC/MK2 (ATCC CCL-7™) cell-based system at the final concentration of 10 μM. The result shows that the alkylation of the hydroxy groups on the A-ring typically leads to lower or no change in the inhibitory effect, as seen in analogs **1b** (77.0%), **2b** (37.5%), **3b** (NA), **4b** (16.7%), **4c** (NA), **5b** (80.0%), and **5i** (44.4%) compared to their parent non-alkylated species **1a** (90.0%), **2a** (37.5%), **3a** (58.3%), **4a** (16.7%), and **5a** (75.0%), respectively. On the other hand, the acylation of the hydroxy groups on the A-ring yielded mixed results. Notably, the triester **5c** (100%), **5d** (90.0%) and diester **5e** (91.7%) analogs of baicalein **5a** showed significant increase in the activity. The introduction of bromine substitution on the A-ring **4d** (83.0%) and **5f** (100.0%) also resulted in higher inhibitory effect, which is in agreement with the previous literatures^18,19^. However, when other substituent like NO_2_ **4e** (33.3%) or NH_2_ **4f** (NA) was introduced, little to no improvement in the activity was observed. Interestingly, the two ester analogs of 8-bromobaicalein **5f** showed stark contrast in the activity; the triacetyl analog **5g** exhibited 100% inhibition, while the tripropionyl analog **5h** was inactive towards DENV2 at 10 μM.

For the substitution on the B-ring, the introduction of either Br (**5m**) or NO_2_ (**5n**) at 4′C improved the activity significantly with 100% DENV2 inhibition. In contrast, no inhibition was observed for the NH_2_ analog (**5o**). For the 4′-bromo analog **5m** (100%), the trimethoxy **5j** (70.4%) and the dimethoxy **5p** (64.8%) counterparts showed a slight decrease in the activity. This trend is also observed in the 4′-nitro analogs **5n** (100%) vs. **5k** (70.0%) and **5q** (98.2%). On the other hand, for the 4′-amino analog **5o** (NA), the introduction of methoxy groups as in **5l** (62.0%) enhanced the inhibition activity substantially.

**Figure 3:**
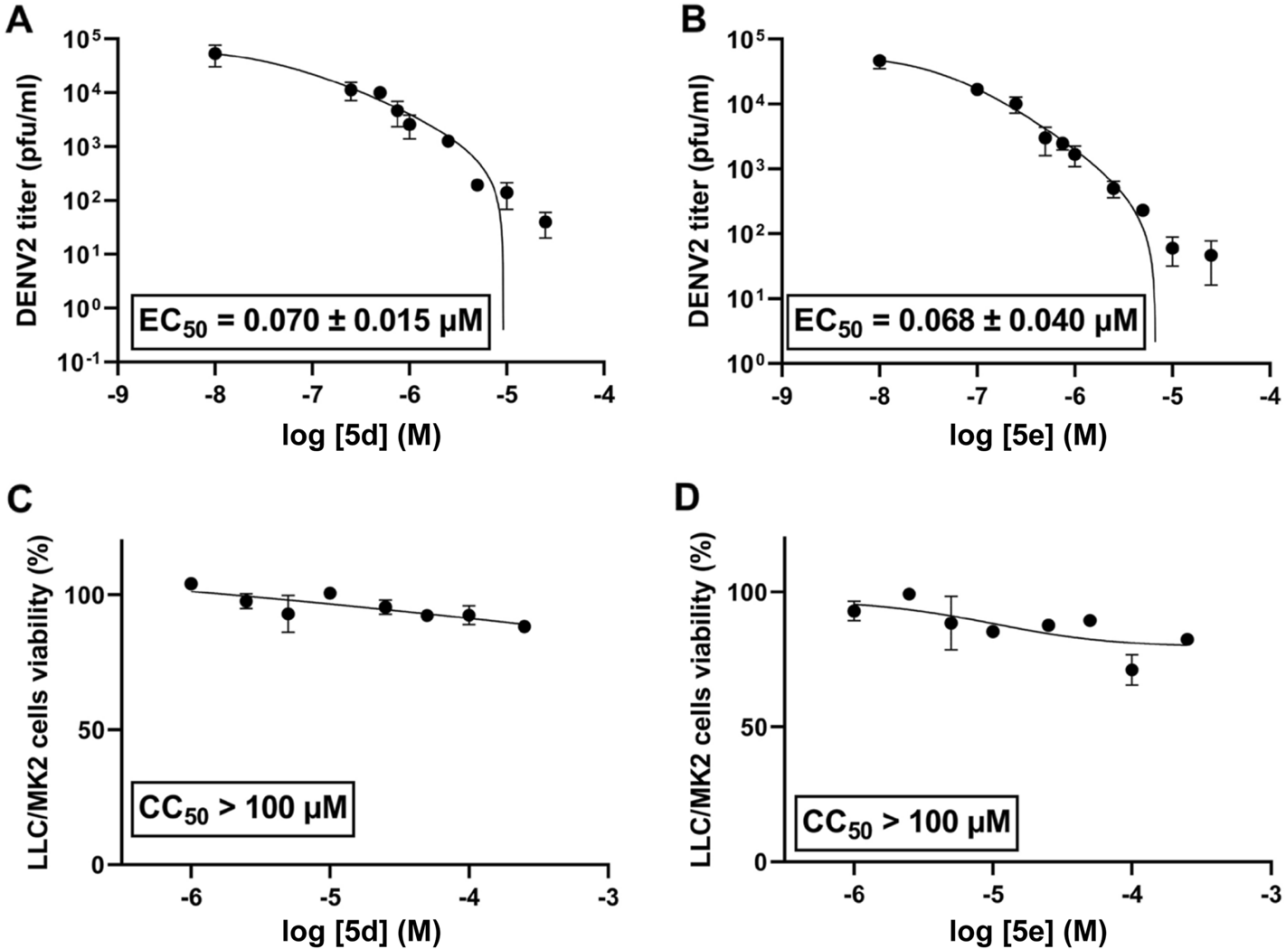
(**A**,**B**) efficacy of **5d** and **5e** against DENV2-infected LLC/MK2 cells, and (**C**,**D**) their cytotoxicities. Errors were calculated from three independent experiments.

Next, we selected 7 compounds with the viral inhibition at 10 µM above 90% (**5c**, **5d**, **5e**, **5g**, **5m**, **5n**, and **5q**) to examine their efficacy against DENV2 at various concentrations (Table 1). The EC_50_ values for most compounds were in sub-micromolar range. Strikingly, **5d** and **5e** exhibited exceptional activity with the EC_50_ of 0.070 ± 0.015 µM and 0.068 ± 0.040 µM, respectively (Fig. 3). Comparing with 8-bromobaicalein **5f** which is currently the most active flavone analog against DENV2 known in the literature at 0.88 ± 0.14 µM^19^, **5d** and **5e** exhibited more than tenfold increase in the activity, and more than 300-fold increase comparing with the original baicalein **5a**. In addition, we also found that the 4′-bromobaicalein **5m** showed slight increase in the efficacy (EC_50_ = 0.52 ± 0.12) comparing with 8-bromobaicalein^19^, while the 4′-nitro analogs (**5n** and **5q**) were relatively less active. This evidence suggests that the substituents on the B-ring also play an important role in the efficacy of these flavone analogs and more investigation can be made to improve the efficacy even further in the future.

**Table 1:** Anti-dengue activity and cytotoxicity of flavone analogs. Errors were calculated from three independent experiments.

Comp						%Inhibition^a^	EC_50_^b^ (µM)	%Viability^c^
	R^1^	R^2^	R^3^	R^4^	R^5^			
**1a**	H	H	OH	H	H	90.0	–^e^	69.50 ± 14.33
**1b**	H	H	OMe	H	H	77.0	–^e^	95.16 ± 8.12
**1c**	H	H	OPp	H	H	73.0	–^e^	89.18 ± 0.78
**2a**	H	OH	H	H	H	37.5	–^e^	121.78 ± 7.21
**2b**	H	OMe	H	H	H	37.5	–^e^	84.55 ± 3.34
**2c**	H	OPp	H	H	H	37.5	–^e^	115.24 ± 1.55
**3a**	OH	OH	H	H	H	58.3	–^e^	79.36 ± 3.20
**3b**	OCH_2_O		H	H	H	NA^d^	–^e^	96.64 ± 1.01
**4a**	H	OH	H	OH	H	16.7	–^e^	84.85 ± 2.03
**4b**	H	OMe	H	OH	H	16.7	–^e^	97.47 ± 1.00
**4c**	H	OBn	H	OH	H	NA^d^	–^e^	86.14 ± 3.05
**4d**	Br	OH	Br	OH	H	83.0	1.47 ± 0.86^f^	101.08 ± 0.44
**4e**	NO_2_	OH	H	OH	H	33.3	–^e^	52.54 ± 2.59
**4f**	NH_2_	OH	H	OH	H	NA^d^	–^e^	93.38 ± 3.29
**5a**	H	OH	OH	OH	H	75.0	23.9^f^	92.89 ± 1.44
**5b**	H	OMe	OMe	OH	H	80.0	–^e^	97.47 ± 2.47
**5c**	H	OAc	OAc	OAc	H	100	0.41 ± 0.56	107 ± 7.34
**5d**	H	OPp	OPp	OPp	H	90.0	0.070 ± 0.015	83.79 ± 2.61
**5e**	H	OPp	OPp	OH	H	91.7	0.068 ± 0.040	89.62 ± 5.95
**5f**	Br	OH	OH	OH	H	100	0.88 ± 0.14^f^	73.36 ± 14.80
**5g**	Br	OAc	OAc	OAc	H	100	0.26 ± 0.14	50.89 ± 3.22
**5h**	Br	OPp	OPp	OPp	H	NA^d^	–^e^	84.52 ± 6.56
**5i**	H	OMe	OMe	OMe	H	44.4	–^e^	96.84 ± 11.95
**5j**	H	OMe	OMe	OMe	Br	70.4	–^e^	101.13 ± 11.06
**5k**	H	OMe	OMe	OMe	NO_2_	70.0	–^e^	100.95 ± 1.97
**5l**	H	OMe	OMe	OMe	NH_2_	62.0	–^e^	93.00 ± 2.36
**5m**	H	OH	OH	OH	Br	100	0.52 ± 0.12	89.99 ± 6.61
**5n**	H	OH	OH	OH	NO_2_	100	4.30 ± 0.56	78.55 ± 9.87
**5o**	H	OH	OH	OH	NH_2_	NA^d^	–^e^	108.69 ± 2.53
**5p**	H	OMe	OMe	OH	Br	64.8	–^e^	96.78 ± 5.28
**5q**	H	OMe	OMe	OH	NO_2_	98.2	4.52 ± 0.76	84.73 ± 5.07
**5r**	H	OMe	OH	OH	NO_2_	22.2	–^e^	91.60 ± 0.89
**6a**	H	H	Me	H	H	NA^d^	–^e^	72.50 ± 2.41

^a^%viral inhibition against DENV2 at 10 μM; ^b^EC_50_: 50% effective concentration against DENV2; ^c^%viability in LLC/MK2 cell at 10 μM; ^d^NA = not active; ^e^– = not determined; ^f^data according to literature.

**Figure 4:**
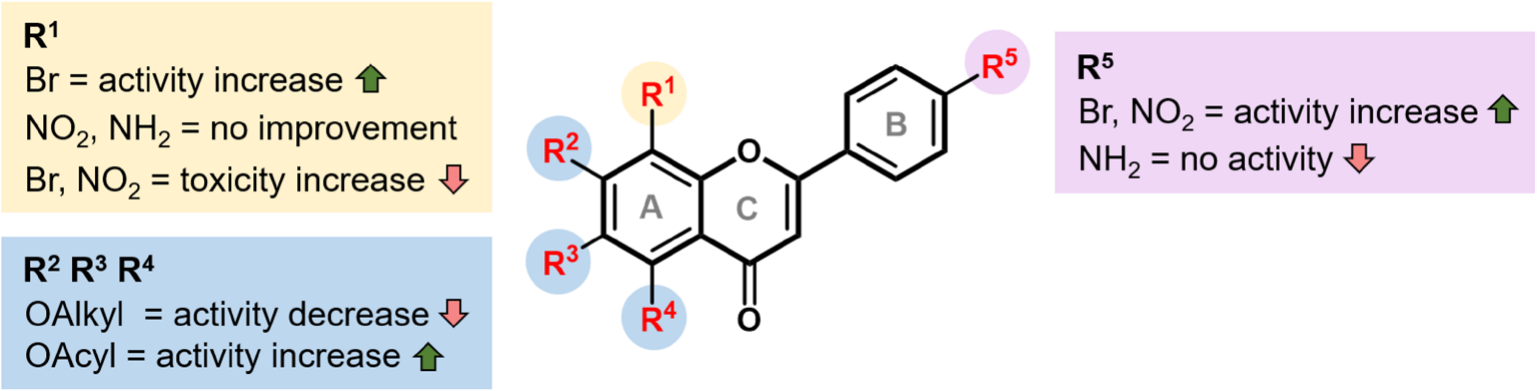
The SAR analysis.

The cytotoxicities of flavone analogs were investigated against LLC/MK2 cells at 10 µM (Table 1). Most compounds are relatively non-toxic with the viability above 75%, except for **4e** and **5g** which are moderately toxic (viability (%) = 52.54 ± 2.59% and 50.89 ± 3.22%, respectively). Therefore, these flavone analogs should be relatively safe and suitable for further consideration in the drug development process. The SAR analysis is summarized in Fig. 4.

### Molecular docking studies

**Figure 5:**
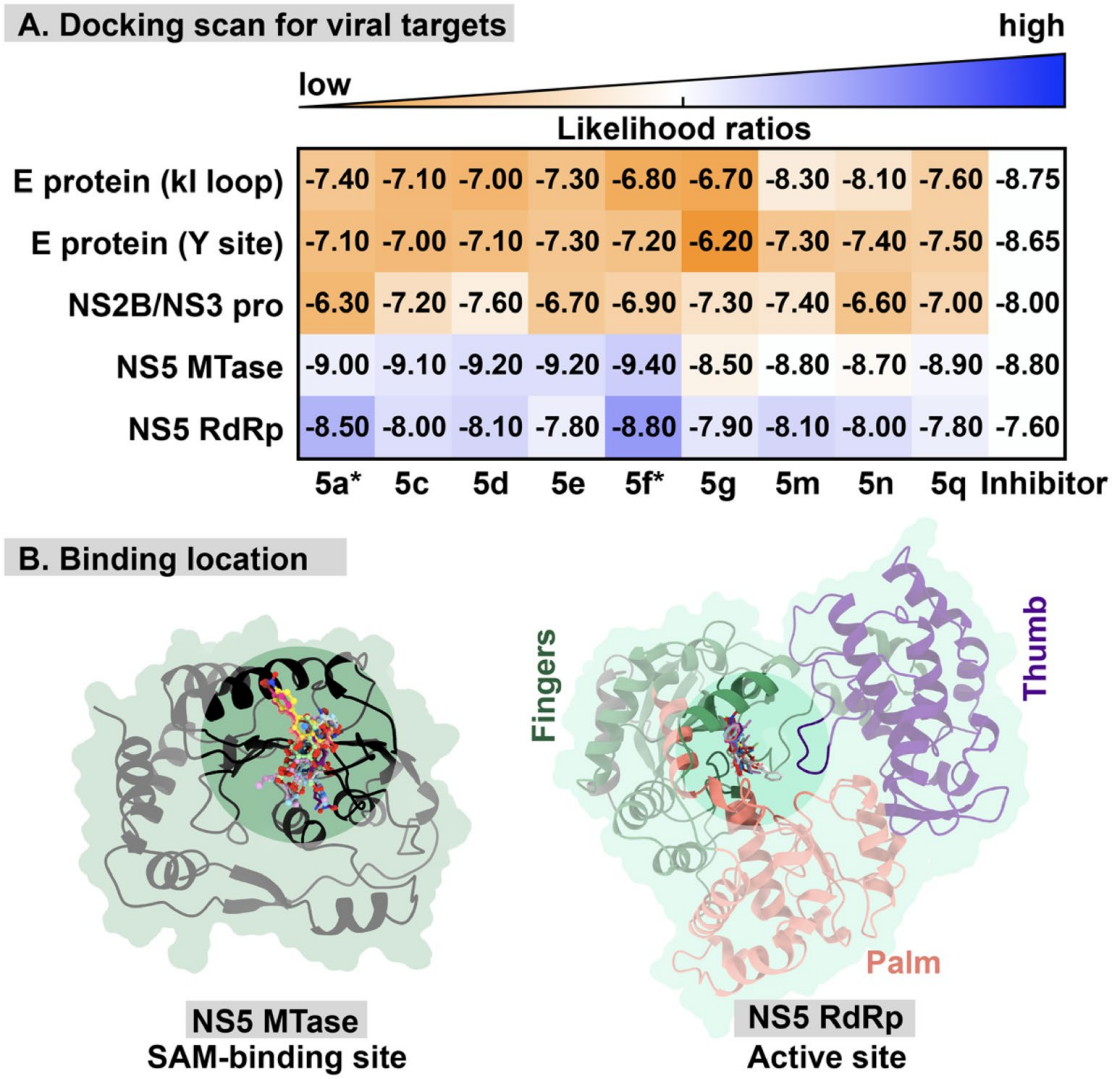
(**A**) Molecular docking scanning result of the potent flavone analogs against that viral promising target protein was plotted versus the native inhibitor of each protein. The color in the grid plot corresponds to the likelihood of the compound targeting a viral protein that ranges from low to high (orange-white-blue) using the known inhibitor as reference data (white). (**B**) The binding location for flavones on NS5 MTase and NS5 RdRp. Note that the molecular result of **5a** and **5f** were derived from our previous study^19^.

The inhibition mechanism of flavones to DENV infected cell has been reported as a multitarget mode of action. This study focused on the viral protein as a promising target for these flavone analogs. From a molecular perspective, the docking study was employed to predict the possible viral protein target for the potent flavone analogs (**5c**, **5d**, **5e**, **5g**, **5m**, **5n**, and **5q**) suggested by the anti-dengue activity and cytotoxicity assays. In addition, **5a**, **5f**, and native inhibitors of each viral protein target were also included in this step as a reference binding free energy. In this study, the 3D conformers of interested flavone analogs were constructed and docked with the viral target protein to analyze the binding interaction energy. The possible target was identified by comparing their binding interaction score to the native inhibitor called the likelihood ratios (Fig. 5 and Table S1 in the Supporting Information), which is described in the Methodology section below. A molecular docking study and likelihood ratios implied that the NS5 MTase and NS5 RdRp were likely to be the preferential viral targets for these potent flavone analogs, particularly **5f** compound. However, the binding pattern and interaction of these compounds were further elucidated for each viral target.

**Figure 6:**
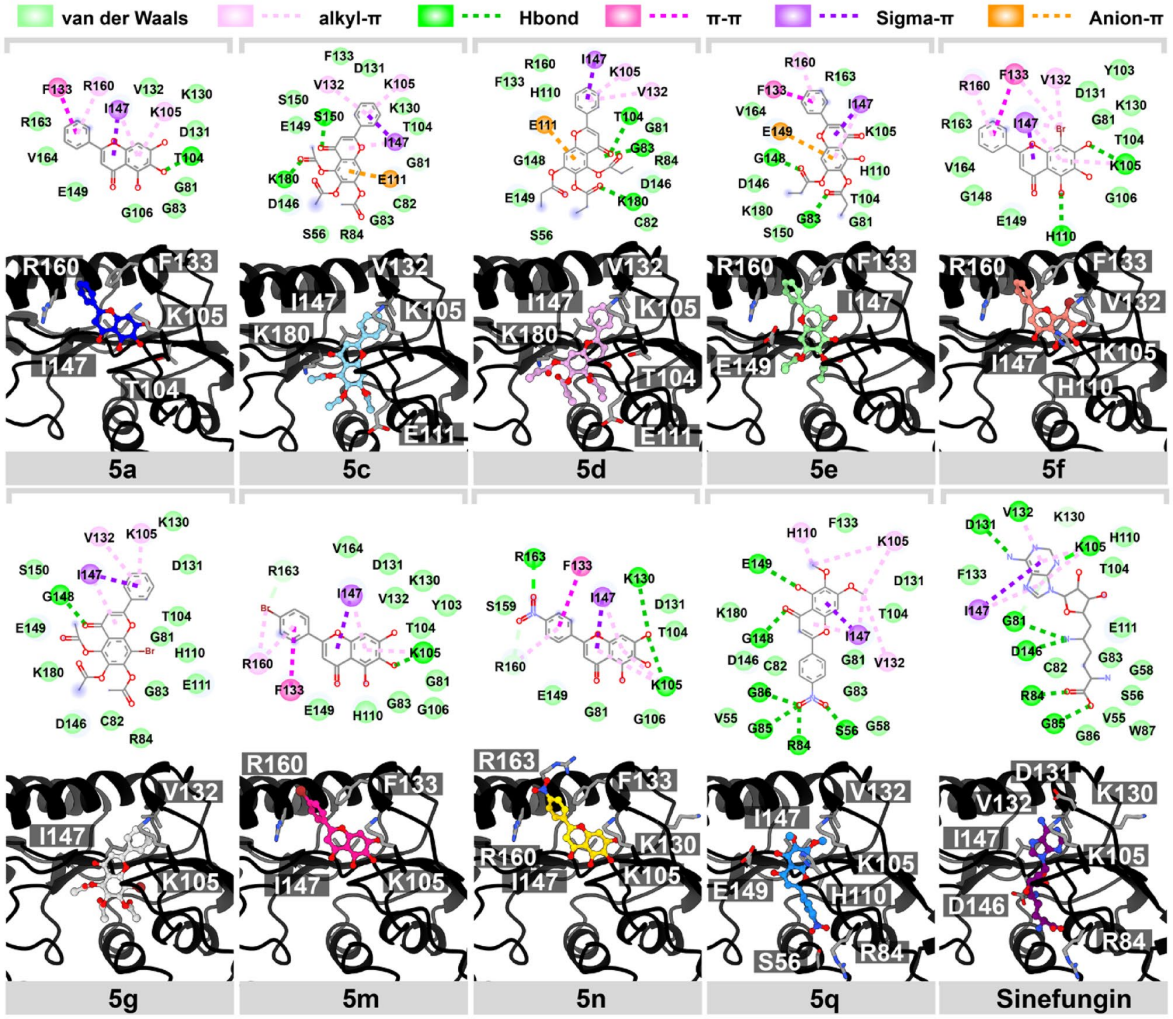
2D and 3D interaction view of potent flavone analogs and NS5 MTase's native inhibitor interacting in the SAM binding pocket of NS5 MTase.

For NS5 MTase, we found that the ester modification (**5c**–**d**) or the bromine substitution on A-ring (**5f**)^19^ of the original baicalein significantly enhances the intermolecular interaction (− 9.10 to − 9.40 kcal/mol) to the SAM binding site of NS5 MTase, while the others reveal slightly worse binding interaction score compared to NS5 MTase inhibitor, sinefugin. Interestingly, the presence of both ester moieties and bromine substituent on the A-ring led to a dramatic decrease in the binding interaction (**5g**, − 8.50 kcal/mol), as we surmise that the excessive steric bulk on the A-ring could hinder the favorable interaction within the pocket. By observing the binding conformation, **5c**, **5d**, **5e**, and **5g** insert ring B into the pocket and align rings A and C to the groove, which is close to the conformation of sinefungin. This ligand/binding pattern is also similar to those of the previously reported dibromopinocembrin and dibromopinostrobin with potent antiviral activity^29^. For the ring B modification (**5m** and **5n**), these flavone analogs showed a similar binding pattern to those of the parent compound (**5a**) and 8-bromobaicalein (**5f**), aligning ring A and C to the purine group of sinefungin. Although these compounds' alignment is not covered in the SAM binding site, they exhibited moderate to excellent binding affinity due to the hydrophobic interaction with several key residues in this site (Fig. 6). Besides the hydrophobic force from the flavone core structure, it is clear that the displaced bromine in the scaffold also enhances the hydrophobic and halogen interaction between the ligand and the surrounding amino acids, which leads to the increase of ligand binding strength, as shown in **5f**. The brominated compound could be a superior potent inhibitor than the parent structure.

**Figure 7:**
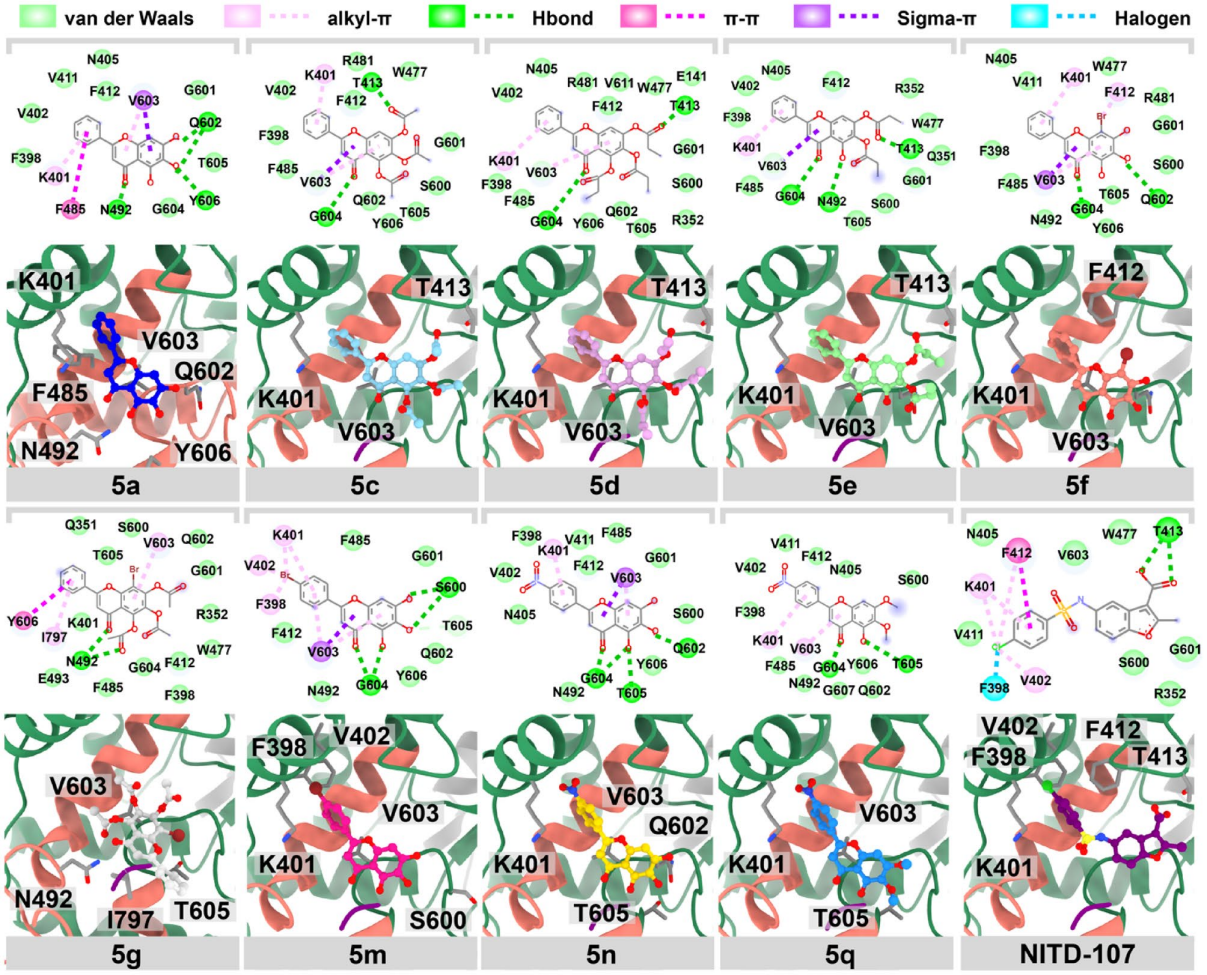
2D interaction diagram and 3D view of potent flavone analogs and NS5 RdRp's native inhibitor interacting in the binding pocket of NS5 RdRp.

For NS5 RdRp (Fig. 7), most flavone analogs showed similar binding conformers at the finger domain, which is the exact location for the NS5 RdRp's native inhibitor, NITD-107. Although **5g** showed a good anti-dengue activity, the molecular mechanism revealed that this compound did not fit well in this domain. This possibly due to the presence of four relatively large substituents on the A-ring. The hydrophobic interaction of 8-Brominated baicalein (**5f**) played a major contribution towards the binding of these flavones within the active site of NS5 RdRp, resulting in this likely to be the most potent inhibitor, with the binding strength of − 8.90 kcal/mol, surpassing that of NITD-107 (− 7.60 kcal/mol)^19^. Moreover, bromine substitution on ring A (**5f**) or B (**5m**) could improve the binding strength through halogen interaction, which is in line with the antiviral activity against SAR-CoV2, dengue, and zika viruses, in the previous studies^20,29–31^. The NO_2_ substitution (**5n** and **5q**) at ring B did not significantly improve the binding strength^19^. Moreover, we found that the ester modification on ring A (**5c**, **5d**, **5e**, and **5g**) led to the decrease in the binding interaction, despite their exceptional anti-dengue activities^19^. These ester analogs may alternatively serve as prodrugs, improving the the solubility or the delivery system, which are then hydrolyzed within cells before inhibiting the viral target in the active form^32^.

### Physicochemical properties prediction

**Figure 8:**
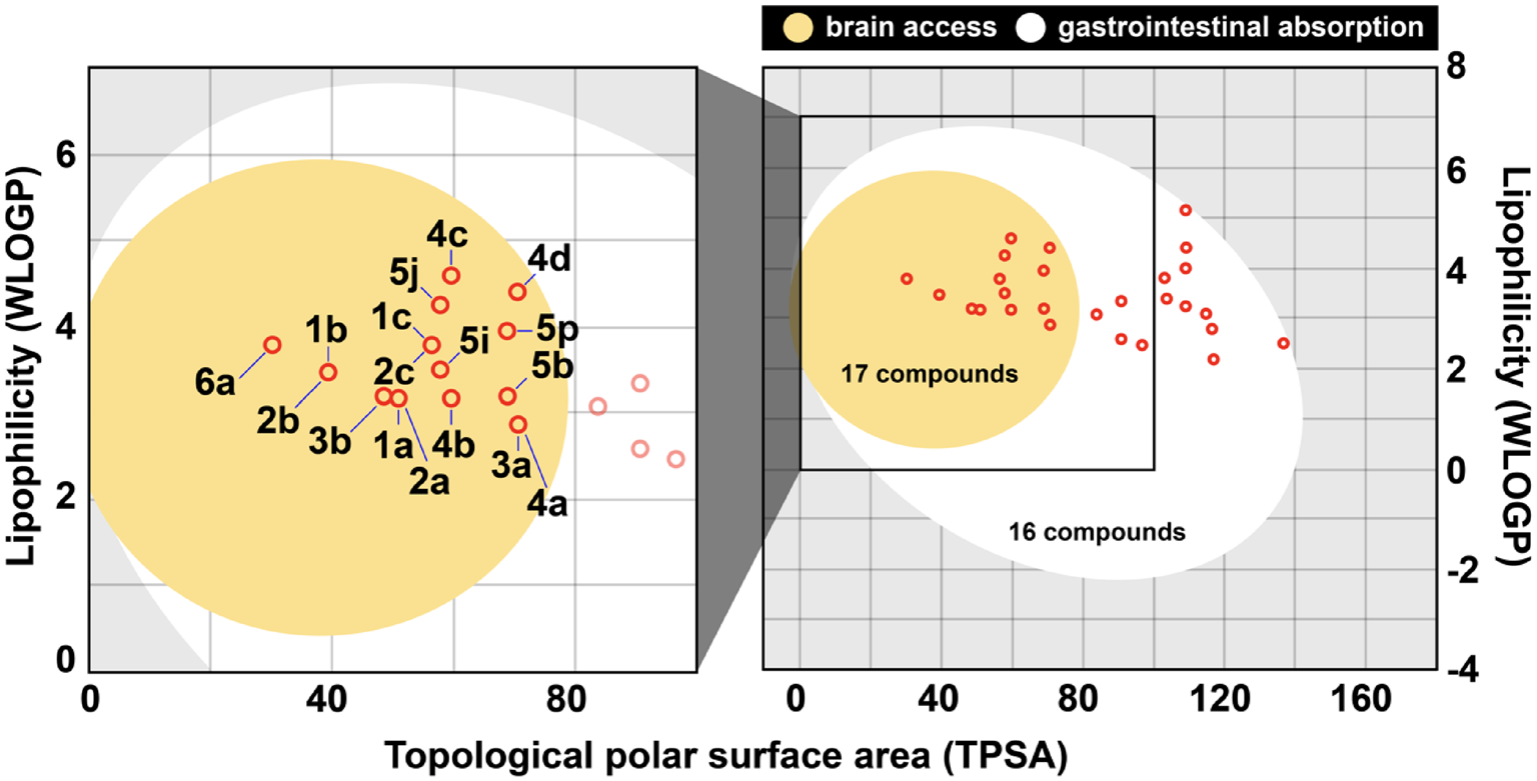
The passive gastrointestinal absorption (egg white area) and brain access across the blood–brain barrier (yolk area) prediction for 33 flavone analogs were plotted as a BOILED-Egg model.

The physicochemical properties and drug-likeness of the 33 flavone analogs were predicted by SwissADME^33^ and ADMETlab 2.0^34^ based on Lipinski's rule of five^35^ and the Golden Triangle^36^, which are the famous criterion for pharmaceutical industries worldwide. Most of these molecules were acceptable within the criterion, except for **5h** due to its slightly higher MW (Table 2). Moreover, SWISSADME was applied to predict gastrointestinal (GI) absorption and blood–brain barrier (BBB) permeabilities of these analogs. Considering the lipophilicity prediction (log P) and total polar surface area (TPSA) calculation, which are the key properties related to drug bioavailability, all flavone analogs possess the values of log P in a range of − 2 to 7 and TPSA lower than 140. This indicates that the molecules can be passively absorbed through the gastrointestinal tract, as shown in the egg-white region in Fig. 8, hence classified as non-substrate of the permeability glycoprotein (PGP−), which is one of the explanation for the higher permeability of molecule. Notably, only 17 molecules located in a yolk area could permeate the blood–brain barrier, making these promising lead compounds excavate and discover novel anti-dengue inhibitors. The existence of the hydrophilic substituents such as NH_2_ or NO_2_ hinders the permeation through the blood–brain barrier as observed in some flavone analogs.

**Table 2:** Predicted physicochemical properties and drug-likeness of flavone analogs.

Comp	Drug-likeness parameters					Verber parameters			Others	
	MW^a^	HBD^b^	HBA^c^	Log P^d^	Lip. Vio^e^	TPSA^f^	No. of RB^g^	Ver. Vio^h^	Log S^i^	MR^j^
**1a**	238.24	1	3	3.62	0	50.44	1	0	− 4.19	69.94
**1b**	252.26	0	3	3.95	0	39.44	2	0	− 4.38	74.41
**1c**	294.30	0	4	4.20	0	56.51	4	0	− 4.58	84.23
**2a**	238.24	1	3	3.62	0	50.44	1	0	− 4.19	69.94
**2b**	252.26	0	3	3.95	0	39.44	2	0	− 4.38	74.41
**2c**	294.30	0	4	4.20	0	56.51	4	0	− 4.58	84.23
**3a**	254.24	2	4	3.26	0	70.67	1	0	− 4.03	71.97
**3b**	266.25	0	4	3.37	0	48.67	1	0	− 4.14	73.98
**4a**	254.24	2	4	3.52	0	70.67	1	0	− 4.19	71.97
**4b**	268.26	1	4	3.85	0	59.67	2	0	− 4.39	76.44
**4c**	344.36	1	4	5.34	0	59.67	4	0	− 5.70	100.92
**4d**	412.03	2	4	4.40	0	70.67	1	0	− 5.66	87.37
**4e**	299.24	2	6	3.90	0	116.49	2	0	− 4.56	80.79
**4f**	269.25	3	4	2.84	0	96.69	1	0	− 3.82	76.37
**5a**	270.24	3	5	3.16	0	90.90	1	0	− 4.03	73.99
**5b**	298.29	1	5	3.82	0	68.90	3	0	− 4.44	82.93
**5c**	396.35	0	8	2.44	0	109.11	7	0	− 3.78	102.42
**5d**	438.43	0	8	3.85	0	109.11	10	0	− 4.69	116.84
**5e**	382.36	1	7	3.82	0	103.04	7	0	− 4.58	102.56
**5f**	349.13	3	5	3.36	0	90.90	1	0	− 4.62	81.69
**5g**	475.24	0	8	3.13	0	109.11	7	0	− 4.69	110.12
**5h**	517.32	0	8	4.54	1	109.11	10	0	− 5.61	124.54
**5i**	312.32	0	5	3.09	0	57.90	4	0	− 3.97	87.40
**5j**	391.21	0	5	3.79	0	57.90	4	0	− 4.88	95.10
**5k**	357.31	0	7	2.92	0	103.72	5	0	− 4.02	96.22
**5l**	327.33	1	5	2.41	0	83.92	4	0	− 3.62	91.80
**5m**	349.13	3	5	3.71	0	90.90	1	0	− 4.84	81.69
**5n**	315.23	3	7	2.85	0	136.72	2	0	− 3.97	82.81
**5o**	285.25	4	5	2.34	0	116.92	1	0	− 3.58	78.39
**5p**	377.19	1	5	4.36	0	68.90	3	0	− 5.24	90.63
**5q**	343.29	1	7	3.50	0	114.72	4	0	− 4.38	91.75
**5r**	329.26	2	7	3.18	0	125.72	3	0	− 4.18	87.28
**6a**	236.27	0	2	3.92	0	30.21	1	0	− 4.37	72.89

^a^MW = Molecular weight: ≤ 500; ^b^HBD = Hydrogen bond donors: ≤ 5; ^c^HBA = Hydrogen bond acceptors: ≤ 10; ^d^Log P = log of octanol to water partition coefficient: ≤ 5; ^e^Lip. Vio. = Lipinski Violations; ^f^TPSA = Total polar surface area [A°]^2^: ≤ 140; ^g^No. of RB = Number of rotatable bonds: ≤ 10; ^h^Ver. Vio = Verber Violations; ^i^Log S = log of aqueous solubility (mol/L): − 6 to 0; ^j^MR = Molecular refractivity [cm^3^/mol]: 40–130.

### QSAR model

**Figure 9:**
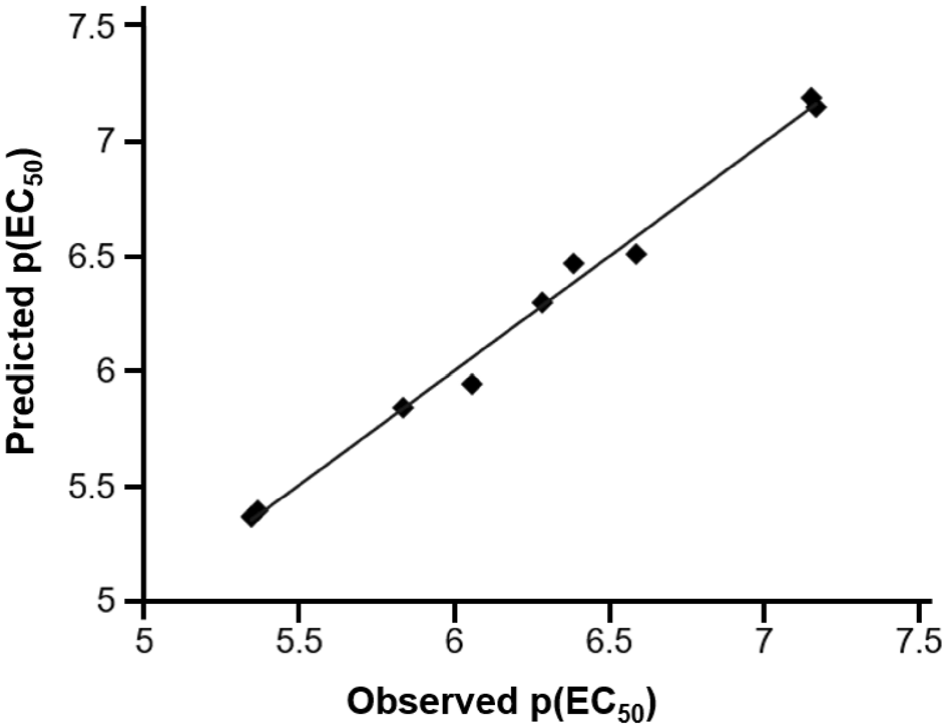
Plot between observed and predicted anti-dengue activities of flavone derivatives.

A semi-3D quantitative structure–activity relationship (QSAR) model of these flavone derivatives with their anti-dengue activities was generated via Materials Studio Program using the physicochemical parameters generated from ADMETlab 2.0^34^ as the descriptors. Multiple linear regression (MLR) was applied to manipulate the equation. After regression analysis, the best equation (plotted graph shown in Fig. 9) obtained was

1 $${\text{p}}\left( {{\text{EC}}_{{{5}0}} } \right) = 0.{7537}\left[ {{\text{iLOGP}}} \right] - {8}.0{753 }\left[ {\text{H - HT}} \right] - {1}.{4921 }\left[ {{\text{ROA}}} \right] + {5}.{4918}$$

with r^2^ = 0.993, r^2^(CV) = 0.976, Residual Sum of Squares = 0.027, Predictive Sum of Squares = 0.087.

The model shows excellent squared correlation coefficient (r^2^) of 0.993 between descriptors (iLOGP, H-HT, and ROA) and the anti-dengue activity. The exceptional cross-validated squared correlation coefficient of this model of 0.976 reflects the good internal prediction power of this model. From QSAR model, it is suggested that higher lipophilicity (iLOGP) of the flavone derivatives results in higher anti-dengue activity. Moreover, the predicted human hepatotoxicity (H-HT) and rat oral acute toxicity (ROA) are contributing negatively to the activity against DENV-2. This predicted model confirmed that these flavone analogs could be convenient for further drug development.

## Materials and methods

### Synthesis and identification of flavone analogs

All reagents and solvents were obtained from Sigma–Aldrich (St. Louis, MO, USA), TCI chemicals (Tokyo, Japan) and Merck (Darmstadt, Germany). All solvents for column chromatography from RCI Labscan (Samutsakorn, Thailand) were distilled before use. Reactions were monitored by thin–layer chromatography (TLC) using aluminium Merck TLC plates coated with silica gel 60 F254. Normal phase column chromatography was performed using silica gel 60 (0.063–0.200 mm, 70–230 mesh ASTM, Merck, Darmstadt, Germany). Proton and carbon nuclear magnetic resonance (^1^H and ^13^C NMR) spectra were recorded on a Jeol JNM–ECZ500/S1 (500 MHz). Chemical shifts were expressed in parts per million (ppm), *J* values were in Hertz (Hz). High-resolution mass spectra (HRMS) data were obtained with Micro-TOF mass spectrometer. IR spectra were recorded using the Thermo Scientific™ Nicolet™ iS50 FTIR spectrometer with ATR module and are reported in wave number (cm^−1^). Melting points were measured using a melting point apparatus (Griffin) and are uncorrected.

#### General procedure A^21^

A mixture of flavone, alkyl halide, K_2_CO_3_ and dry DMF or dry acetone was stirred at 60 °C overnight. After completion, the reaction was quenched with DI water and extracted with EtOAc (3 times). The combined organic layers were washed with brine, dried with anh. Na_2_SO_4_, filtered and concentrated in vacuo. The crude mixture was purified by silica gel column chromatography.

#### General procedure B^22^

A mixture of flavone, propionyl chloride, K_2_CO_3_ and dry DMF was stirred at room temperature overnight. The reaction was quenched with DI water and extracted with EtOAc (3 times). The combined organic layers were washed with brine, dried with anh. Na_2_SO_4_, filtered and concentrated in vacuo. The crude mixture was purified by silica gel column chromatography.

#### General procedure C^23^

A mixture of flavone, acid anhyhydride and pyridine was stirred at room temperature overnight. The reaction was quenched with DI water and extracted with CH_2_Cl_2_. The combined organic layers were washed with 10% NaOH, 1M HCl and brine, dried with anh. Na_2_SO_4_, filtered and concentrated in vacuo.

#### General procedure D^27^

BF_3_⋅Et_2_O was added into a mixture of cinnamoyl chloride and 3,4,5-trimethoxyphenol. After refluxing at 90 °C for an hour, the reaction was quenched with DI water and extracted with EtOAc (3 times). The combined organic layers were dried with anh. Na_2_SO_4_, filtered and concentrated in vacuo.

#### General procedure E^28^

The mixture of 6-hydroxy-2,3,4-trimethoxychalcone and I_2_ was dissolved in DMSO. After refluxing at 90 °C for 3 h, the reaction was quenched with DI water and extracted with CH_2_Cl_2_ (3 times). The combined organic layers were dried with anh. Na_2_SO_4_, filtered and concentrated in vacuo.

#### General procedure F^37^

To a solution of 5,6,7-trimethoxyflavone in CH_2_Cl_2_ at 0 ºC was added BBr_3_. After stirring at room temperature for 6 h, the reaction was quenched with 1 M HCl and extracted with EtOAc (3 times). The combined organic layers were dried with anh. Na_2_SO_4_, filtered and concentrated in vacuo.

#### General Procedure H^38^

To a solution of 5,6,7-trimethoxyflavone in toluene was added AlCl_3_. After stirring at 100 °C for 3 h, the reaction was quenched with 1M HCl and left stirring for an hour. The mixture was extracted with EtOAc (3 times). The combined organic layers were dried with anh. Na_2_SO_4_, filtered and concentrated in vacuo.

#### 6-Methoxyflavone (1b)

The title compound was synthesized following the General Procedure A using 6-hydroxyflavone (48 mg, 0.20 mmol), MeI (66 µL, 1.0 mmol), K_2_CO_3_ (140 mg, 1.0 mmol) and dry DMF (0.50 mL). The crude product was purified by silica gel column chromatography (eluent: EtOAc/hexanes = 1:2) to give to give a white powder of **1b** (45 mg, 89%). **TLC** (EtOAc:hexanes 2:1): R_f_ = 0.49; ^**1**^**H-NMR** (500 MHz, acetone-*d*6) δ 8.06 (dd, *J* = 7.1, 2.9, 2H), 7.67 (d, *J* = 9.6 Hz, 1H), 7.55–7.59 (m, 3H), 7.49 (d, *J* = 3.1 Hz, 1H), 7.36 (dd, *J* = 8.9, 2.9 Hz, 1H), 6.81 (s, 1H), 3.90 (s, 3H); ^**13**^**C-NMR** (126 MHz, acetone-*d*6) δ 176.9, 162.8, 157.2, 151.0, 132.0, 131.5, 129.1, 126.3, 124.7, 123.1, 119.9, 106.3, 105.0, 55.4. ^1^H and ^13^C data are consistent with literature values^39^.

#### 6-Propionoxyflavone (1c)

The title compound was synthesized following the General Procedure B using 6-hydroxyflavone (48 mg, 0.20 mmol), propionyl chloride (42 µL, 0.48 mmol), K_2_CO_3_ (56 mg, 0.40 mmol) and dry DMF (0.50 mL). The crude product was purified by silica gel column chromatography (eluent: EtOAc/hexanes = 1:1) to give a pale-yellow powder of **1c** (51 mg, 78%). **TLC** (EtOAc:hexanes 2:1): R_f_ = 0.27; ^**1**^**H-NMR** (500 MHz, DMSO-*d*6) δ 8.12 (dd, *J* = 5.7, 3.1 Hz, 2H), 7.88 (d, *J* = 9.9 Hz, 1H), 7.76 (d, *J* = 2.9 H, 1H), 7.64 (d, *J* = 2.3 Hz, 1H), 7.62–7.58 (m, 3H), 7.07 (s, 1H), 2.66 (q, *J* = 7.7 Hz, 2H), 1.16 (t, *J* = 7.2 Hz, 3H); ^**13**^**C-NMR** (126 MHz, DMSO-*d*_6_) δ 177.2, 173.2, 163.4, 153.7, 148.1, 132.5, 131.6, 129.7, 129.1, 127.0, 124.5, 120.7, 117.4, 107.0, 27.4, 9.3. ^1^H and ^13^C data are consistent with literature values^40^.

#### 7-Methoxyflavone (2b)

The title compound was synthesized following the General Procedure A using 7-hydroxyflavone (97 mg, 0.40 mmol), MeI (50 µL, 0.80 mmol), K_2_CO_3_ (111 mg, 0.80 mmol) and dry DMF (0.50 mL). The crude product was purified by silica gel column chromatography (eluent: EtOAc/hexanes = 2:1) to give a white powder of **2b** (102 mg, 99%). **TLC** (EtOAc:hexanes 2:1): R_f_ = 0.52; ^**1**^**H-NMR** (500 MHz, CDCl_3_) δ 8.14 (d, *J* = 8.7 Hz, 1H), 7.94–7.88 (m, 2H), 7.57–7.48 (m, 3H), 7.07–6.94 (m, 2H), 6.77 (s, 1H), 3.93 (s, 3H); ^**13**^**C-NMR** (126 MHz, CDCl_3_) δ 176.8, 164.3, 163.1, 158.1, 132.0, 131.5, 129.1, 127.2, 126.3, 118.0, 114.5, 107.7, 100.5, 56.0. ^1^H and ^13^C data are consistent with literature values^40^.

#### 7-Propionoxyflavone (2c)

The title compound was synthesized following the General Procedure B using 7-hydroxyflavone (96 mg, 0.40 mmol), propionyl chloride (84 µL, 0.96 mmol), K_2_CO_3_ (111 mg, 0.80 mmol) and dry DMF (0.50 mL). The crude product was purified by silica gel column chromatography (eluent: EtOAc/hexanes = 1:2) to give a white powder of **2c** (31 mg, 22%). **TLC** (EtOAc:hexanes 2:1): R_f_ = 0.24; ^**1**^**H-NMR** (500 MHz, acetone-*d6*) δ 8.10 (d, *J* = 8.6 Hz, 1H), 8.08–8.02 (m, 2H), 7.62–7.50 (m, 4H), 7.23 (d, *J* = 8.6 Hz, 1H), 6.82 (s, 1H), 2.66 (q, *J* = 7.5 Hz, 2H), 1.20 (t, *J* = 7.4 Hz, 3H). ^**13**^**C-NMR** (126 MHz, acetone-*d*_6_) δ 176.4, 171.9, 163.3, 156.7, 155.2, 131.7, 131.7 129.1, 126.4, 126.4, 121.6, 119.8, 111.5, 107.2, 27.2, 8.3. ^1^H and ^13^C data are consistent with literature values^22^.

#### 7,8-Dioxoloflavone (3b)

The title compound was synthesized following the General Procedure A using 7,8-dihydroxyflavone (51 mg, 0.20 mmol), dibromomethane (17 µL, 0.24 mmol), K_2_CO_3_ (138 mg, 1.0 mmol) and dry DMF (0.50 mL) at 100 °C. The crude product was purified by silica gel column chromatography (eluent: EtOAc/hexanes = 2:1) to give a pale-yellow powder of **3b** (38 mg, 71%). **TLC** (EtOAc:hexanes 2:1): R_f_ = 0.63; ^**1**^**H-NMR** (500 MHz, CDCl_3_) δ 7.92 (dd, *J* = 7.9, 2.2 Hz, 2H), 7.81 (d, *J* = 8.4 Hz, 1H), 7.49–7.56 (m, 3H), 6.96 (d, *J* = 8.4 Hz, 1H), 6.74 (s, 1H), 6.22 (s, 2H); ^**13**^**C-NMR** (126 MHz, CDCl_3_) δ 177.5, 162.7, 152.5, 141.3, 134.9, 131.7, 131.6, 129.1, 126.3, 120.3, 120.1, 107.2, 107.1, 103.3. ^1^H data are consistent with literature values^41^.

#### 5-Hydroxy-7-methoxyflavone (4b)

The title compound was synthesized following the General Procedure A using chrysin (51 mg, 0.20 mmol), MeI (63 µL, 1.0 mmol), K_2_CO_3_ (138 mg, 1.0 mmol) and anhydrous DMF (1.0 mL). The crude product was purified by silica gel column chromatography (eluent: EtOAc/hexanes = 1:9) to give a pale-yellow powder of **4b** (76 mg, 67%). **TLC** (EtOAc:hexanes 1:4): R_f_ = 0.21; ^**1**^**H-NMR** (500 MHz, CDCl_3_) δ 12.72 (br s, 1H), 7.86 (dd, *J* = 8.0 Hz, 2H), 7.56–7.48 (m, 3H), 6.64 (s, 1H), 6.48 (d, *J* = 2.3 Hz, 1H), 6.35 (d, *J* = 2.3 Hz, 1H), 3.87 (s, 3H); ^**13**^**C-NMR** (126 MHz, CDCl_3_) δ 182.5, 165.7, 164.0, 162.2, 157.8, 131.9, 131.3, 129.2, 126.4, 105.9, 105.8, 98.3, 92.7, 55.9. ^1^H and ^13^C data are consistent with literature values^42^.

#### 7-Benzyloxy-5-hydroxyflavone (4c)

The title compound was synthesized following the General Procedure A using chrysin (254 mg, 1.0 mmol), BnBr (178 µL, 1.5 mmol), K_2_CO_3_ (276 mg, 2.0 mmol) and acetone (10 mL). After the combined organic layers were concentrated in vacuo, a pale yellow powder of **4c** (310 mg, 90%) was obtained without further purification. **TLC** (EtOAc:hexanes 1:2): R_f_ = 0.60; ^**1**^**H-NMR** (500 MHz, CDCl_3_) δ 12.64 (br s, 1H), 7.88 (d, *J* = 6.5 Hz, 2H), 7.51–7.57 (m, 3H), 7.37–7.48 (m, 5H), 6.68 (s, 1H), 6.59 (d, *J* = 2.1 Hz, 1H), 6.46 (d, *J* = 2.2 Hz, 1H), 5.15 (s, 2H); ^**13**^**C-NMR** (126 MHz, CDCl_3_) δ 182.6, 164.7, 164.1, 162.3, 157.8, 135.8, 131.9, 131.4, 129.2, 128.8, 128.5, 127.6, 126.4, 106.0, 99.0, 93.6, 70.5. ^1^H and ^13^C data are consistent with literature values^43^.

#### 6,8-Dibromo-5,7-dihydroxyflavone (4d)

A mixture of chrysin (254 mg, 1.0 mmol), NBS (196 mg, 1.1 mmol), dry THF (4.0 mL) and conc. H_2_SO_4_ (50 µL) was stirred at 60 °C for 2 h. After completion, the reaction was quenched with DI water. The mixture was extracted with EtOAc (3 × 20 mL). The combined organic layers were washed with brine, dried with anh. Na_2_SO_4_, filtered and concentrated in vacuo. The crude mixture was purified by silica gel column chromatography (eluent: EtOAc/hexanes = 1:2) to give a light brown powder of **4d** (128 mg, 31%). **TLC** (EtOAc:hexanes 1:2): R_f_ = 0.17; ^**1**^**H-NMR** (500 MHz, DMSO-*d*6) δ 13.65 (br s, 1H), 10.97 (br s, 1H), 8.06 (d, *J* = 6.8 Hz, 2H), 7.57–7.50 (m, 3H), 7.13 (s, 1H); ^**13**^**C-NMR** (126 MHz, DMSO-*d*_6_) δ 182.2, 174.1, 164.1, 157.9, 157.6, 152.9, 133.1, 130.8, 129.9, 127.1, 105.7, 95.1, 89.0. ^1^H and ^13^C data are consistent with literature values^26^.

#### 5,7-Dihydroxy-8-nitroflavone (4e)

The mixture of chrysin (254 mg, 1.0 mmol), conc. HNO_3_ (0.10 mL), and glacial acetic acid (3.0 mL) was stirred at 60 °C for an hour. After completion, the reaction was quenched with DI water and kept at 0 °C. The precipitate was collected by suction filtration. The crude mixture was purified by silica gel column chromatography (eluent: EtOAc/hexanes = 2:1) to give a white powder of **4e** (72 mg, 24%). **TLC** (EtOAc:hexanes 2:1): R_f_ = 0.42; ^**1**^**H-NMR** (500 MHz, CDCl_3_) δ 12.02 (br s, 1H), 8.10 (d, *J* = 6.8 Hz, 2H), 7.57–7.64 (m, 3H), 6.92 (s, 1H), 6.49, (s, 1H); ^**13**^**C-NMR** (126 MHz, CDCl_3_) δ 186.7, 168.5, 167.9, 163.2, 155.2, 138.4, 135.2, 134.6, 131.7, 111.3, 108.4, 104.1, 101.6. ^1^H and ^13^C data are consistent with literature values^24^.

#### 8-Amino-5,7-dihydroxyflavone (4f)

A mixture of **4e** (154 mg, 0.26 mmol), Sn powder (309 mg, 2.6 mmol), 12 M HCl (10 mL) and EtOH (10 mL) was mixed at 0 °C and left stirring at room temperature for 72 h. After completion, the reaction was quenched with DI water. The mixture was neutralized by sat. NaHCO_3_(aq) and extracted with EtOAc (3 × 20 mL). The combined organic layers were washed with brine, dried with anh. Na_2_SO_4_, filtered and concentrated in vacuo to give a brown powder of **4f** (81 mg, 58%). **TLC** (EtOAc:hexanes 1:1): R_f_ = 0.35; ^**1**^**H-NMR** (500 MHz, DMSO-*d*_6_) δ 12.06 (br s, 1H), 8.21 (d, *J* = 7.2 Hz, 2H), 7.54–7.61 (m, 3H), 6.90 (s, 1H), 6.29 (s, 1H); ^**13**^**C-NMR** (126 MHz, DMSO-*d*_6_) δ 182.9, 163.5, 151.9, 151.8, 143.8, 132.5, 131.4, 129.6, 127.2, 116.9, 105.0, 104.1, 98.9. ^1^H and ^13^C data are consistent with literature values^44^.

#### 5-Hydroxy-6,7-dimethoxyflavone (5b)

The title compound was synthesized using the previously described method^21^. Light yellow solid; Yield: 19%; **TLC** (EtOAc:hexanes 3:2): R_f_ = 0.17; ^**1**^**H NMR** (500 MHz, acetone-*d*_6_): δ 12.80 (br s, 1H), 8.08–8.06 (m, 2H), 7.60–7.57 (m, 3H), 6.88 (s, 1H), 6.81 (s, 1H), 3.97 (s, 3H), 3.77 (s, 3H); ^**13**^**C NMR** (126 MHz, CDCl_3_): δ 182.9, 164.1, 159.0, 153.4, 153.1, 132.8, 132.0, 131.4, 129.2, 126.4, 106.4, 105.7, 90.7, 61.0, 56.4. ^1^H and ^13^C NMR data are consistent with literature values^21^.

#### 5,6,7-Triacetoxyflavone (5c)

The title compound was synthesized following the General Procedure C using baicalein (20 mg, 0.074 mmol), pyridine (60 µL), and acetic anhydride (2.2 mL). The reaction was stirred at room temperature for 4 h. The reaction was quenched with DI water and extracted with EtOAc (3 × 10 mL). The combined organic layers were concentrated in vacuo yielding a pale-yellow solid of **5c** (29 mg, 99%). **TLC** (EtOAc:hexanes 2:3): R_f_ = 0.26; ^**1**^**H NMR** (500 MHz, CDCl_3_): δ 7.84 (dd, *J* = 8.3, 1.8 Hz, 2H), 7.54–7.50 (m, 3H), 7.49 (s, 1H), 6.64 (s, 1H), 2.43 (s, 3H), 2.34 (s, 3H), 2.33 (s, 3H); ^**13**^**C NMR** (126 MHz, CDCl_3_): δ 176.3, 168.4, 167.3, 167.1, 162.9, 154.3, 147.0, 142.2, 131.9, 131.1, 129.3, 129.2, 126.3, 110.4, 108.3, 105.9, 20.9, 20.8, 20.2. ^1^H and ^13^C NMR data are consistent with literature values^23^.

#### 5,6,7-Tripropionoxyflavone (5d)

Propionic anhydride (2.4 mL, 15 mmol) was added into the solution of baicalein (1.0 g, 3.7 mmol) in CH_2_Cl_2_ (50 mL). NEt_3_ (2.0 mL, 3.7 mmol) was added into the mixture. After stirring at room temperature for 2 h, the reaction was quenched with DI water and extracted with CH_2_Cl_2_. The combined organic layers were washed with 10% NaOH, 1 M HCl and brine. The crude mixture was concentrated in vacuo to give an off-white solid of **5d** (1.3 g, 80%). **TLC** (EtOAc:hexanes 1:3): R_f_ = 0.21; ^**1**^**H NMR** (500 MHz, CDCl_3_): δ 7.84 (dd, *J* = 8.1, 1.5 Hz, 2H), 7.54–7.47 (m, 4H), 6.63 (s, 1H), 2.75 (q, *J* = 7.5 Hz, 2H), 2.61 (q, *J* = 7.6 Hz, 2H), 2.60 (q, *J* = 7.6 Hz, 2H), 1.32–1.25 (m, 9H); ^**13**^**C NMR** (126 MHz, CDCl_3_):δ 176.4, 171.8, 170.9, 170.7, 162.7, 154.2, 147.1, 142.3, 132.8, 131.9, 131.1, 129.2, 126.3, 115.7, 110.3, 108.3, 27.7, 27.6, 27.2 9.4, 9.0, 8.9. ^1^H and ^13^C NMR data are consistent with literature values^23^.

#### 5-Hydroxy-6,7-dipropionoxyflavone (5e)

To a solution of **5d** (200 mg, 0.46 mmol) in acetic acid (2.76 mL), conc. HNO_3_ (0.46 mL) was added dropwise at 0 °C. The reaction was stirred at 65 °C for 2 h, quenched with DI water and filtered. The residue was recrystallized in EtOH to give a pale-yellow solid of **5e** (105 mg, 60%). **m.p.**: 132–134 °C; **TLC** (EtOAc:hexanes 1:4): R_f_ = 0.21; ^**1**^**H NMR** (500 MHz, CDCl_3_): δ 12.91 (s, 1H), 7.88 (d, *J* = 7.1 Hz, 2H), 7.60–7.50 (m, 3H), 6.98 (s, 1H), 6.73 (s, 1H), 2.69–2.59 (m, 4H), 1.33–1.27 (m, 6H); ^**13**^**C NMR** (126 MHz, CDCl_3_): δ 178.8, 171.3, 171.1, 165.1, 158.11, 153.5, 153.4, 148.6, 132.4, 131.0, 129.3, 126.5, 111.5, 105.9, 101.7, 27.7, 27.2, 9.3, 9.1; **IR** (neat): 3017 (C–H), 2990 (C–H), 2847 (O–H), 1812 (C=O), 1793 (C=O) 1642 (C=O), 1560 (C=C), 1432 (C=C), 1315 (C=C), 1244 (C=C), 1159 (C–O), 1090, (C–O) cm^-1^; **HRMS** (m/z): [M + H]^+^ calcd. for C_21_H_19_O_7_, 383.1131; found 383.1135.

#### 8-Bromo-5,6,7-trihydroxyflavone (5f)

NBS (54 mg, 0.30 mmol) was added into a solution of baicalein (54 mg, 0.20 mmol) in THF (6.2 mL). The reaction was stirred at room temperature for 3 h, quenched with DI water and extracted with EtOAc (3 times). The combined organic layers were concentrated in vacuo to give a light-yellow solid of **5f** (65 mg, 93%). **TLC** (EtOAc:hexanes 1:3): R_f_ = 0.10; ^**1**^**H NMR** (500 MHz, acetone-*d*_6_): δ 12.82 (br s, 1H), 8.18 (dd, *J* = 7.6, 2.1 Hz, 2H), 7.67–7.62 (m, 3H), 6.90 (s, 1H); ^**13**^**C NMR** (126 MHz, acetone-*d*_6_): δ 183.0, 163.8, 150.8, 147.4, 146.6, 132.1, 131.3, 130.4, 129.3, 126.5, 105.3, 104.6, 86.6. ^1^H and ^13^C NMR data are consistent with literature values^45^.

#### 5,6,7-Triacetoxy-8-bromoflavone (5g)

The title compound was synthesized following the General Procedure C using **5f** (17.5 mg, 0.050 mmol), pyridine (40.5 µL), and acetic anhydride (1.5 mL) to give a pale beige solid of **5g** (15 mg, 63%). **TLC** (EtOAc:hexanes 1:1): R_f_ = 0.24; ^**1**^**H NMR** (500 MHz, CDCl_3_): δ 7.95 (dd, *J* = 8.1, 1.5 Hz, 2H), 7.57–7.51 (m, 3H), 6.70 (s, 1H), 2.42 (s, 3H), 2.41 (s, 3H), 2.34 (s, 3H); ^**13**^**C NMR** (126 MHz, CDCl_3_): δ 181.1, 169.5, 165.5, 169.2, 162.8, 151.7, 151.0, 141.5, 133.2, 132.3, 129.4, 129.3, 126.5, 112.4, 105.6, 104.6, 20.9, 20.4, 20.1. ^1^H and ^13^C NMR data are consistent with literature values^46^.

#### 8-Bromo-5,6,7-tripropionoxyflavone (5h)

The title compound was synthesized following the General Procedure C using **5f** (94 mg., 0.27 mmol), pyridine (22 µL, 0.27 mmol), and propionic anhydride (1.1 mL, 8.3 mmol) to give a pale beige solid of **5h** (43 mg, 59%). **m.p.** 113–115 ºC; **TLC** (EtOAc:hexanes = 2:3): R_f_ = 0.18; ^**1**^**H NMR** (500 MHz, CDCl_3_): δ 8.01–7.90 (m, 2H), 7.61–7.47 (m, 3H), 6.70 (s, 1H), 2.78–2.65 (m, 4H), 2.60 (q, *J* = 7.4 Hz, 2H), 1.39–1.24 (m, 9H).; ^**13**^**C NMR** (126 MHz, CDCl_3_): δ 176.0, 171.4, 170.5, 169.8, 162.7, 151.7, 146.5, 141.6, 134.3, 132.2, 130.7, 129.3, 126.5, 116.5, 108.0, 105.3, 27.6, 27.4, 27.2, 9.4, 9.2, 8.9; **IR** (neat): 3059 (C–H), 1817 (C=O), 1803 (C=O), 1704 (C=O), 1630 (C=C) 1415 (C=C), 1357 (C=C), 1165 (C–O), 1121 (C–O), 1075 (C–O) cm^-1^; **HRMS** (m/z): [M + H]^+^ calcd. for C_24_H_22_Br^79^O_8_, 517.0498; found 517.0490.

#### 5,6,7-Trimethoxyflavone (5i)

General Procedure D was followed using BF_3_⋅Et_2_O (5.0 mL), cinnamoyl chloride (866 mg, 5.0 mmol) and 3,4,5-trimethoxyphenol (958 mg, 5.0 mmol) to give an orange-brown solid of 6-hydroxy-2,3,4-trimethoxychalcone (**6i**, 2.7 g, 100%). Then, the title compound was synthesized following General Procedure E using **6i** (314 mg, 1.0 mmol) and I_2_ (20 mg, 0.080 mmol) and DMSO (12 mL) to give a dark yellow solid of **5i** (126 mg, 40%). **TLC** (EtOAc:hexanes 1:1): R_f_ = 0.28; ^**1**^**H NMR** (500 MHz, CDCl_3_): δ 7.86 (dd, *J* = 7.9, 1.8 Hz, 2H), 7.51–7.47 (m, 3H), 6.80 (s, 1H), 6.66 (s, 1H), 3.98 (s, 3H), 3.97 (s, 3H), 3.90 (s, 3H); ^**13**^**C NMR** (126 MHz, CDCl_3_): δ 177.1, 160.1, 158.0, 154.5, 152.7, 140.6, 132.4, 130.6, 127.5, 126.0, 113.0, 108.6, 96.3, 62.3, 61.7, 56.4. ^1^H and ^13^C NMR data are consistent with literature values^47^.

#### 4′-Bromo-5,6,7-trimethoxylflavone (5j)

General Procedure D was followed using BF_3_⋅Et_2_O (8.2 mL), 4′-bromocinnamoyl chloride (1.23 g, 5.0 mmol) and 3,4,5-trimethoxyphenol (958 mg, 5.0 mmol) to give a dark-orange solids of 4′-bromo-6-hydroxy-2,3,4-trimethoxychalcone (**6j**, 702 mg, 36%). Then, the title compound was synthesized following General Procedure E using **6j** (650 mg, 1.7 mmol), I_2_ (34 mg, 0.14 mmol) and DMSO (18 mL) to give a dark green solid of **5j** (419 mg, 65%). **TLC** (EtOAc:hexanes 1:1): R_f_ = 0.27; ^**1**^**H NMR** (500 MHz, CDCl_3_): δ 7.72 (d, *J* = 8.8 Hz, 2H), 7.62 (d, *J* = 8.8 Hz, 2H), 6.79 (s, 1H), 6.63 (s, 1H), 3.97 (s, 6H), 3.90 (s, 3H); ^**13**^**C NMR** (126 MHz, CDCl_3_): δ 177.1, 160.1, 158.0, 154.5, 152.7, 140.6, 132.4, 130.6, 127.5, 126.0, 113.0, 108.6, 96.3, 62.3, 61.7, 56.4. ^1^H and ^13^C NMR data are consistent with literature values^28^.

#### 5,6,7-Trimethoxy-4′-nitroflavone (5k)

General Procedure D was followed using BF_3_⋅Et_2_O (7.4 mL), 4′-nitrocinnamoyl chloride (1.3 g, 6.2 mmol) and 3,4,5-trimethoxyphenol (1.14 g, 6.2 mmol) to give an orange-brown solid of 6-hydroxy-2,3,4-trimethoxy-4′-nitrochalcone (**6k**, 675 mg, 31%). Then, the title compound was synthesized following General Procedure E using **6k** (620 mg, 1.7 mmol), I_2_ (34 mg, 0.14 mmol) and DMSO (18 mL) to give a dark orange solid of **5k** (366 mg, 60%). **TLC** (EtOAc:hexanes 1:1): R_f_ = 0.31; ^**1**^**H NMR** (500 MHz, CDCl_3_): δ 8.35 (d, *J* = 8.6 Hz, 2H), 8.04 (d, *J* = 8.7 Hz, 2H), 6.82 (s, 1H), 6.75 (s, 1H), 3.99 (s, 3H), 3.98 (s, 3H), 3.92 (s, 3H); ^**13**^**C NMR** (126 MHz, CDCl_3_): δ 176.8, 162.9, 158.5, 154.5, 152.7, 149.3, 140.9, 137.6, 128.9, 123.5, 113.1, 110.6, 96.3, 62.3, 61.7, 56.5. ^1^H and ^13^C NMR data are consistent with literature values^47^.

#### 4′-Amino-5,6,7-trimethoxyflavone (5l)

To a solution of **5k** (60 mg, 0.17 mmol) in EtOH (2.7 mL) at 0 °C was slowly added 12 M HCl (2.7 mL), followed by Sn powder (100 mg, 0.84 mmol). The reaction was stirred at room temperature for 1.5 h, quenched with sat. NaHCO_3_ and extracted with EtOAc (3 times). The combined organic layers were concentrated in vacuo to give a dark orange solid of **5l** (54 mg, 100%). **TLC** (5% MeOH in CH_2_Cl_2_): R_f_ = 0.32; ^**1**^**H NMR** (500 MHz, CDCl_3_): δ 7.68 (d, *J* = 8.6 Hz, 2H), 6.76 (s, 1H), 6.73 (d, *J* = 8.6 Hz, 2H), 6.52 (s, 1H), 3.97 (s, 3H), 3.96 (s, 3H), 3.90 (s, 3H); ^**13**^**C NMR** (126 MHz, CDCl_3_): δ 177.4, 161.8, 157.5, 154.5, 152.6, 149.6, 140.3, 127.7, 121.1, 114.8, 112.9, 106.0, 96.3, 62.3, 61.6, 56.3. ^1^H and ^13^C NMR data are consistent with literature values^47^.

#### 4′-Bromo-5,6,7-trihydroxyflavone (5m)

The title compound was synthesized following the General Procedure F using **5j** (50 mg, 0.13 mmol), CH_2_Cl_2_ (1.0 mL), and BBr_3_ (36 µL, 0.38 mmol) to give a dark green solid of **5m** (42 mg, 94%). **TLC** (5% MeOH in CH_2_Cl_2_): R_f_ = 0.10; ^**1**^**H NMR** (500 MHz, DMSO-*d*_6_): δ 8.01 (d, *J* = 8.6 Hz, 2H), 7.77 (d, *J* = 8.6 Hz, 2H), 6.98 (s, 1H), 6.62 (s, 1H); ^**13**^**C NMR** (126 MHz, DMSO-*d*_6_): δ 182.6, 162.3, 154.2, 150.3, 147.4, 132.7, 130.7, 129.9, 128.8, 126.1, 105.3, 104.8, 94.6. ^1^H and ^13^C NMR data are consistent with literature values^27^.

#### 5,6,7-Trihydroxy-4′-nitroflavone (5n)

The title compound was synthesized following the General Procedure F using **5k** (20 mg, 0.056 mmol), CH_2_Cl_2_ (1 mL), and BBr_3_ (50 µL, 0.53 mmol) to give a dark orange solid of **5n** (13 mg, 74%). **TLC** (5% MeOH in CH_2_Cl_2_): R_f_ = 0.06; ^**1**^**H NMR** (500 MHz, DMSO-*d*_6_): δ 12.45 (s, 1H), 10.69 (s, 1H), 8.88 (s, 1H), 8.35 (d, *J* = 7.1 Hz, 2H), 8.30 (d, *J* = 6.9 Hz, 2H), 7.11 (s, 1H), 6.62 (s, 1H); ^**13**^**C NMR** (126 MHz, DMSO-*d*_6_): δ 184.1, 162.6, 158.7, 154.3, 151.3, 147.5, 145.2, 136.5, 128.0, 124.6, 107.5, 104.9, 94.7. ^1^H and ^13^C NMR data are consistent with literature values^48^.

#### 4′-Amino-5,6,7-trihydroxyflavone (5o)

The title compound was synthesized following the General Procedure F using **5l** (40 mg, 0.12 mmol), CH_2_Cl_2_ (1.0 mL), and BBr_3_ (50 µL, 0.53 mmol) to give a yellow solid of **5o** (30 mg, 86%). **m.p.** > 250 ºC (decompose); **TLC** (5% MeOH in CH_2_Cl_2_): R_f_ = 0.28; ^**1**^**H NMR** (500 MHz, DMSO-*d*_6_): δ 7.70 (d, *J* = 9.0 Hz, 2H), 6.72 (d, *J* = 8.9 Hz, 2H), 6.53 (s, 1H), 6.48 (s, 1H); ^**13**^**C NMR** (126 MHz, DMSO-*d*_6_): δ 182.3, 167.5, 158.9, 154.0, 150.0, 147.3, 143.2, 128.5, 123.4, 114.0, 106.2, 104.3, 94.2; **IR** (neat): 3512 (O–H), 3497 (N–H), 3479 (N–H), 3077 (C–H), 2922 (C–H), 2879 (O–H), 1678 (C=O), 1497 (C=C), 1470 (C=C), 1381 (C=C), 1336 (C=C) cm^−1^; **HRMS** (m/z): [M + H]^+^ calcd. for C_15_H_12_NO_5_, 286.0715; found 286.0719.

#### 4′-Bromo-5-hydroxy-6,7-dimethoxyflavone (5p)

The title compound was synthesized following the General Procedure H using **5j** (30 mg, 0.077 mmol), toluene (5.6 mL), and AlCl_3_ (52 mg, 0.39 mmol) to give a brown solid of **5p** (63 mg, 100%). **TLC** (5% MeOH in CH_2_Cl_2_): R_f_ = 0.70; ^**1**^**H NMR** (500 MHz, CDCl_3_): δ 7.75 (d, *J* = 8.7 Hz, 2H), 7.65 (d, *J* = 8.7 Hz, 2H), 6.65 (s, 1H), 6.55 (s, 1H), 3.96 (s, 3H), 3.92 (s, 3H); ^**13**^**C NMR** (126 MHz, CDCl_3_): δ 182.7, 162.9, 159.1, 153.3, 153.1, 132.9, 132.5, 130.3, 127.8, 126.7, 106.4, 105.9, 90.8, 61.0, 56.5. ^1^H and ^13^C NMR data are consistent with literature values^27^.

#### 5-Hydroxy-6,7-dimethoxy-4′-nitroflavone (5q)

The title compound was synthesized following the General Procedure H using **5k** (30 mg, 0.092 mmol), toluene (6.7 mL), and AlCl_3_ (61 mg, 0.46 mmol) to give a brown solid of **5q** (59 mg, 81%). **m.p.** 145–150 ºC; **TLC** (2% MeOH in CH_2_Cl_2_): R_f_ = 0.59; ^**1**^**H NMR** (500 MHz, CDCl_3_): δ 8.38 (d, *J* = 9.0 Hz, 2H), 8.07 (d, *J* = 8.9 Hz, 2H), 6.77 (s, 1H), 6.59 (s, 1H), 3.98 (s, 3H), 3.93 (s, 3H); ^**13**^**C NMR** (126 MHz, CDCl_3_): δ 182.4, 161.1, 159.5, 157.6, 155.7, 153.1, 137.2, 133.1, 127.3, 124.4, 107.9, 104.1, 90.9, 61.0, 56.6; **IR** (neat): 3442 (O–H), 3013 (C–H), 2988 (C–H), 1697 (C = O), 1586 (N–O), 1449 (C = C), 1360 (C = C), 1345 (N–O), 1244 (C = C) cm^-1^; **HRMS** (m/z): [M + H]^+^ calcd. for C_17_H_14_NO_7_, 344.0770; found 344.0779.

#### 5,6-Dihydroxy-7-methoxy-4′-nitroflavone (5r)

To a solution of **5q** (30 mg, 0.087 mmol) in acetic acid (1.36 mL) was added 47% HBr (0.68 mL). After refluxing at 120 ºC for 3 h, the mixture was quenched with sat. NaHCO_3_ and extracted with EtOAc (3 times). The combined organic layers were concentrated in vacuo to give a dark brown solid of **5r** (25 mg, 89%). **m.p.** 174–178 ºC; **TLC** (EtOAc:hexanes 1:1): R_f_ = 0.25; ^**1**^**H NMR** (500 MHz, CDCl_3_): δ 8.37 (d, *J* = 8.9 Hz, 2H), 8.06 (d, *J* = 8.9 Hz, 2H), 6.77 (s, 1H), 6.65 (s, 1H), 4.02 (s, 3H); ^**13**^**C NMR** (126 MHz, CDCl_3_): δ 181.7, 162.3, 160.2, 152.6, 151.6, 147.4, 135.3, 129.1, 127.3, 123.4, 107.1, 103.6, 95.4, 64.0; **IR** (neat): 3454 (O–H), 3045 (C–H), 2950 (C–H), 2935 (O–H), 1684 (N–O), 1651 (C=O), 1604 (C=C), 1451 (C=C), 1342 (N–O), 1324 (C=C) cm^−1^; **HRMS** (m/z): [M + H]^+^ calcd. for C_16_H_12_NO_7_, 330.0614; found 330.0610.

### Anti-dengue activity and cellular toxicity

#### Cells and viruses

The cell lines of LLC/MK2 (ATCC®CCL-7) and C6/36 (ATCC®CRL-1660) were maintained in minimal essential medium (MEM) (Gibco®, Langley, USA) supplemented with 10% fetal bovine serum (Gibco®, Langley, USA); 100 I.U./mL penicillin, and 100 μg/mL streptomycin (Bio Basic Canada, Ontario, Canada); 10 mM HEPES (4-(2-hydroxyethyl)-1-piperazine-ethane-sulfonic acid) (Sigma Aldrich, St. Louis, USA) at 37 °C under condition of 5% CO_2_ and 28 °C, respectively^18^. Reference strain of DENV2 (New Guinea C strain, accession number NC_001474.2) was propagated in C6/36 and LLC/MK2 cell line with MEM medium added with 1% FBS, 100 I.U./mL penicillin, 100 μg/mL streptomycin, and 10 mM HEPES at 37 °C in 5% CO_2_ incubator^18^.

#### Screening antiviral efficacy

LLC/MK2 cells (5 × 10^4^) were seeded in 24-well plate and incubate at 37 °C under 5% CO_2_ overnight. Cells were infected with DENV2 NGC at a multiplicity of infection (M.O.I. of 0.1) and compounds at 10 µM were added and DMSO at 1% was used as control. Cultured plates were incubated for 1 h with gentle rocking every 15 min. Cells were washed with PBS, MEM supplemented with 1% FBS, 100 I.U./mL penicillin, and 100 µg/mL streptomycin, 10 mM HEPES was added in a presence of a compound at 10 µM. Cells were incubated at 37 °C with 5% CO_2_ for 3 days. Supernatants were collected to determine viral titers by plaque titration assay as previously described^49^. The selected compounds were further analyzed for an effective concentration (EC_50_).

#### Screening cellular toxicity

Cytotoxicity of the compounds was also accessed at the concentration of 10 µM in parallel with the viral inhibition screening. LLC/MK2 cells (1 × 10^4^) were seeded in 96-well plate and incubated at 37 °C under 5% CO_2_ overnight; compounds were added after 24 h, and then incubated for 2 days. DMSO at 1% was used as mock treatment. Cytotoxicity was measured using CellTiter 96® Aqueous One Solution Cell Proliferation Assay (MTS) kit (Promega, Wisconsin-Madison, WI, USA) according to the manufacturer’s instruction and analyzed by EnSight Multimode Plate Reader spectrophotometry at *A*_450nm_. (Perkin Elmer, Waltham, MA, USA).

#### Antiviral efficacy

LLC/MK2 (ATCC® CCL-7), and C6/36 (ATCC® CRL-1660) cell lines were propagated and maintained as previously described^18,29,31^. Effective concentration (EC_50_) of the compounds against the DENV2 were tested using LLC/MK2 cells^18,29^. Briefly, cells were seeded overnight and infected with each virus at the multiplicity of infection (M.O.I.) of 0.1 for 1 h. The compound was added during and after infection, and cells were incubated for 72 h. Supernatants were collected for plaque titration^31^. EC_50_ results were means and standard errors of three independent experiments.

### Molecular docking studies

To predict the possible viral protein target for flavone analogs, the crystal structures of dengue viral proteins were retrieved from the protein databank as follows; envelope (E) protein, the allosteric site of NS2B/NS3 protease (NS2B/NS3 pro), the SAM binding site of NS5 methyltransferases (NS5 MTase), and NS5 RNA-dependent RNA polymerase (NS5 RdRp), with PDB entry 1OKE^18^, 3U1I^50^, 6KR2^51^, and 3VWS^52^, respectively. The native inhibitors for E protein, NS2B/NS3 pro, NS5 MTase, and NS5 RdRp are 3-100-22^53^, SYC-1307^54^, Sinefungine^55^, and NITD-107^52^ were used as a reference data for the molecular docking study. The protein structures were considered a receptor prepared as a standard protocol^29^. The native inhibitors and ligands selected based on the antiviral efficacy were constructed and optimized using Gaussview 6 and Gaussian 16 at HF/6-31g* basis set^56^. The binding energy and pose were predicted using Autodock VinaXB^57^. The respective reference ligands were redocked to their crystal structure to validate the molecular docking protocol. The best interaction energy score (kcal/mol) of each flavone analog was ranked and plotted versus the native inhibitor. By considering each protein target of DENV, the binding affinity difference (Δ*D*) between native ligand (*D*_o_) and flavone analog (*D*_i_) calculated from Autodock VinaXB called likelihood ratios was applied to determine the potent compound (Eq. 2). The promising interaction of a ligand and its target with a greater ΔD value suggested a more optimistic interaction of ligand. The binding pose and interaction were visualized using the UCSF ChimeraX^58^ and BIOVIA Discovery Studio Visualizer V21.1.0^59^.

2 $$\Delta D = D_{{\text{o}}} - D_{{\text{i}}}$$

### Physicochemical properties prediction

A total of 33 flavone analogs were analyzed in terms of physicochemical descriptors, drug-likeness, and the ADME properties, which are absorption, distribution, metabolism, and excretion, using the SwissADME webserver^33^ and ADMETlab 2.0^34^. The Lipinski parameters are commonly used to estimate the drug-likeness properties by considering the following criteria; molecular weight ≤ 500, ≤ 5 hydrogen bond donors (HBDs), ≤ 10 hydrogen bond acceptors (HBAs), ≤ 5 = log of octanol to water partition coefficient (Log P), and the number of rotational bonds ≤ 5^35^.

### QSAR moodelling

The optimized structures of all flavone analogs and 36 descriptors obtained from ADMETlab were imported into the QSAR module of Materials Studio software to derive the relationship between physicochemical properties and anti-dengue activities. First, it should be noted that the Genetic Algorithm program in Materials Studio software was applied to select the significant descriptors. Next, the correlation coefficients between each pair of descriptors were calculated to avoid the overfitting equation. Finally, multiple linear regression was applied to obtain the QSAR models.

## Conclusions

In summary, 33 flavone analogs with various substitutions on both A and B rings were successfully synthesized. Many analogs showed remarkable anti-dengue activity against DENV-2 with the lowest EC_50_ of 70 and 68 nM for **5d** and **5e**, respectively, surpassing that of 8-bromobaicalein (**5f**), the most active flavone analog known to date, by over tenfold. The SAR analysis revealed the importance of the ester functional groups and also encourages further exploration on the other substitution patterns on the B ring of the flavone. Moreover, the QSAR model was successfully generated with the exceptional regressive performance (r^2^ = 0.993). The non-toxicity towards normal cell and the acceptable predicted physicochemical properties and drug-likeliness of these analogs suggest that they could exhibit high selectivity with good safety and pharmacokinetic profile for further development. The likelihood ratios from the docking study suggested that NS5 MTase and NS5 RdRp could be the potential targets for these analogs. With further confirmation of the mechanism of action experimentally, these targets could be used for rational design of flavone-based drug development in the future.

## Supplementary Information

Supplementary Information.

## Data Availability

The datasets generated and/or analyzed during the current study are available in the supplementary file. The dengue protein in this study are available in the RCSB protein data bank (https://www.rcsb.org/). AutoDock Vina XB (https://github.com/sirimullalab/vinaXB) was used for virtual screening by molecular docking technique. The molecular visualization and compound structural construction were performed using Chimera USF (https://www.cgl.ucsf.edu/chimera/) and VMD 1.9.3 (https://www.ks.uiuc.edu/Risearch/vmd/), which is free for academic users. Gnuplot (http://www.gnuplot.info) and adobe illustration 25.4.1 (https://www.adobe.com/products/illustrator.html) were used for plotting data and graphical visualization. The simplified molecular-input line-entry system (SMILES) of 33 flavone analogs is provided in the supplementary material. Scripts for data analysis and others are available from the authors upon request.
