# Supplementary material for: Design, synthesis, in vitro, in silico, and SAR studies of flavone analogs towards anti-dengue activity
Source: Sci Rep. 2022 Dec 14;12:21646. doi: 10.1038/s41598-022-25836-5 (PMC9751290; doi:10.1038/s41598-022-25836-5)
Supplement: Supplementary file 1 — Supplementary Information. [file 41598_2022_25836_MOESM1_ESM.pdf]

Supporting Information

Design, Synthesis, *In vitro*, *In silico*, and SAR Studies of Flavone Analogs towards Anti-Dengue Activity

Apinya Patigo[1], Kowit Hengphasatporn[2], Van Cao[3,4,5], Wattamon Paunrat[3,6], Natthanan Vijara[1], Thamonwan Chokmahasarn[1], Phornphimon Maitarad[7], Thanyada Rungrotmongkol[8,9], Yasuteru Shigeta[2], Siwaporn Boonyasuppayakorn[3], Tanatorn Khotavivattana[1,*]

[1] *Center of Excellence in Natural Products Chemistry, Department of Chemistry, Faculty of Science, Chulalongkorn University, Bangkok, 10330, Thailand*

[2] *Center for Computational Sciences, University of Tsukuba, 1-1-1 Tennodai, Tsukuba, Ibaraki 305-8577, Japan*

[3] *Center of Excellence in Applied Medical Virology, Department of Microbiology, Faculty of Medicine, Chulalongkorn University, Bangkok, 10330, Thailand*

[4] *Interdisciplinary Program in Microbiology, Graduate School, Chulalongkorn University, Bangkok 10330, Thailand*

[5] *DaNang University of Medical Technology and Pharmacy, DaNang 50200, Vietnam*

[6] *Medical Sciences Program, Faculty of Medicine, Chulalongkorn University, Bangkok 10330, Thailand*

[7] *Research Center of Nano Science and Technology, Department of Chemistry, College of Sciences, Shanghai University, Shanghai 200444, PR China*

[8] *Program in Bioinformatics and Computational Biology, Graduate school, Chulalongkorn University, Bangkok, 10330, Thailand*

[9] *Center of Excellence in Structural and Computational Biology, Department of Biochemistry, Faculty of Science, Chulalongkorn University, Bangkok, 10330, Thailand*

***Corresponding authors** tanatorn.k@chula.ac.th

Content

1. Likelihood ratios of selected compounds S1

2. The simplified molecular-input line-entry system (SMILES) S2

3. NMR and HRMS spectra S3

1. Likelihood ratios of selected compounds

Table S1 Likelihood ratios (ΔD) and molecular docking result (kcal/mol) of flavone analog (D_i) and native inhibitor (D_o) calculated from Autodock VinaXB

	E protein (kl loop)		E protein (Y site)		NS2B/NS3 pro		NS5 MTase		NS5 RdRp	
	D_i	ΔD	D_i	ΔD	D_i	ΔD	D_i	ΔD	D_i	ΔD
5a*	-7.4	-1.35	-7.1	-1.55	-6.3	-1.7	-9	0.2	-8.5	0.9
5c	-7.1	-1.65	-7.0	-1.65	-7.2	-0.8	-9.1	0.3	-8.0	0.4
5d	-7.0	-1.75	-7.1	-1.55	-7.6	-0.4	-9.2	0.4	-8.1	0.5
5e	-7.3	-1.45	-7.3	-1.35	-6.7	-1.3	-9.2	0.4	-7.8	0.2
5f*	-6.8	-1.95	-7.2	-1.45	-6.9	-1.1	-9.4	0.6	-8.8	1.2
5g	-6.7	-2.05	-6.2	-2.45	-7.3	-0.7	-8.5	-0.3	-7.9	0.3
5m	-8.3	-0.45	-7.3	-1.35	-7.4	-0.6	-8.8	0	-8.1	0.5
5n	-8.1	-0.65	-7.4	-1.25	-6.6	-1.4	-8.7	-0.1	-8.0	0.4
5q	-7.6	-1.15	-7.5	-1.15	-7.0	-1.0	-8.9	0.1	-7.8	0.2
Native inhibitor (D_o)	-8.75		-8.65		-8		-8.8		-7.6	

* Note that the molecular result of **5a** and **5f** were obtained from our previous study [19]

2. The simplified molecular-input line-entry system (SMILES)

Table S2 The simplified molecular-input line-entry system (SMILES) of 33 flavone analogs

Comp.	SMILES
**1a**	O=c1cc(-c2ccccc2)oc2ccc(O)cc12
**1b**	COc1ccc2oc(-c3ccccc3)cc(=O)c2c1
**1c**	CCC(=O)Oc1ccc2oc(-c3ccccc3)cc(=O)c2c1
**2a**	O=c1cc(-c2ccccc2)oc2cc(O)ccc12
**2b**	COc1ccc2c(=O)cc(-c3ccccc3)oc2c1
**2c**	CCC(=O)Oc1ccc2c(=O)cc(-c3ccccc3)oc2c1
**3a**	O=c1cc(-c2ccccc2)oc2c(O)c(O)ccc12
**3b**	O=c1cc(-c2ccccc2)oc2c3c(ccc12)OCO3
**4a**	O=c1cc(-c2ccccc2)oc2cc(O)cc(O)c12
**4b**	COc1cc(O)c2c(=O)cc(-c3ccccc3)oc2c1
**4c**	O=c1cc(-c2ccccc2)oc2cc(OCc3ccccc3)cc(O)c12
**4d**	O=c1cc(-c2ccccc2)oc2c(Br)c(O)c(Br)c(O)c12
**4e**	O=c1cc(-c2ccccc2)oc2c([N+](=O)[O-])c(O)cc(O)c12
**4f**	Nc1c(O)cc(O)c2c(=O)cc(-c3ccccc3)oc12
**5a**	O=c1cc(-c2ccccc2)oc2cc(O)c(O)c(O)c12
**5b**	COc1cc2oc(-c3ccccc3)cc(=O)c2c(O)c1OC
**5c**	CC(=O)Oc1cc2oc(-c3ccccc3)cc(=O)c2c(OC(C)=O)c1OC(C)=O
**5d**	CCC(=O)Oc1cc2oc(-c3ccccc3)cc(=O)c2c(OC(=O)CC)c1OC(=O)CC
**5e**	CCC(=O)Oc1cc2oc(-c3ccccc3)cc(=O)c2c(O)c1OC(=O)CC
**5f**	O=c1cc(-c2ccccc2)oc2c(Br)c(O)c(O)c(O)c12
**5g**	CC(=O)Oc1c(OC(C)=O)c(OC(C)=O)c2c(=O)cc(-c3ccccc3)oc2c1Br
**5h**	CCC(=O)Oc1c(OC(=O)CC)c(OC(=O)CC)c2c(=O)cc(-c3ccccc3)oc2c1Br
**5i**	COc1cc2oc(-c3ccccc3)cc(=O)c2c(OC)c1OC
**5j**	COc1cc2oc(-c3ccc(Br)cc3)cc(=O)c2c(OC)c1OC
**5k**	COc1cc2oc(-c3ccc([N+](=O)[O-])cc3)cc(=O)c2c(OC)c1OC
**5l**	COc1cc2oc(-c3ccc(N)cc3)cc(=O)c2c(OC)c1OC
**5m**	O=c1cc(-c2ccc(Br)cc2)oc2cc(O)c(O)c(O)c12
**5n**	O=c1cc(-c2ccc([N+](=O)[O-])cc2)oc2cc(O)c(O)c(O)c12
**5o**	Nc1ccc(-c2cc(=O)c3c(O)c(O)c(O)cc3o2)cc1
**5p**	COc1cc2oc(-c3ccc(Br)cc3)cc(=O)c2c(O)c1OC
**5q**	COc1cc2oc(-c3ccc([N+](=O)[O-])cc3)cc(=O)c2c(O)c1OC
**5r**	COc1cc2oc(-c3ccc([N+](=O)[O-])cc3)cc(=O)c2c(O)c1O
**6a**	Cc1ccc2oc(-c3ccccc3)cc(=O)c2c1

3. NMR and HRMS spectra

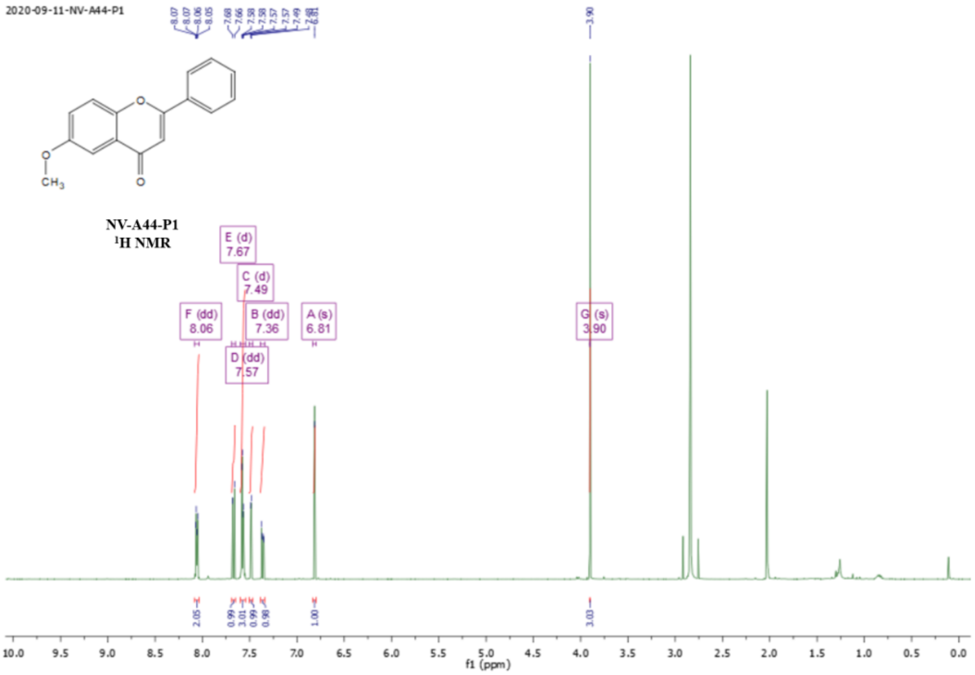

Figure S1 ^{1}H NMR spectrum of **1b**

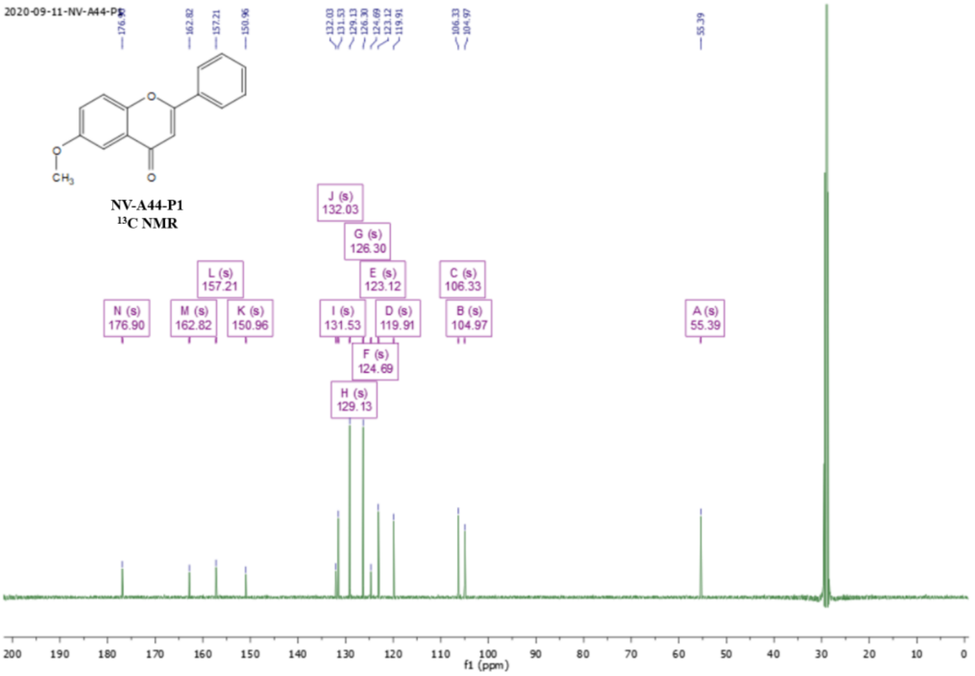

Figure S2 ^{13}C NMR spectrum of **1b**

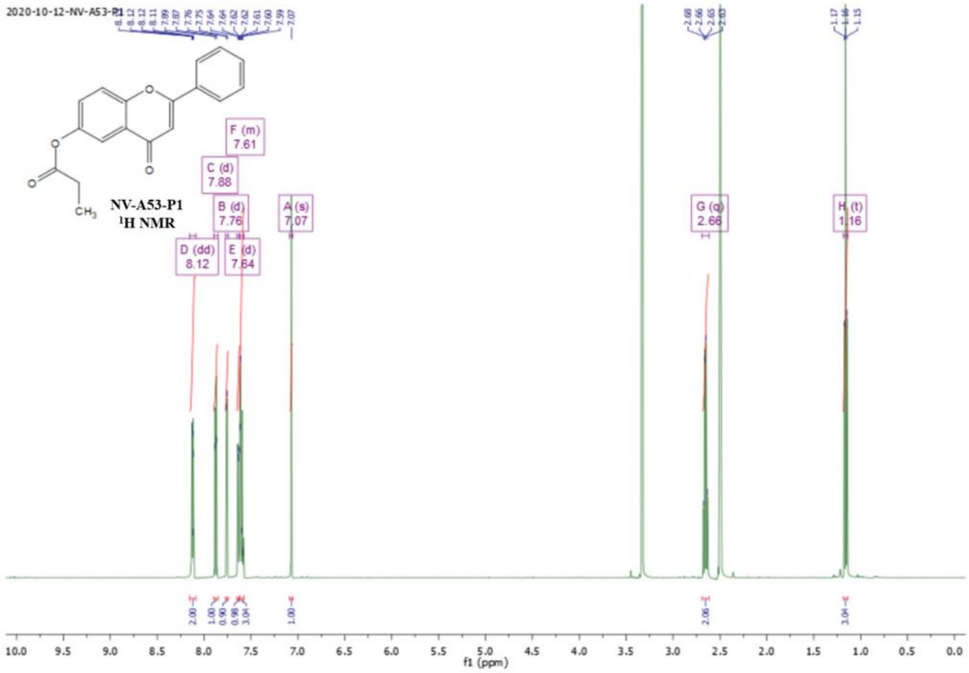

Figure S3 ^{1}H NMR spectrum of **1c**

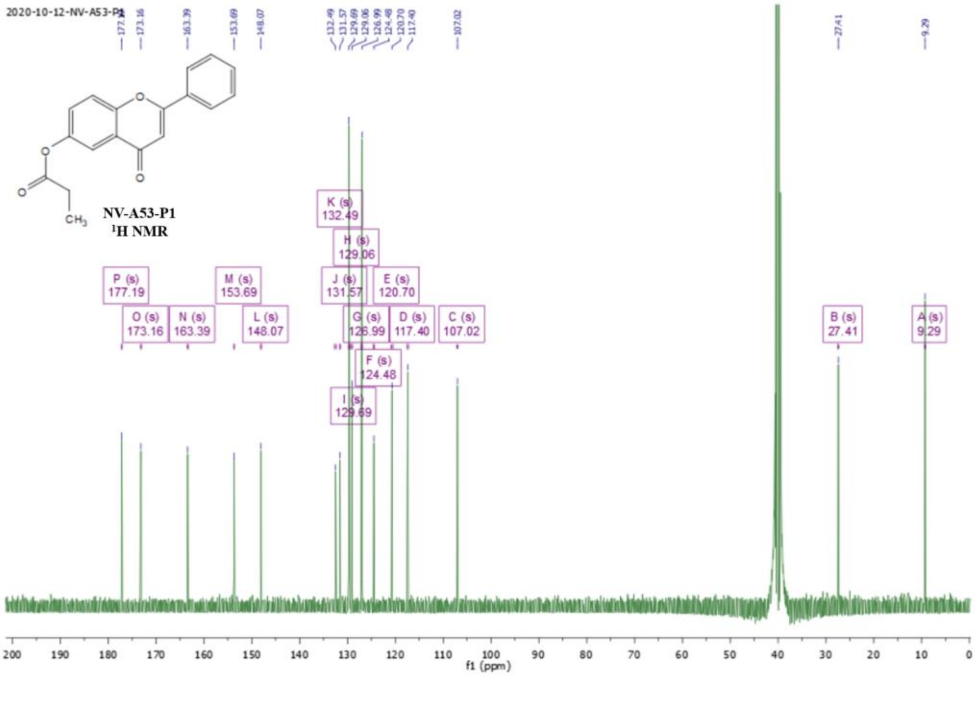

Figure S4 ^{13}C NMR spectrum of **1c**

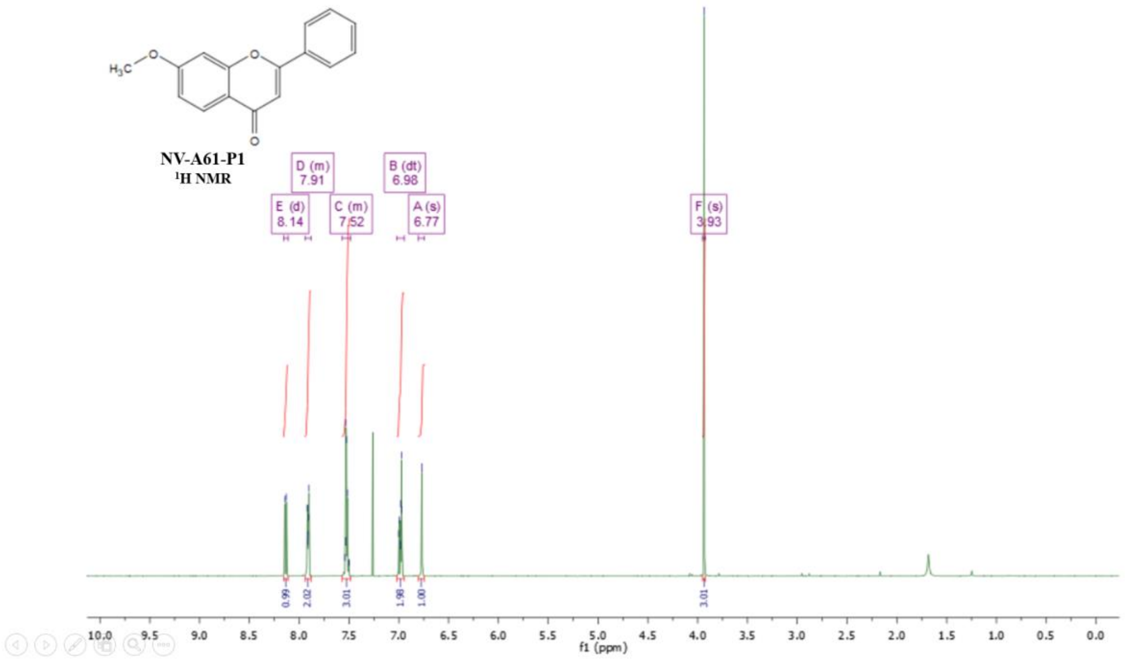

Figure S5 ^{13}C NMR spectrum of **2b**

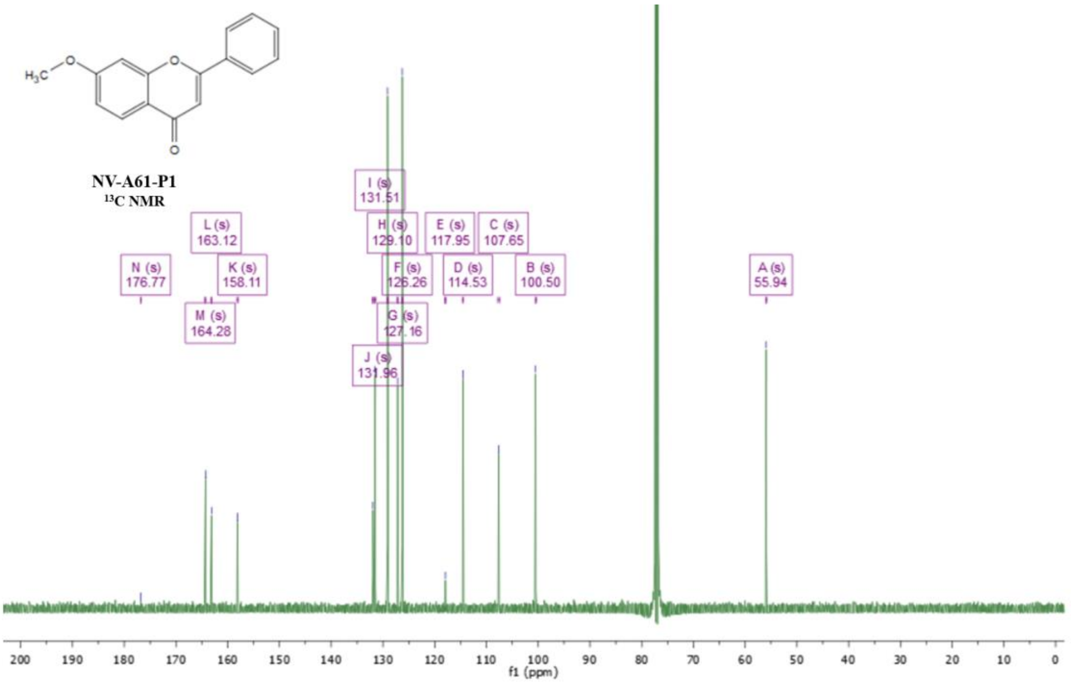

Figure S6 ^{13}C NMR spectrum of **2b**

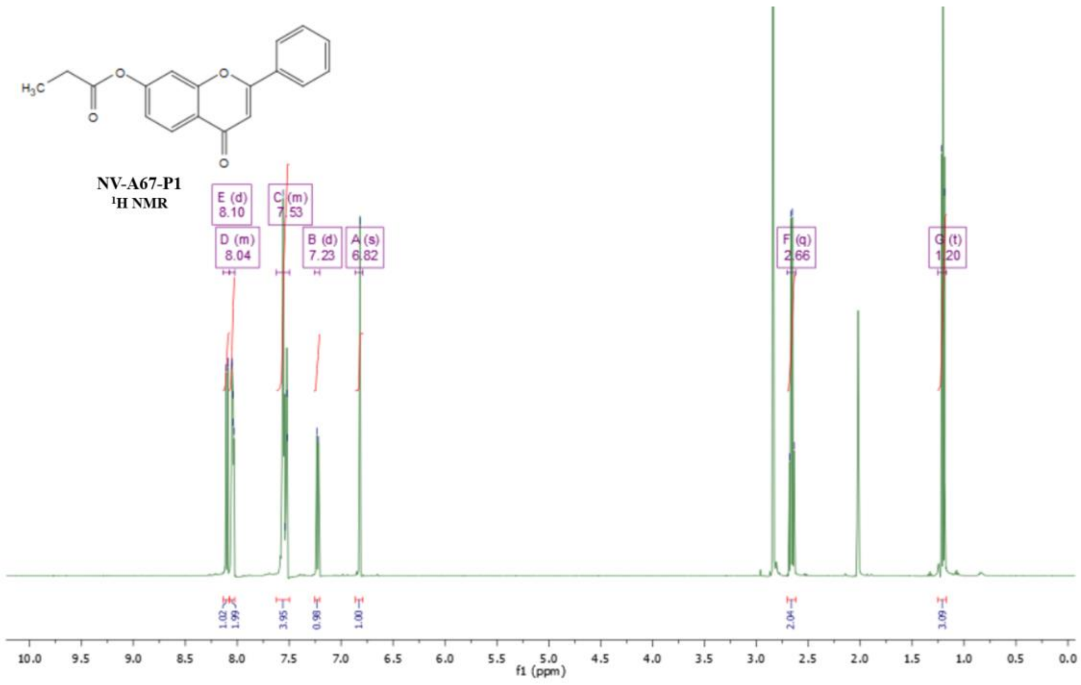

Figure S7 ^{13}C NMR spectrum of **2c**

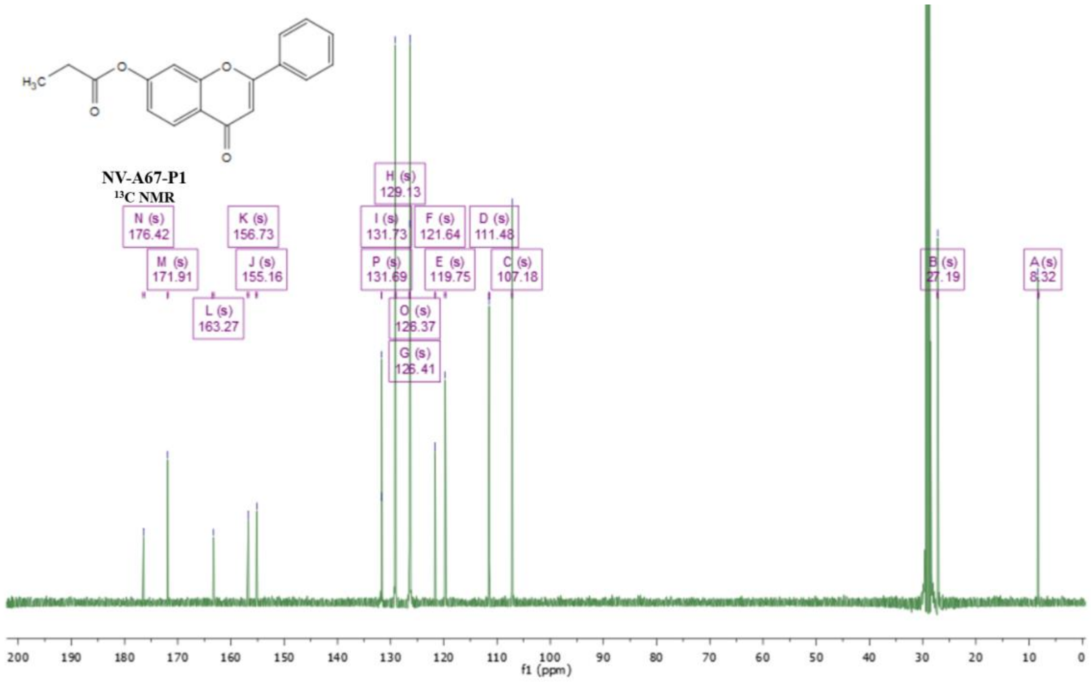

Figure S8 ^{13}C NMR spectrum of **2c**

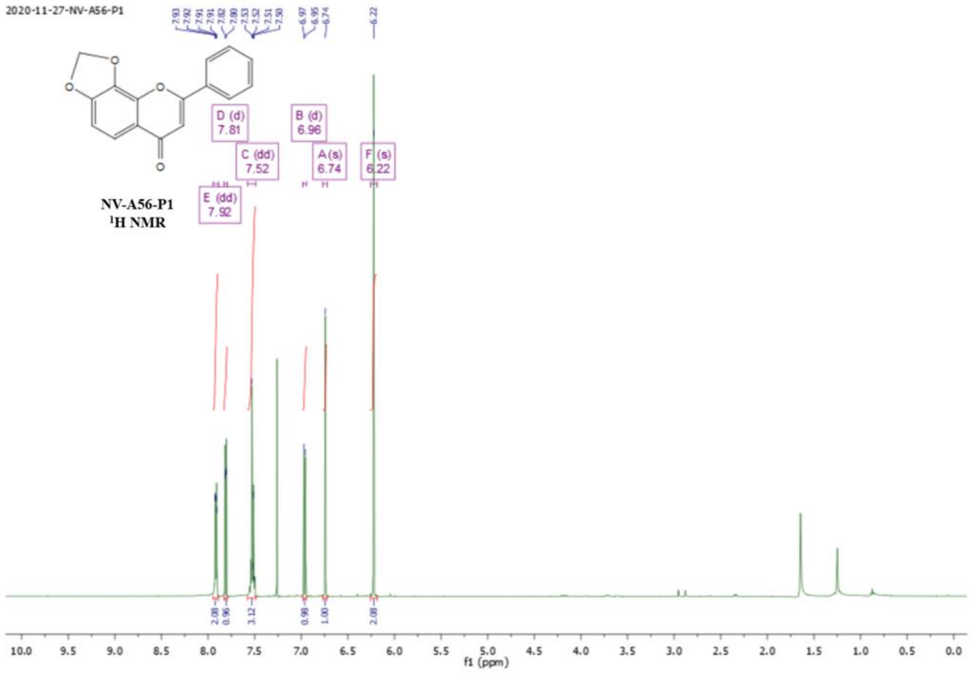

Figure S9 ^{1}H NMR spectrum of **3b**

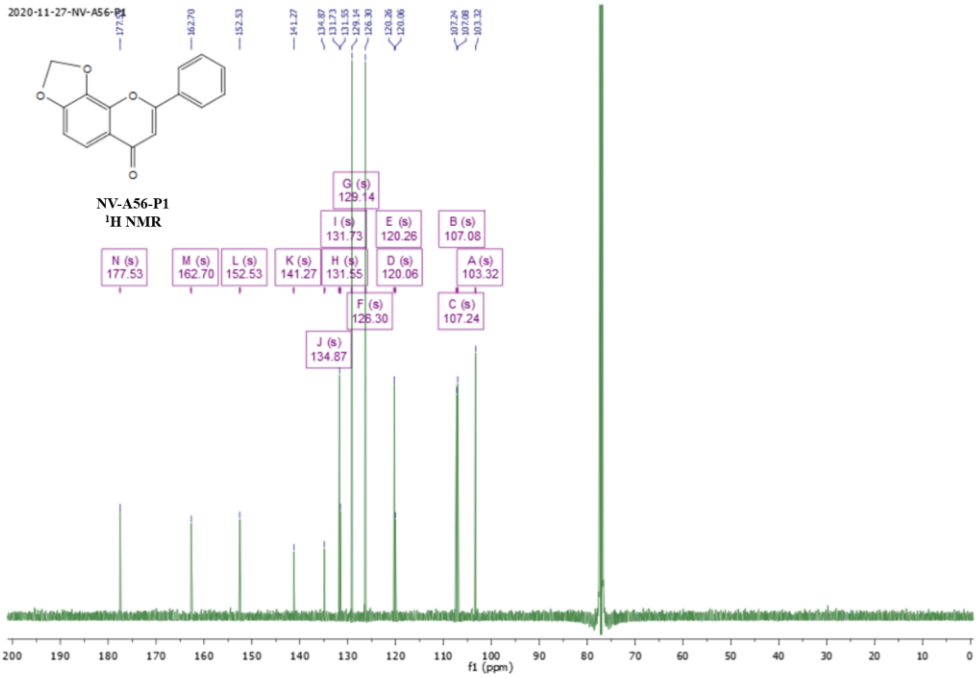

Figure S10 ^{13}C NMR spectrum of **3b**

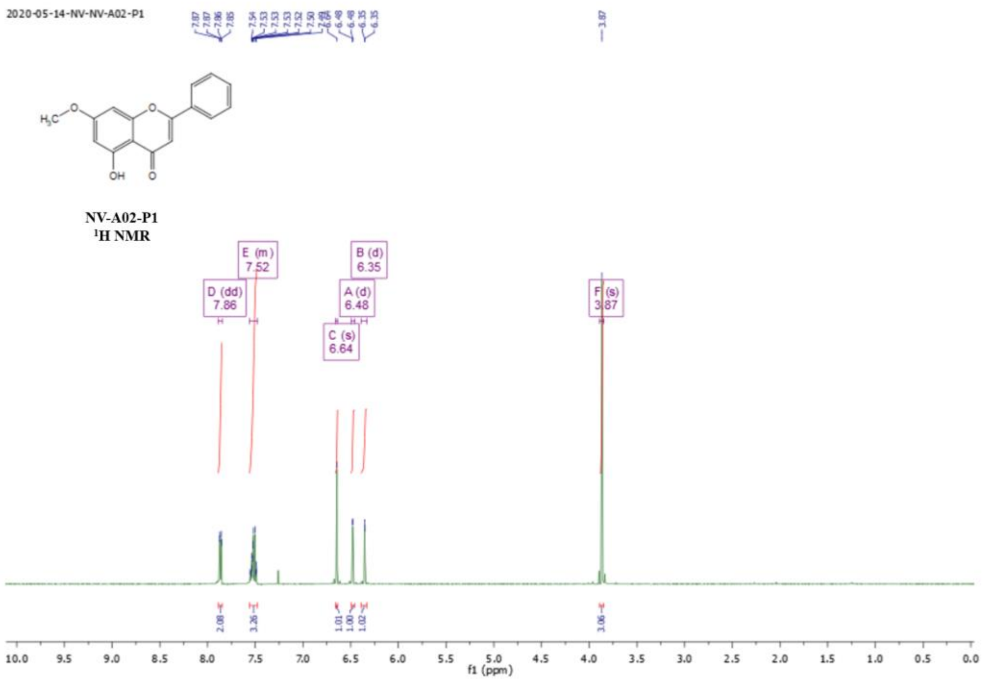

Figure S11 ^{1}H NMR spectrum of **4b**

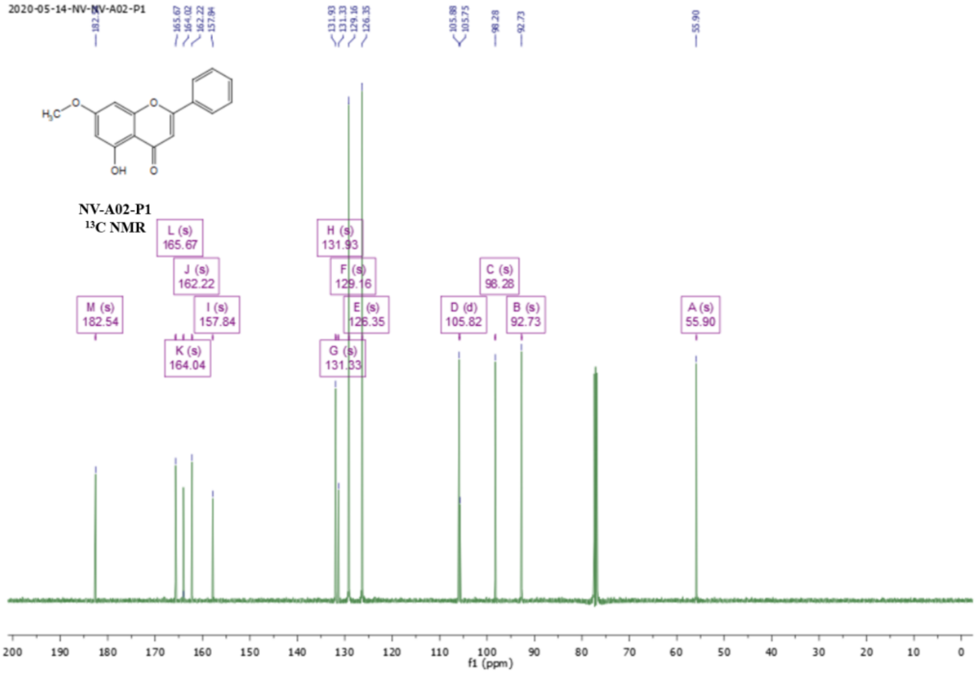

Figure S12 ^{13}C NMR spectrum of **4b**

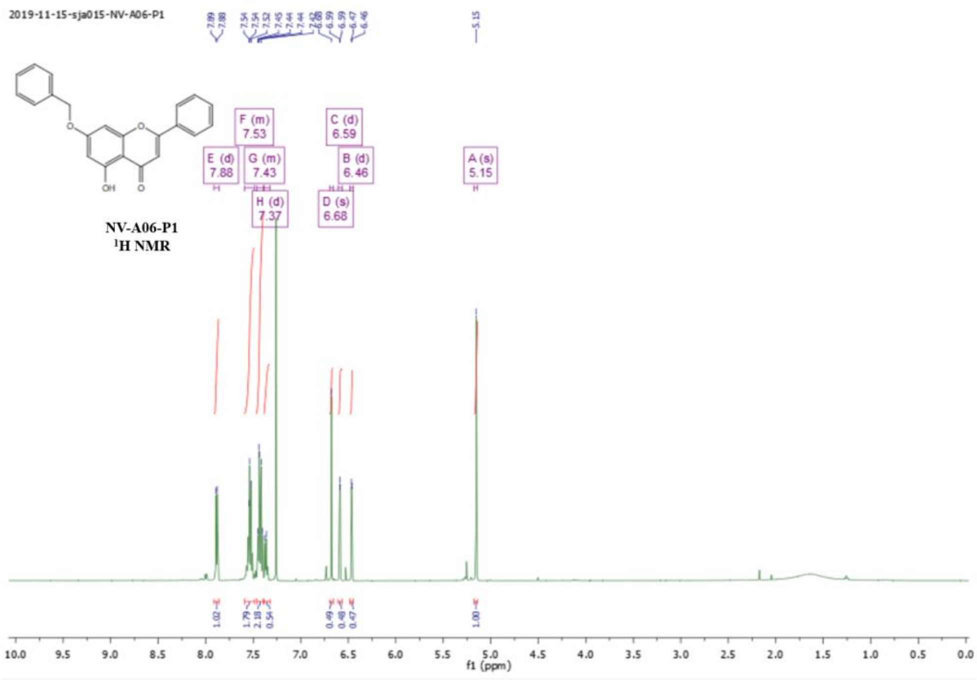

Figure S13 ^{1}H NMR spectrum of **4c**

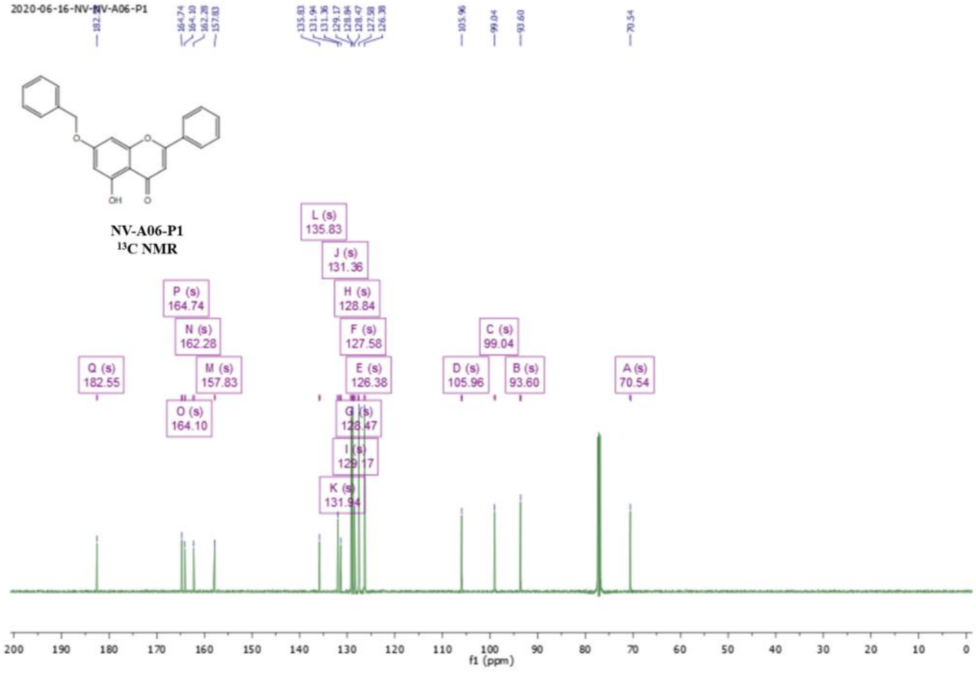

Figure S14 ^{13}C NMR spectrum of **4c**

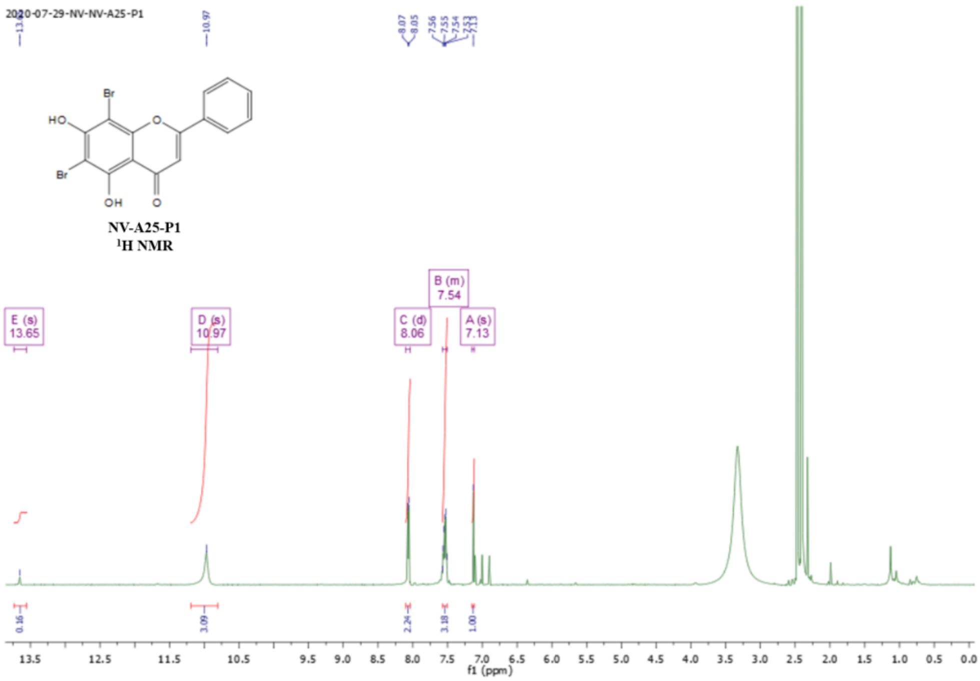

Figure S15 ^{1}H NMR spectrum of **4d**

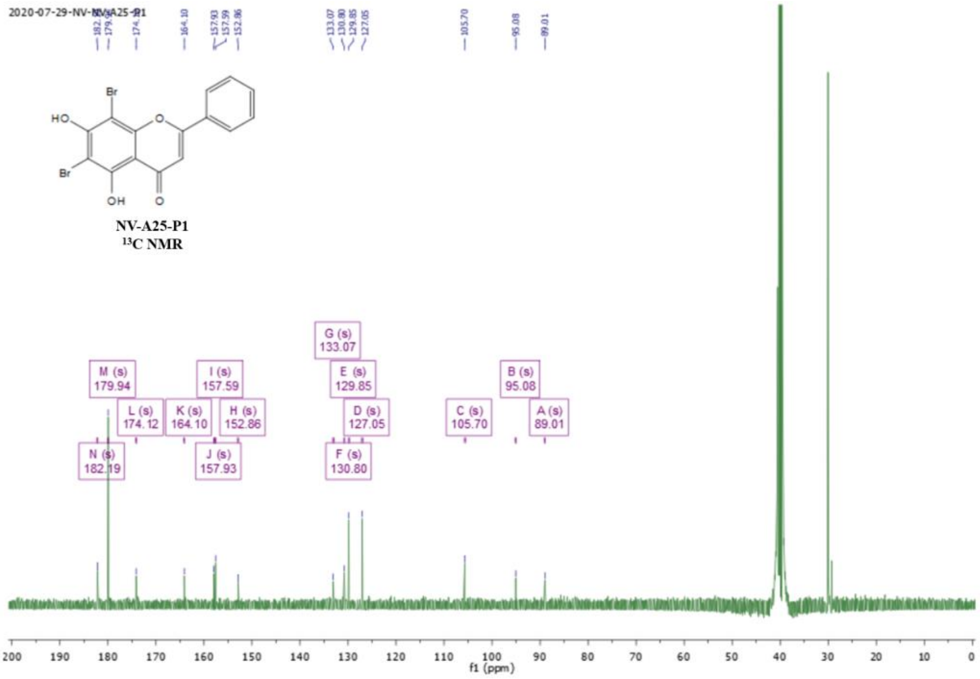

Figure S16 ^{13}C NMR spectrum of **4d**

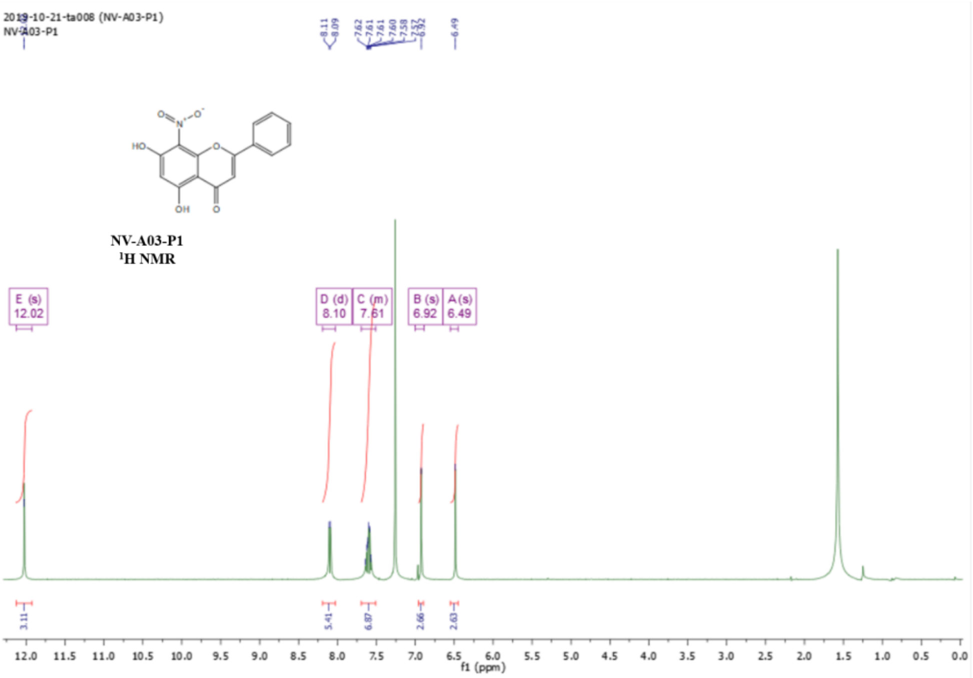

Figure S17 ^{1}H NMR spectrum of **4e**

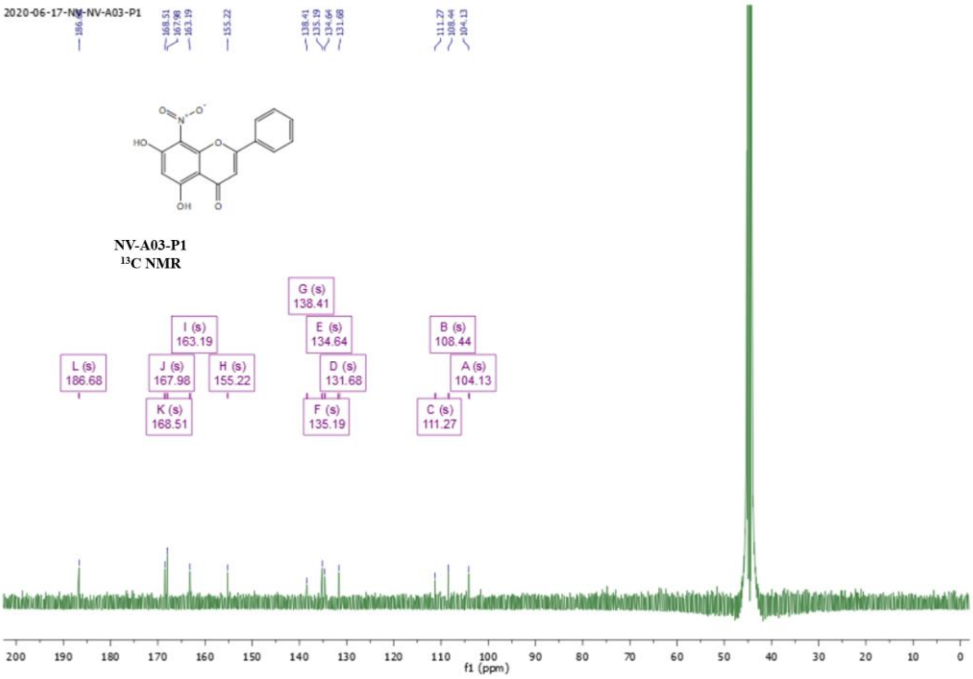

Figure S18 ^{13}C NMR spectrum of **4e**

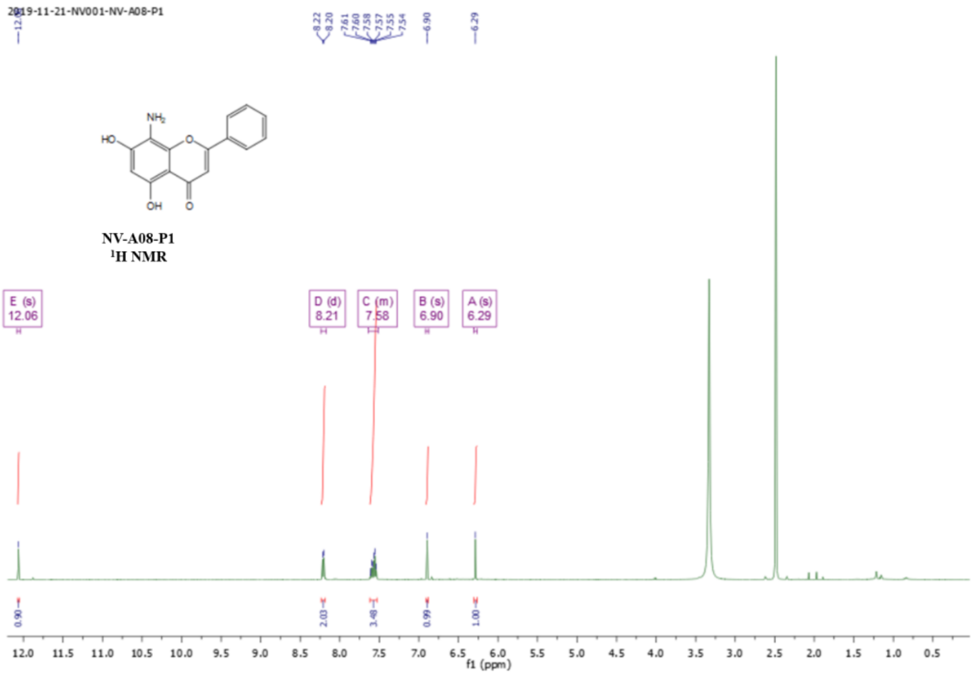

Figure S19 ^{1}H NMR spectrum of **4f**

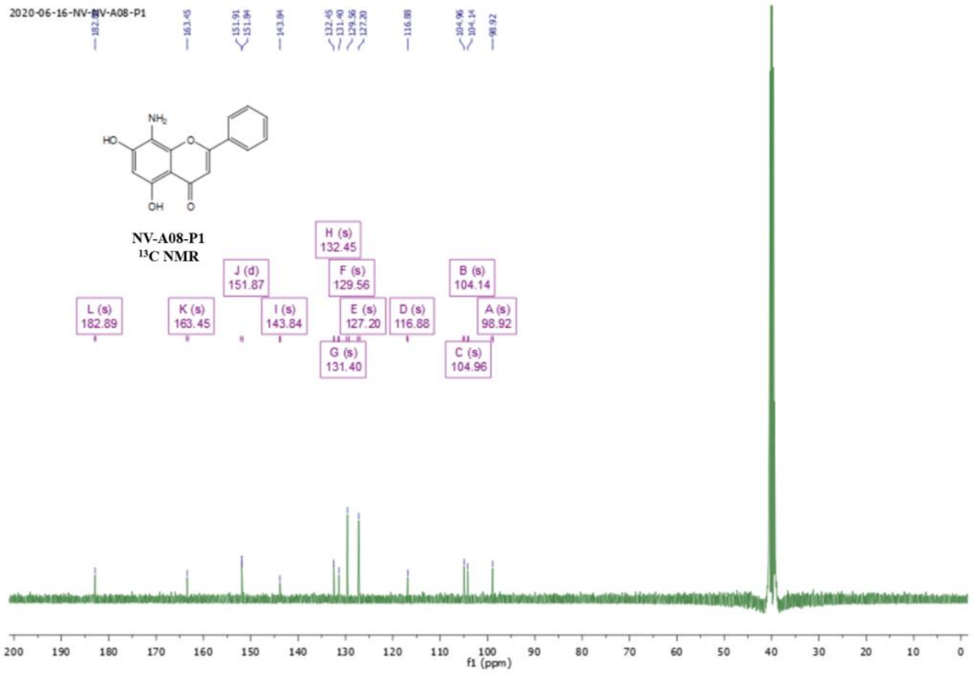

Figure S20 ^{13}C NMR spectrum of **4f**

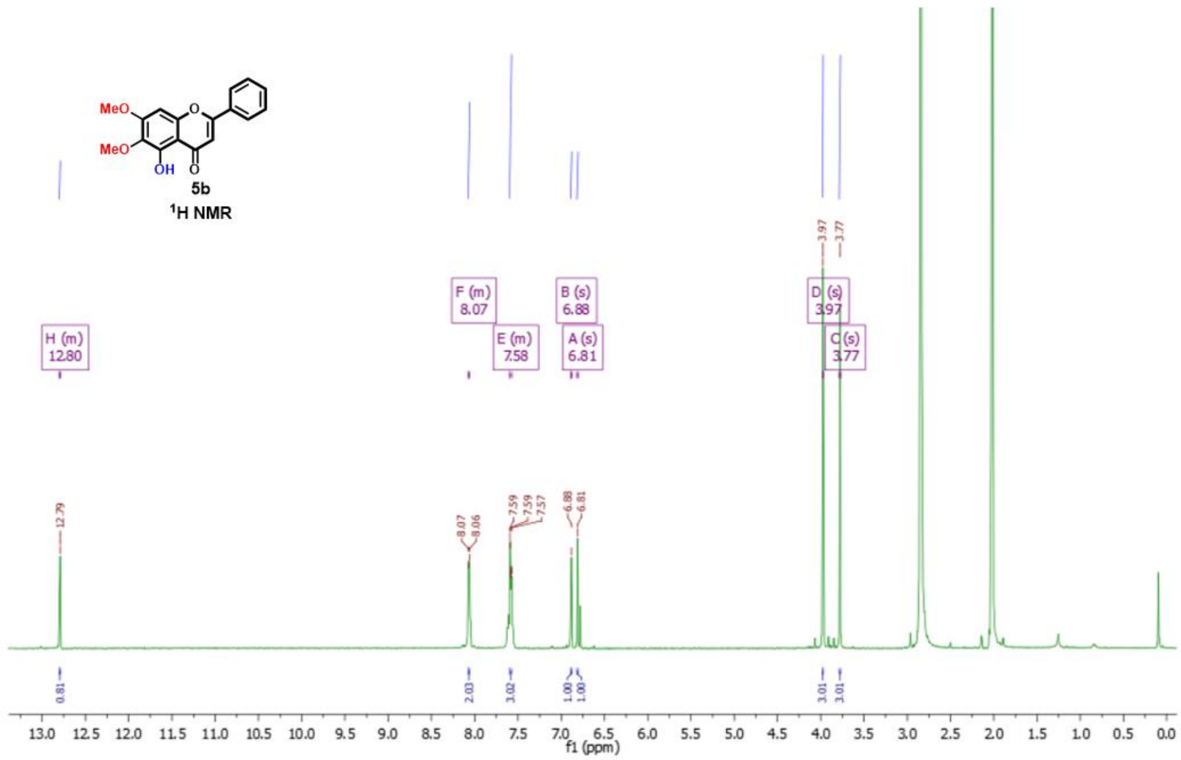

Figure S21 ^{1}H NMR spectrum of **5b**

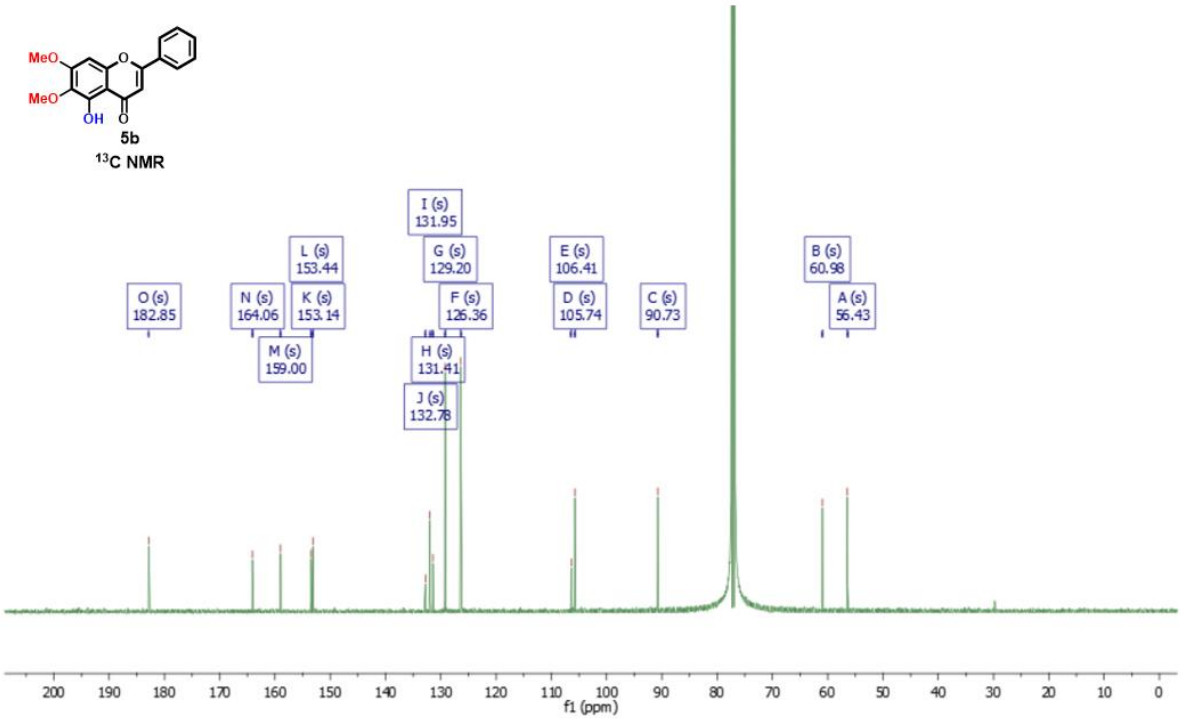

Figure S22 ^{13}C NMR spectrum of **5b**

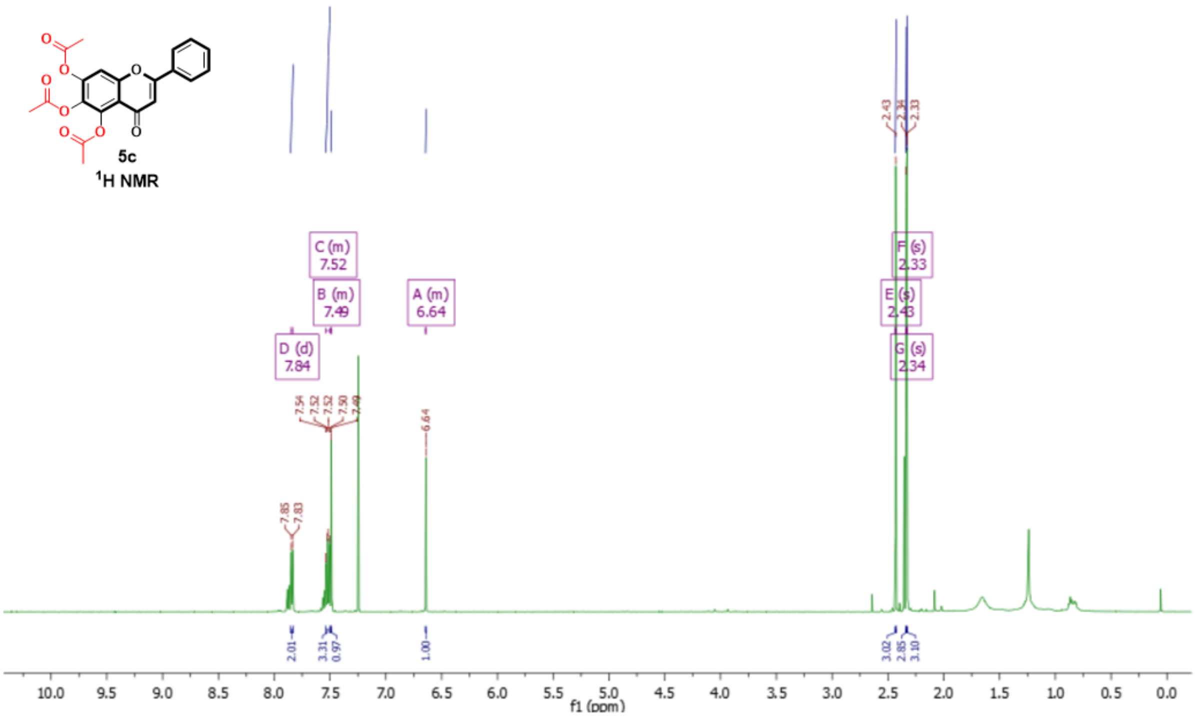

Figure S23 ^{1}H NMR spectrum of **5c**

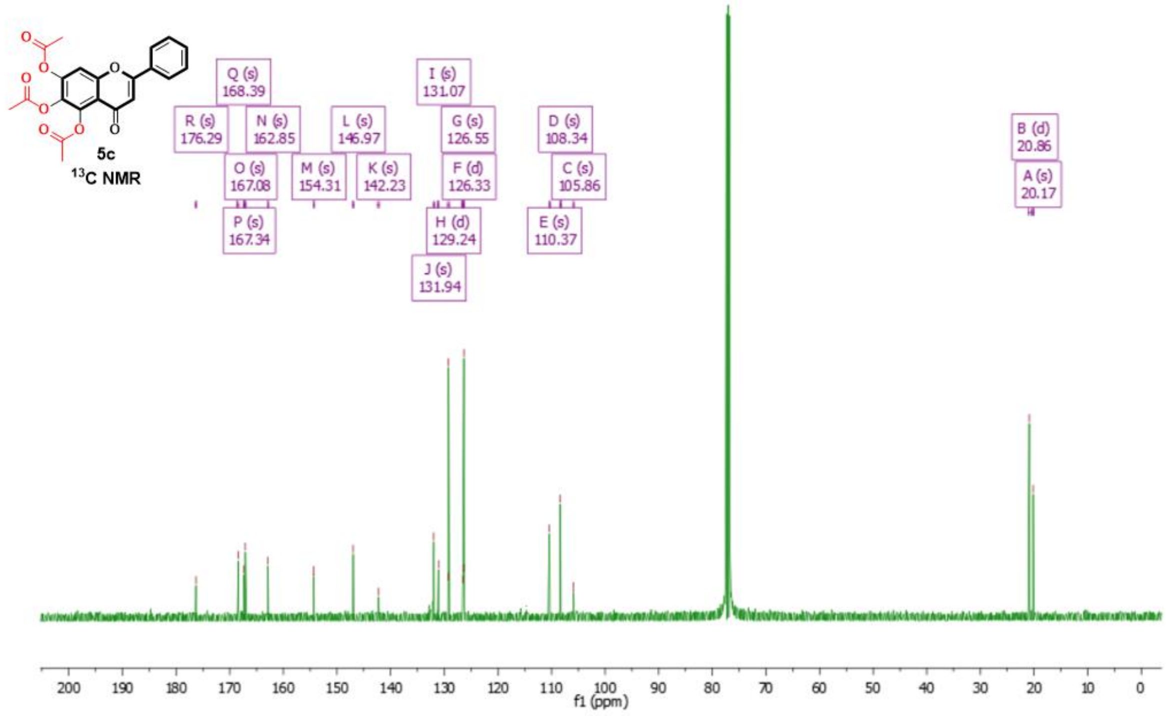

Figure S24 ^{13}C NMR spectrum of **5c**

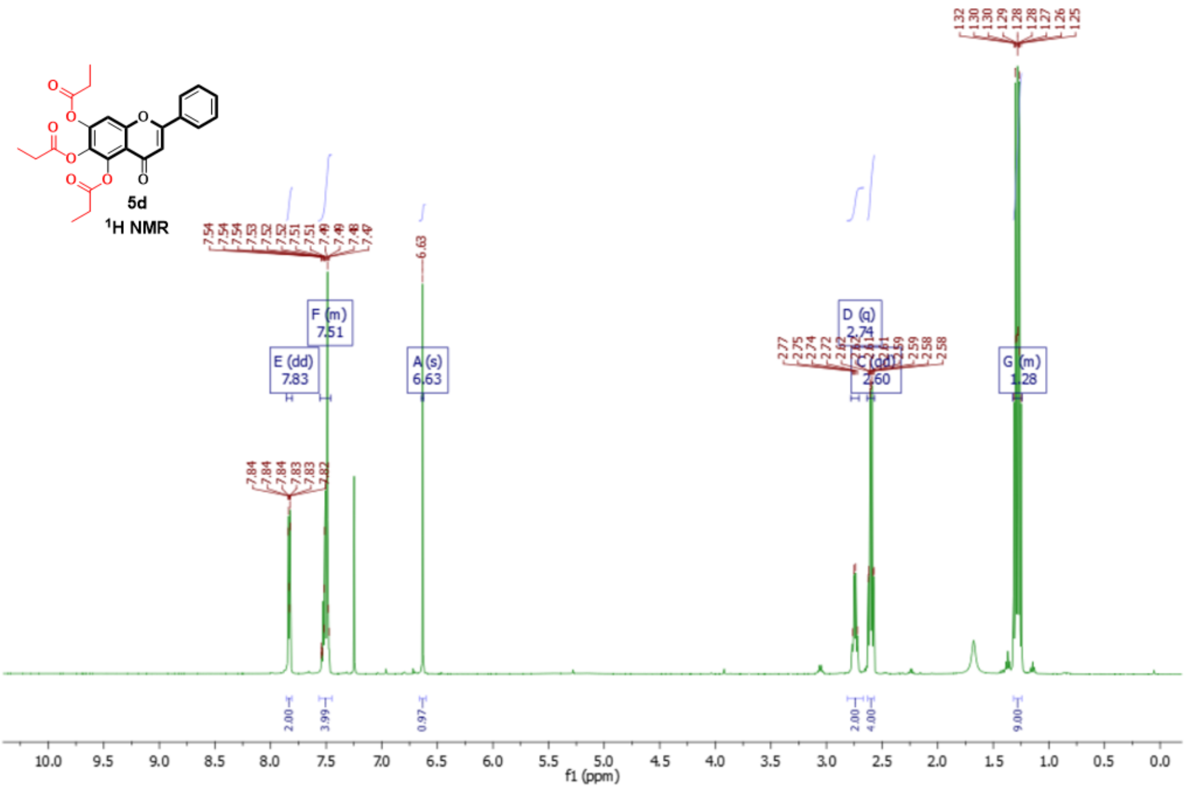

Figure S25 ^{1}H NMR spectrum of **5d**

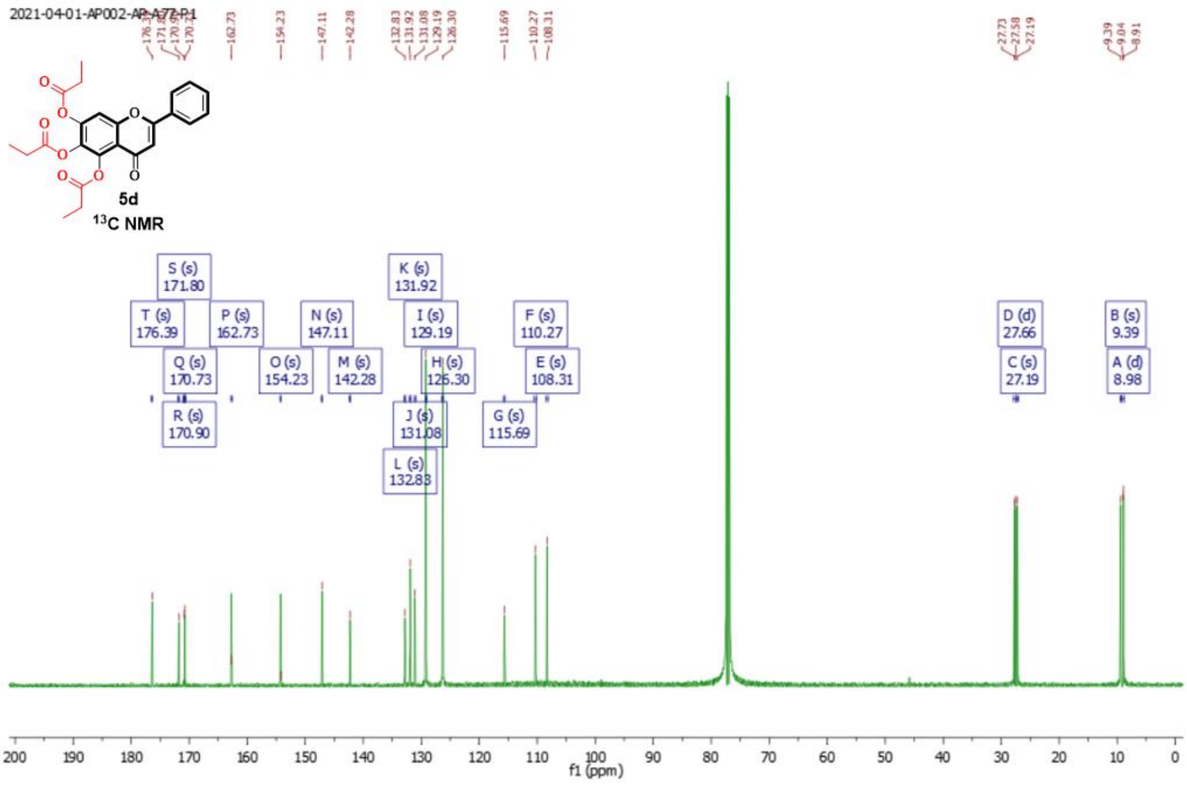

Figure S26 ^{13}C NMR spectrum of **5d**

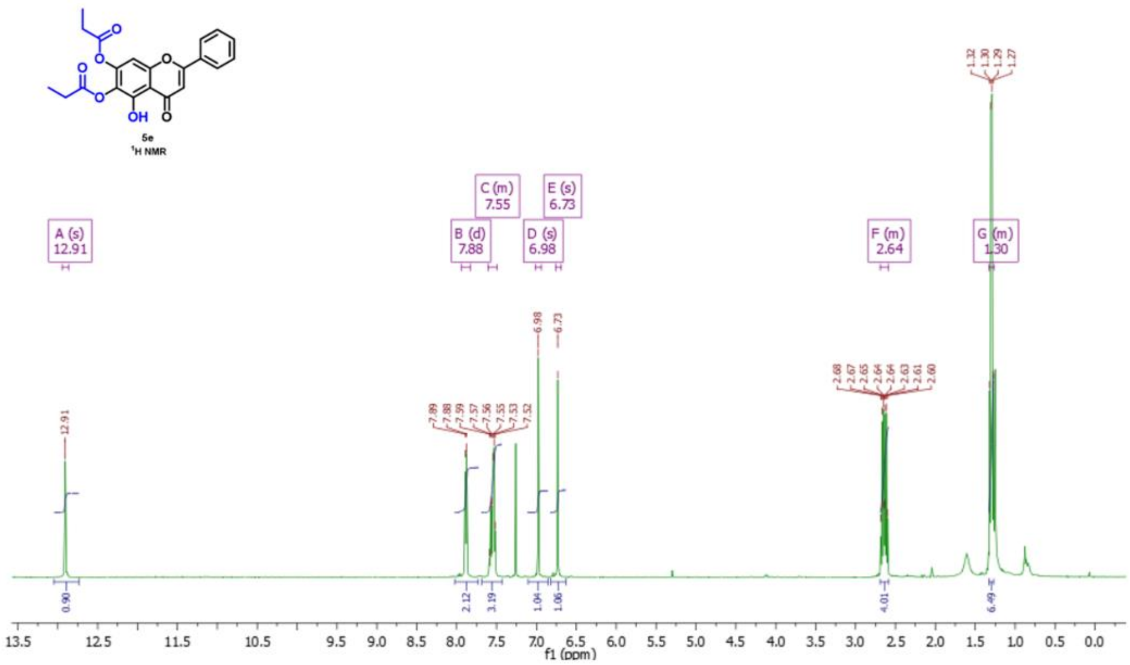

Figure S27 ^{1}H NMR spectrum of **5e**

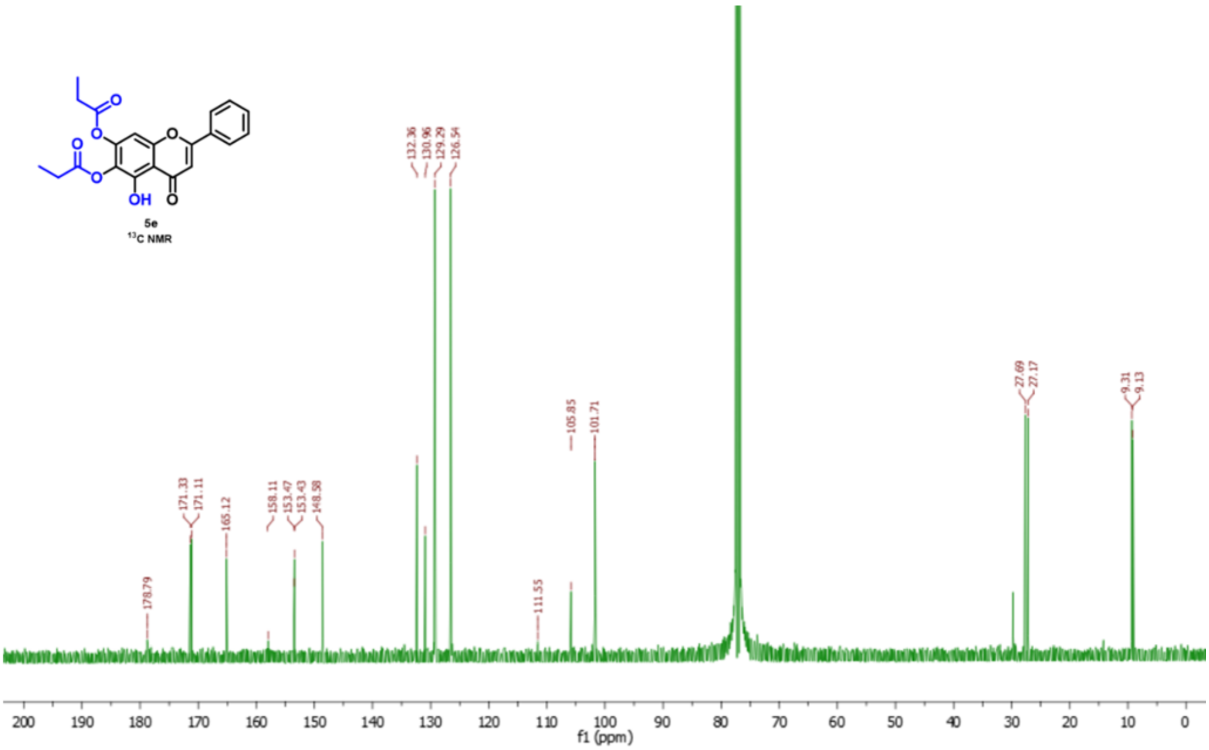

Figure S28 ^{13}C NMR spectrum of **5e**

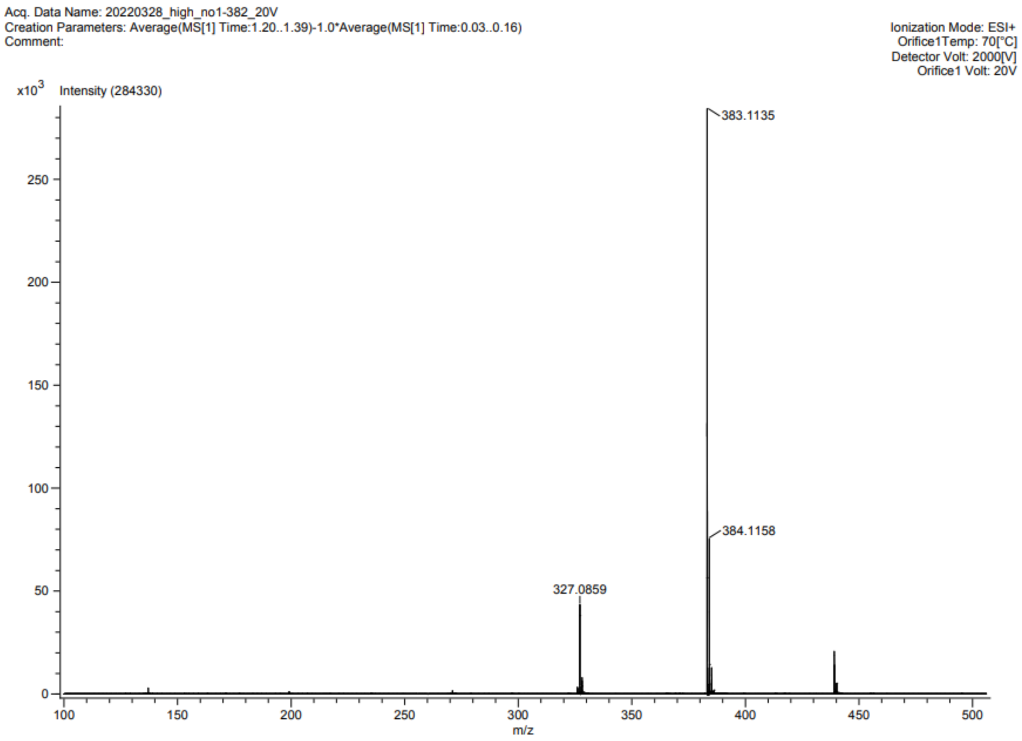

Figure S29 HSMS of **5e**

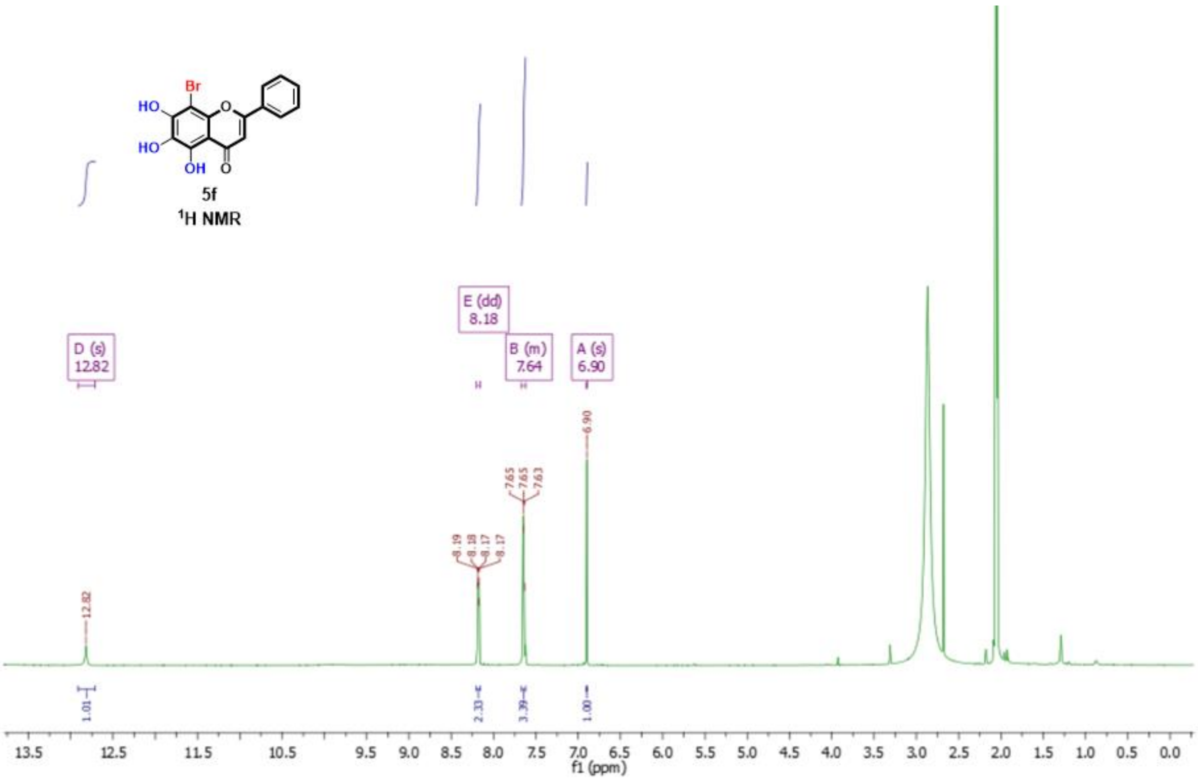

Figure S30 ^{1}H NMR spectrum of **5f**

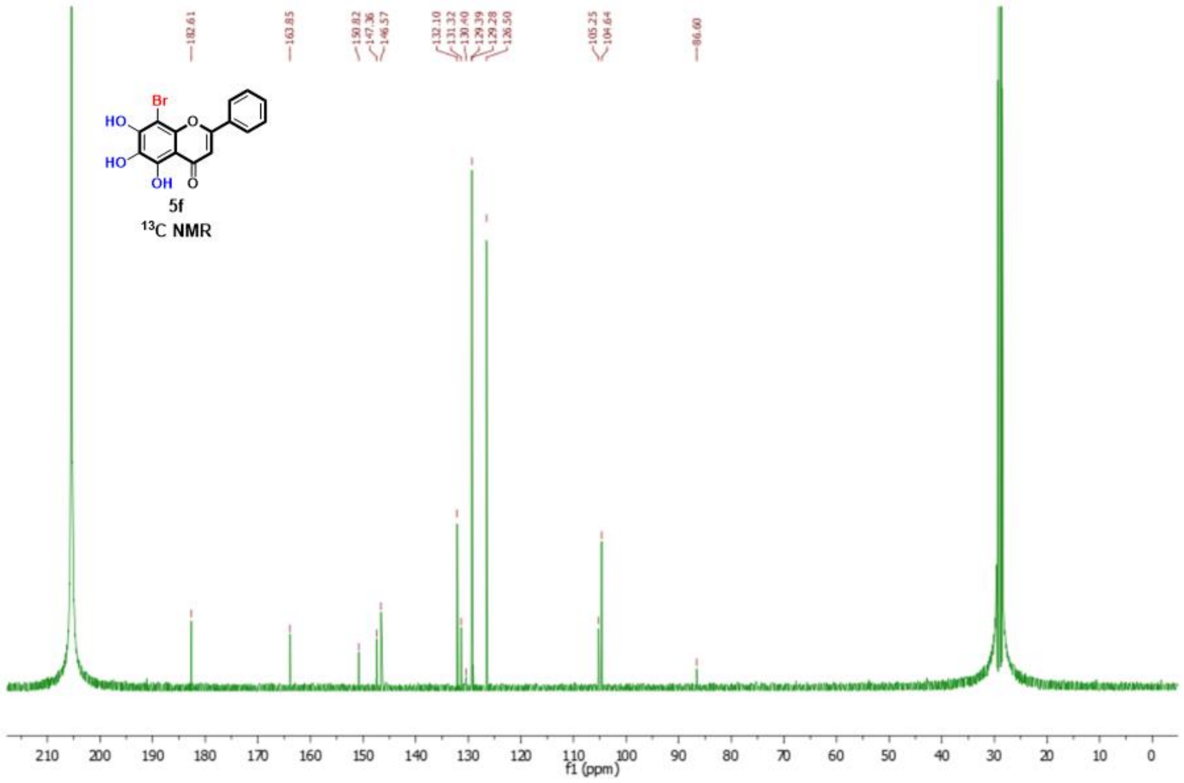

Figure S31 ^{13}C NMR spectrum of **5f**

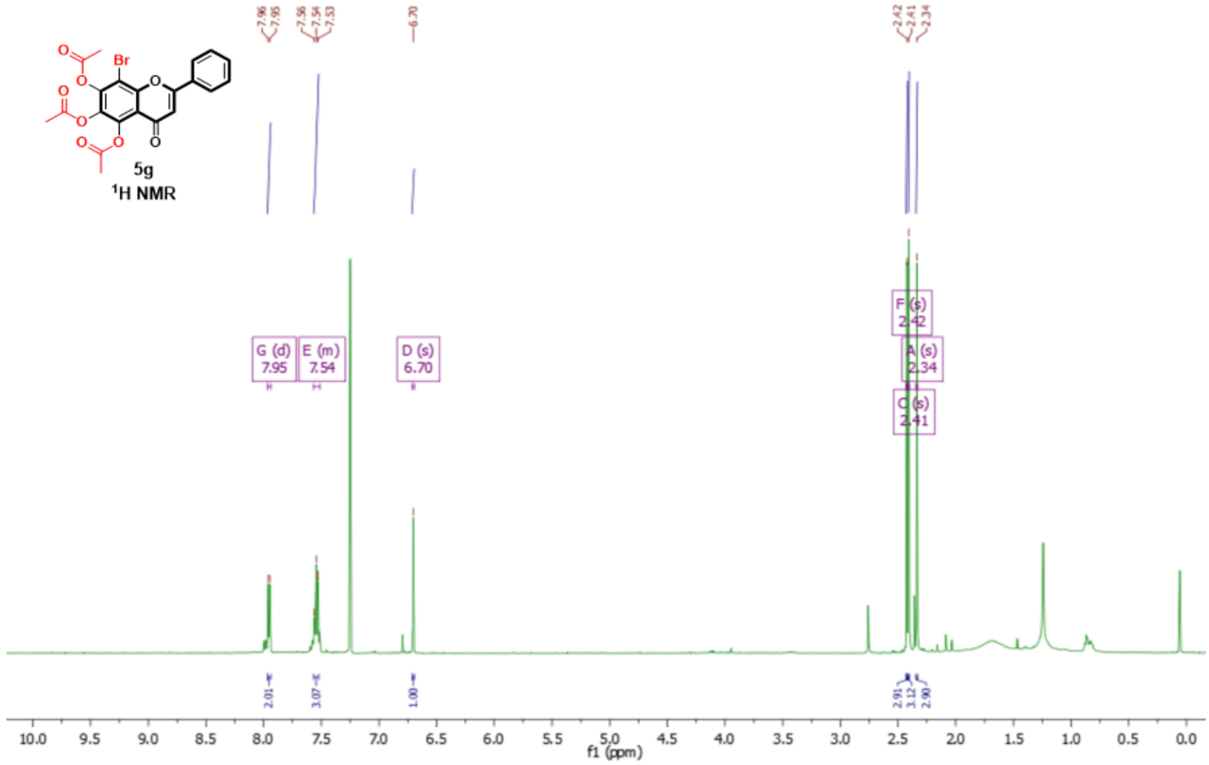

Figure S32 ^{1}H NMR spectrum of **5g**

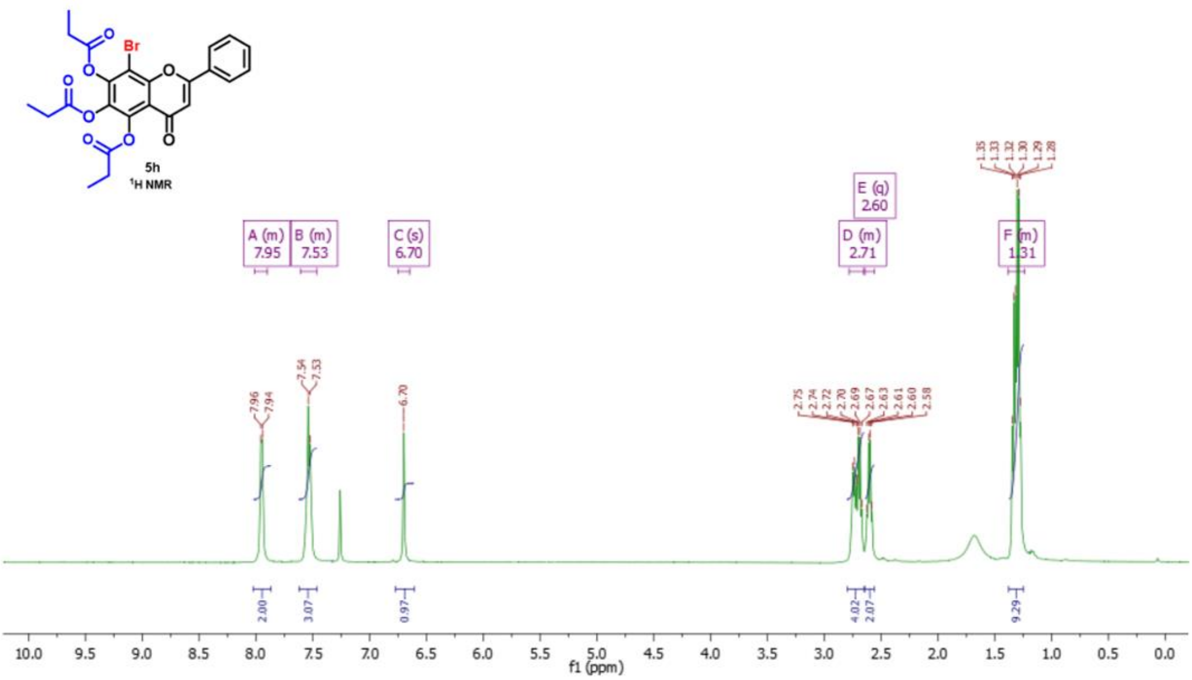

Figure S33 ^{1}H NMR of **5h**

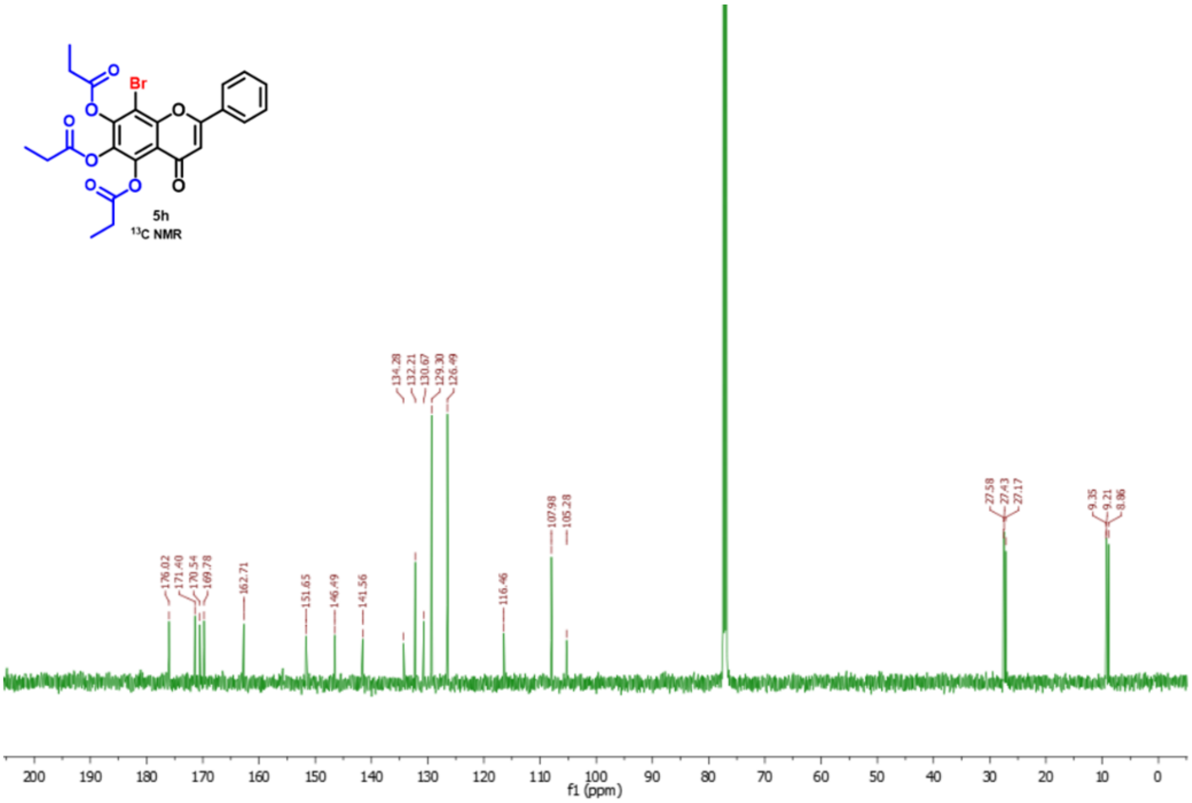

Figure S34 ^{13}C NMR of **5h**

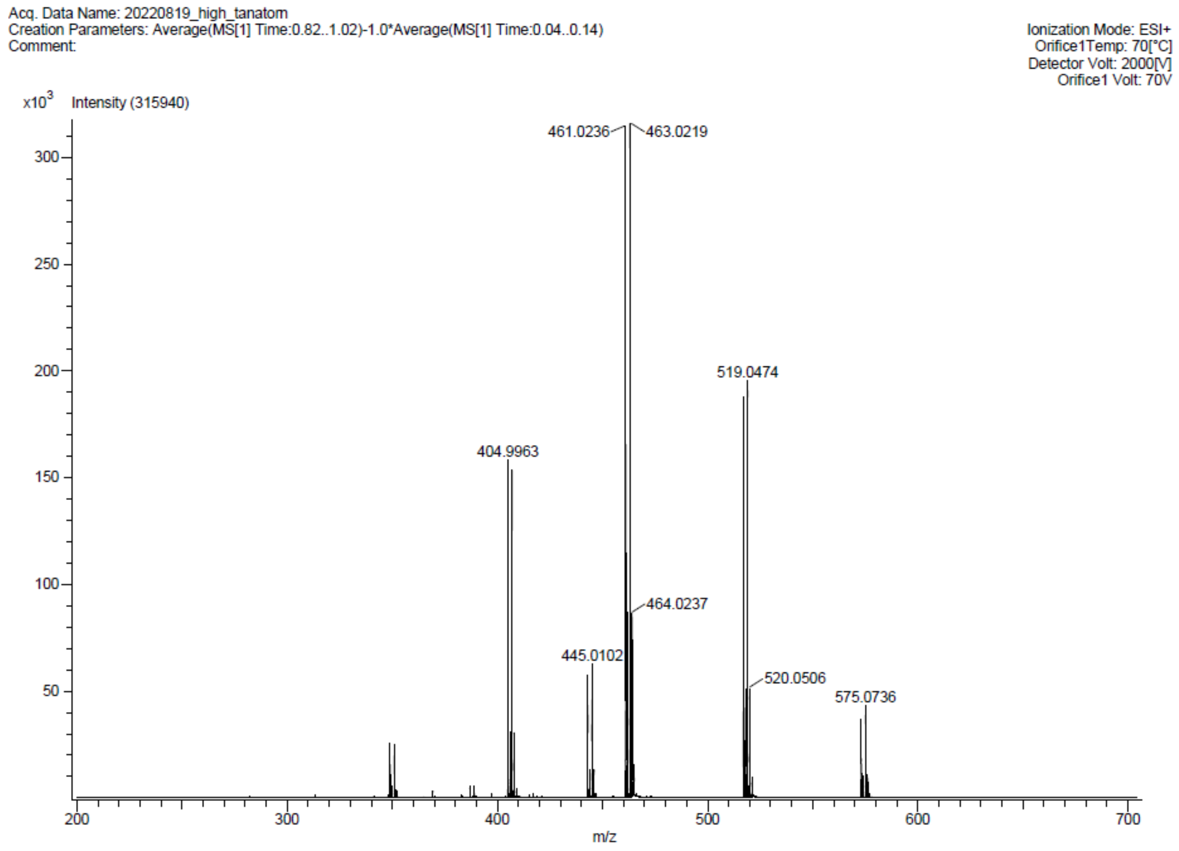

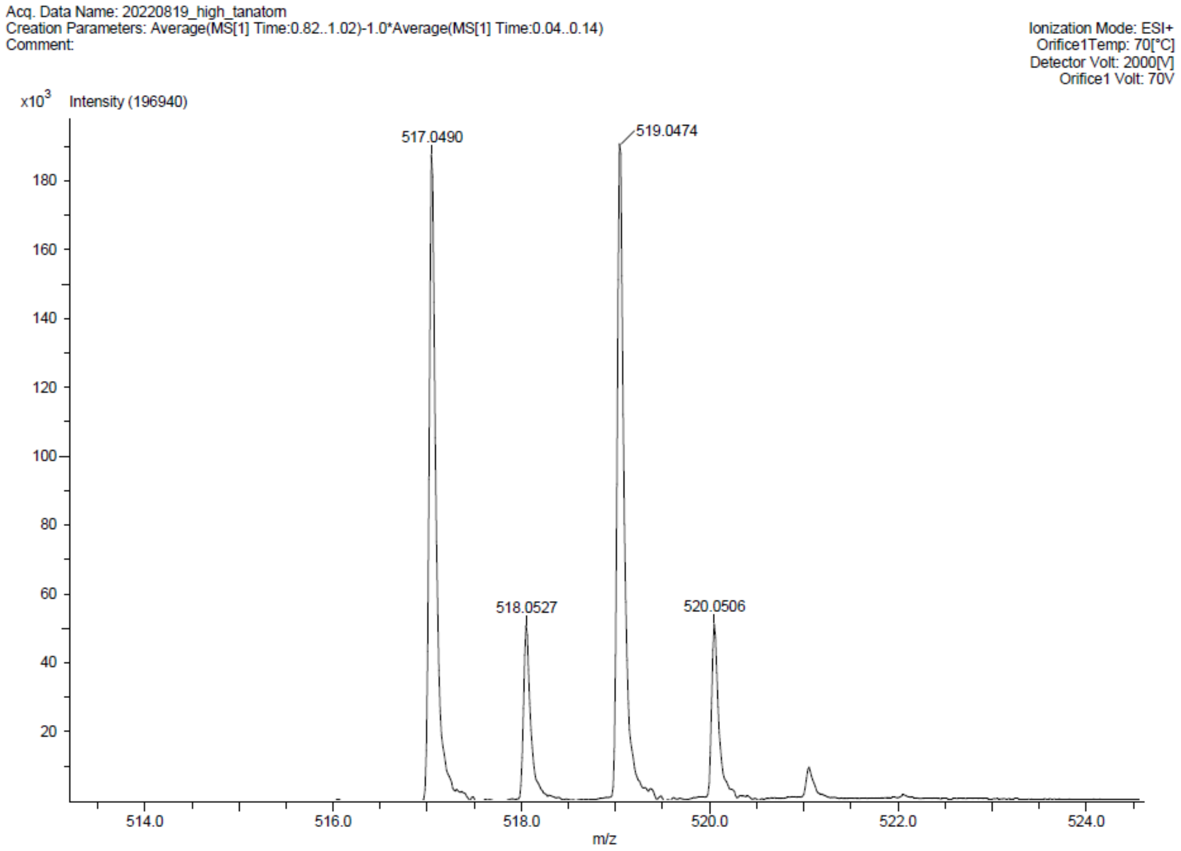

Figure S35 HSMS of **5h**

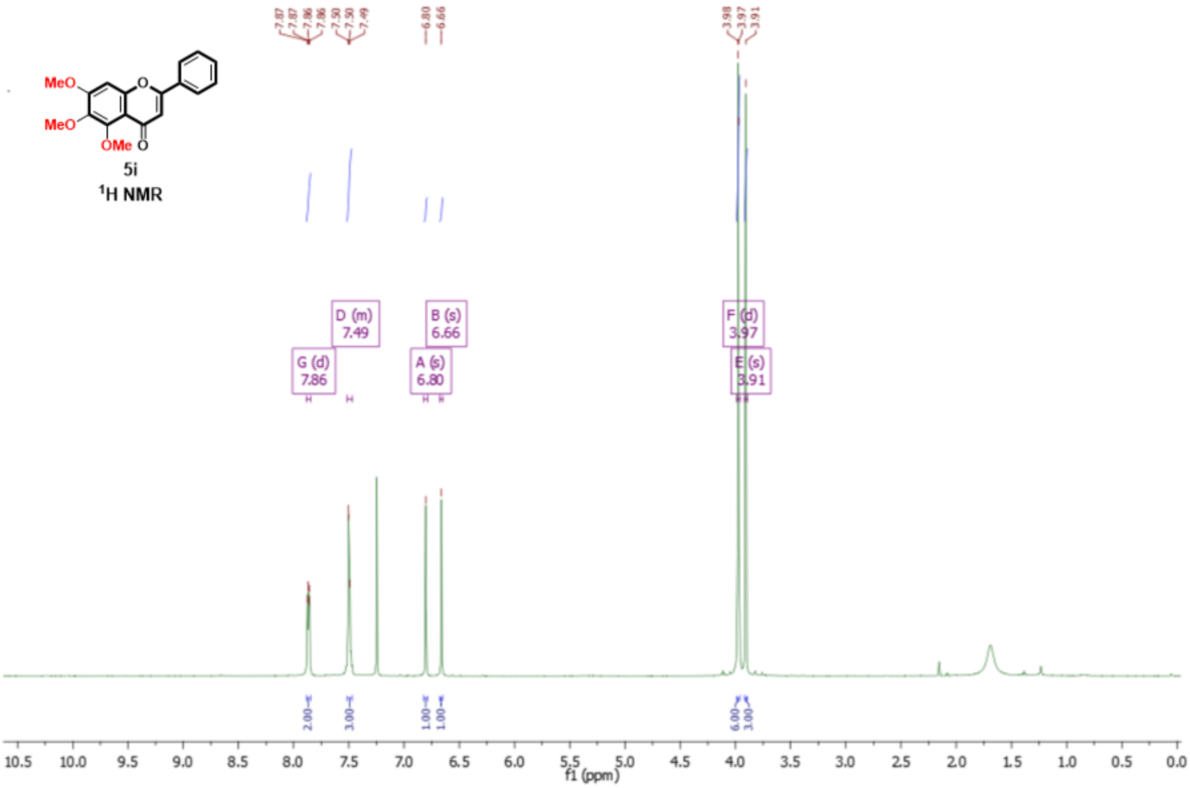

Figure S36 ^{1}H NMR of **5i**

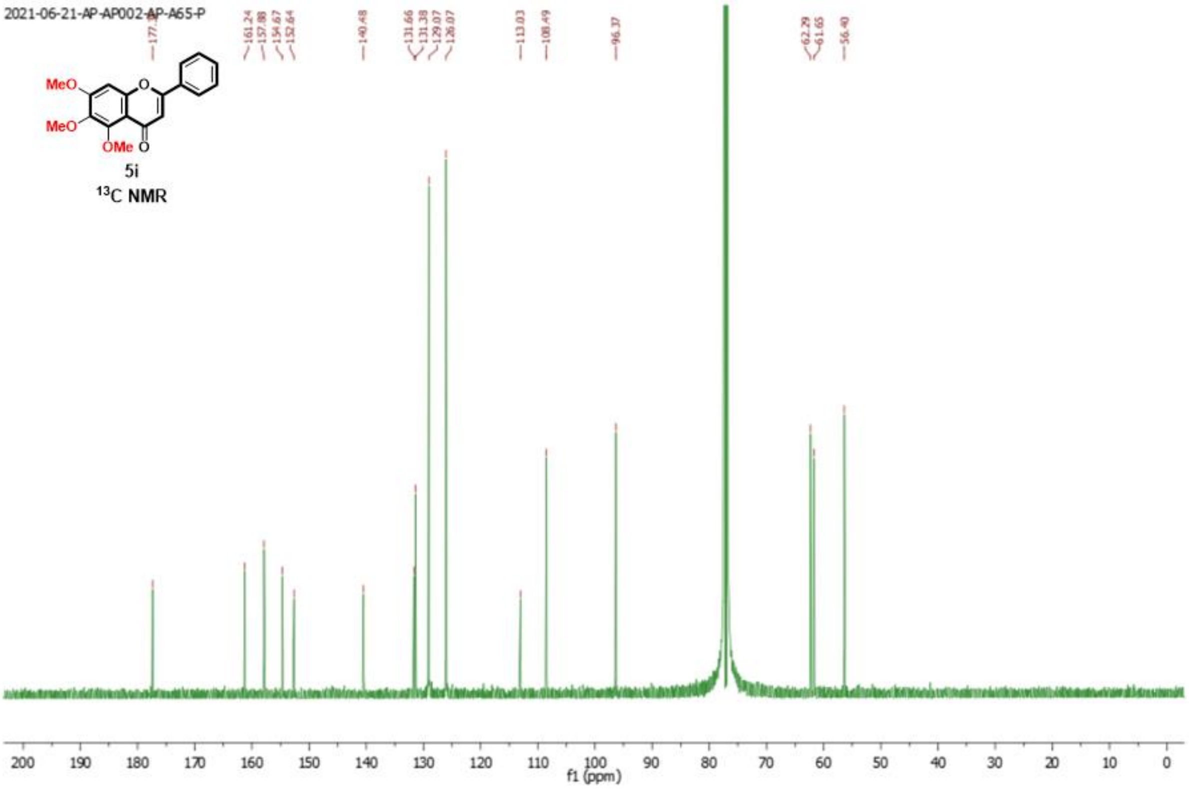

Figure S37 ^{13}C NMR of **5i**

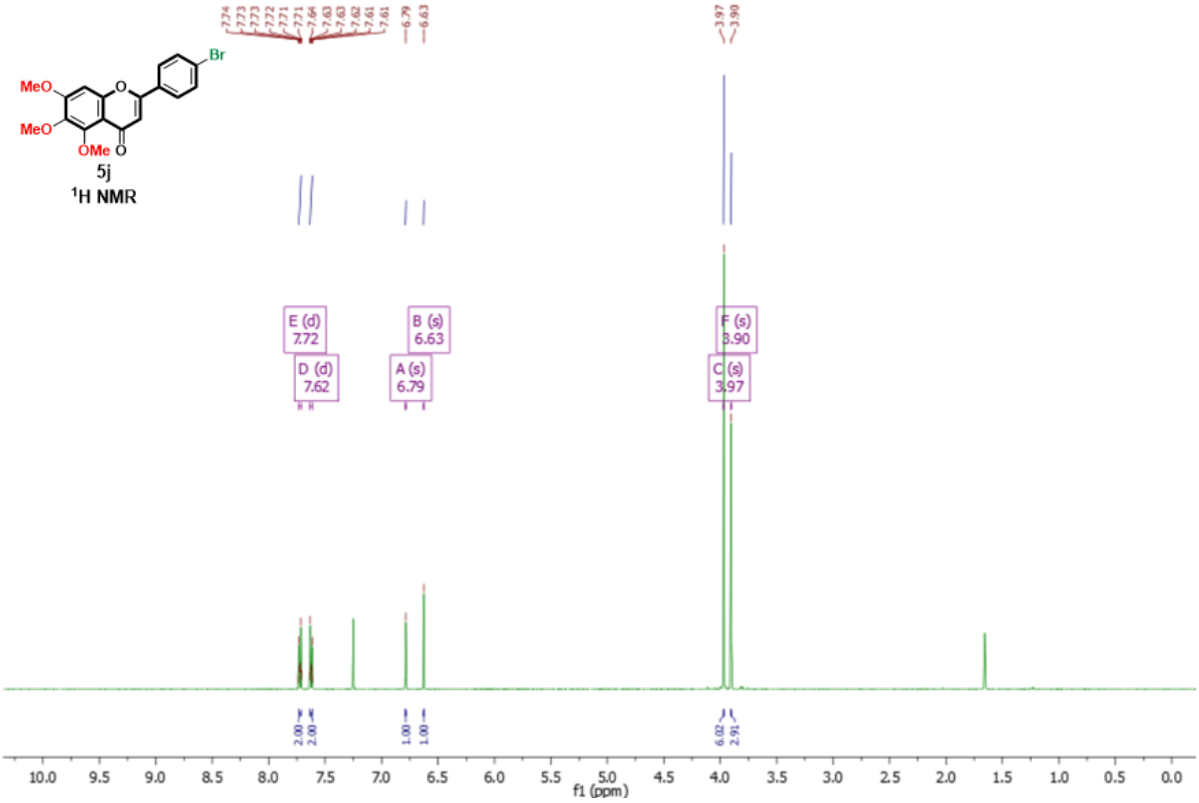

Figure S38 ^{1}H NMR of **5j**

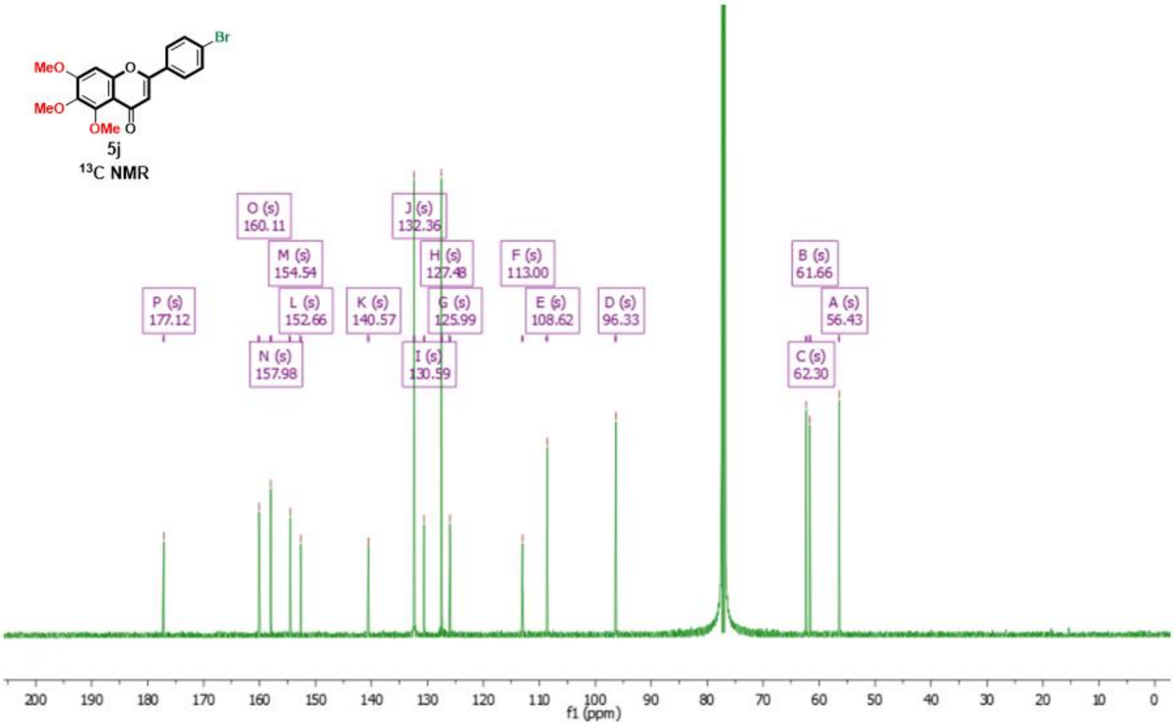

Figure S39 ^{13}C NMR of **5j**

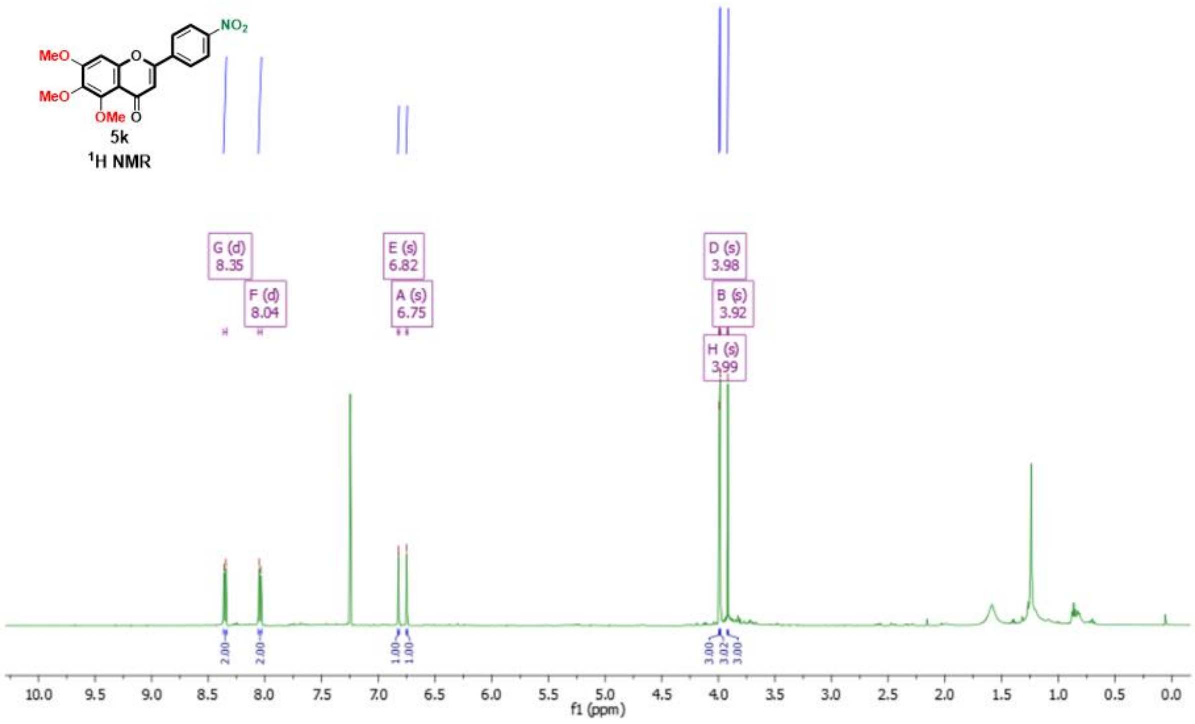

Figure S40 ^{1}H NMR of **5k**

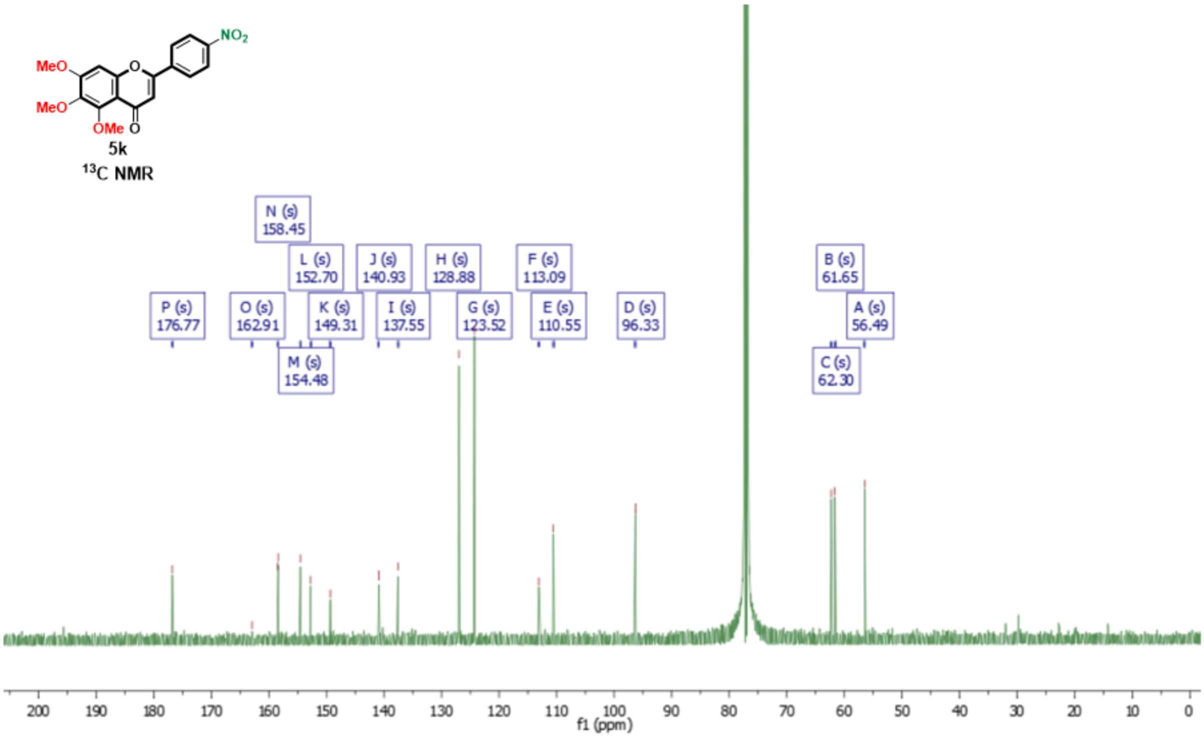

Figure S41 ^{13}C NMR of **5k**

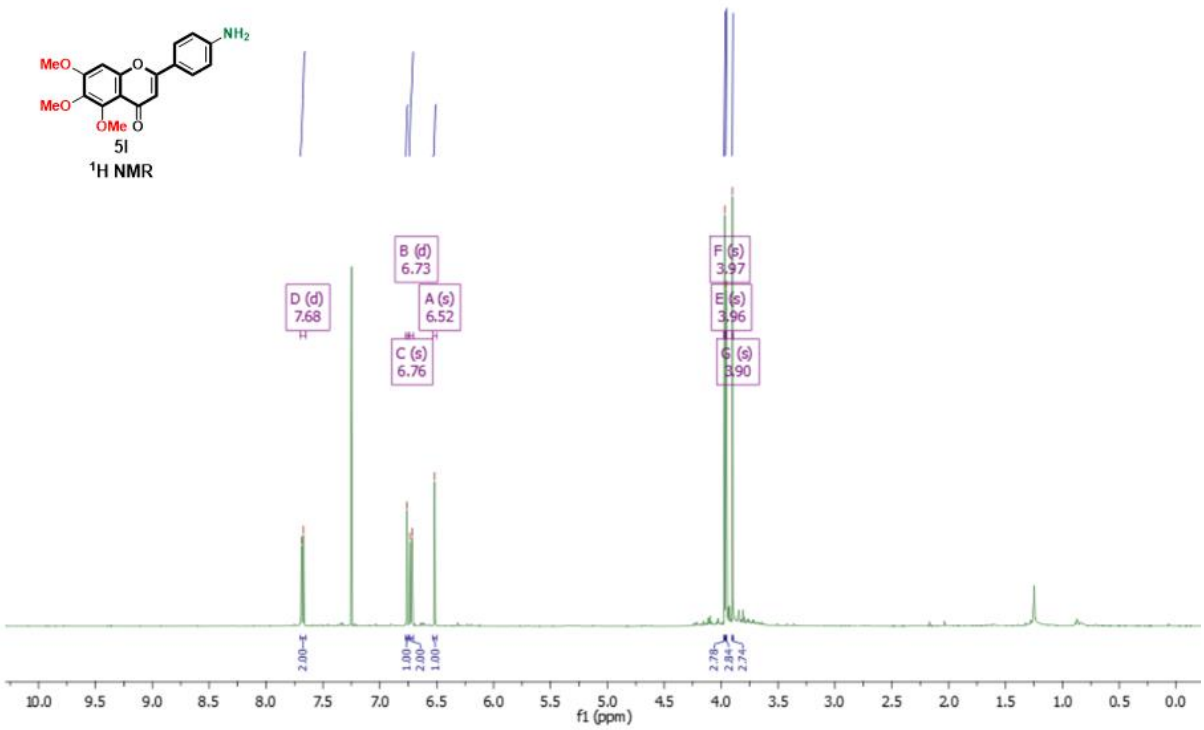

Figure S42 ^{1}H NMR of **5l**

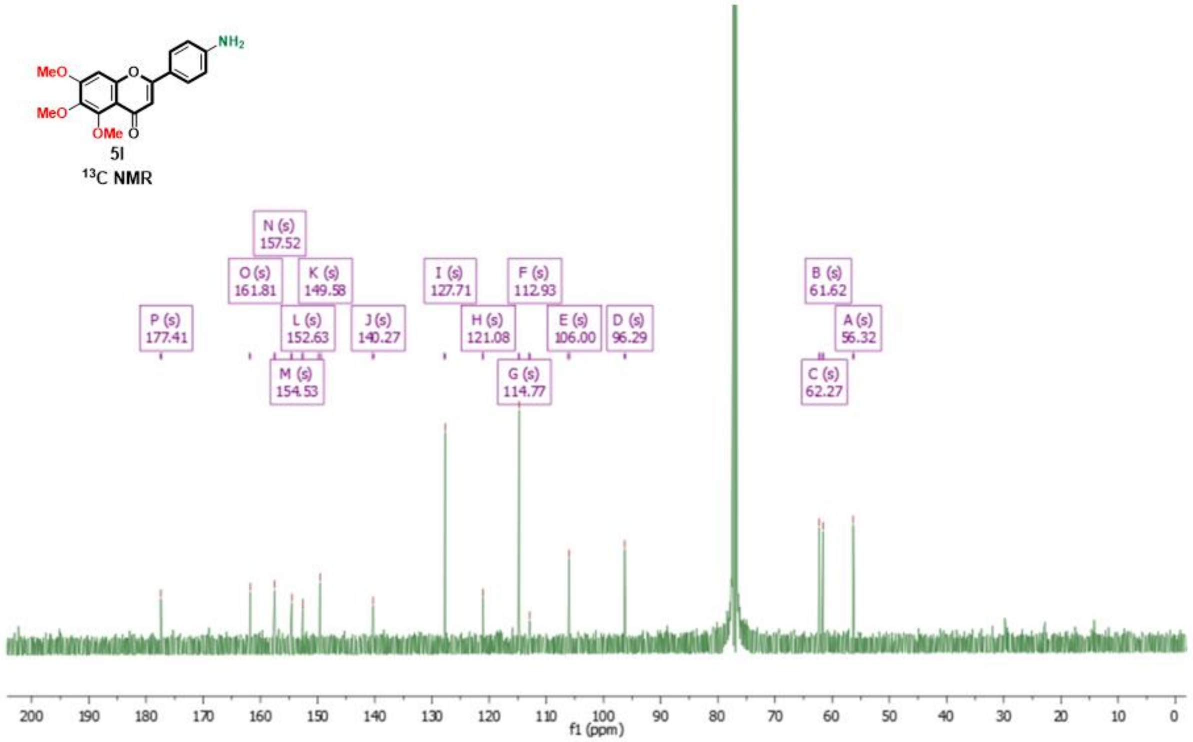

Figure S43 ^{13}C NMR of **5l**

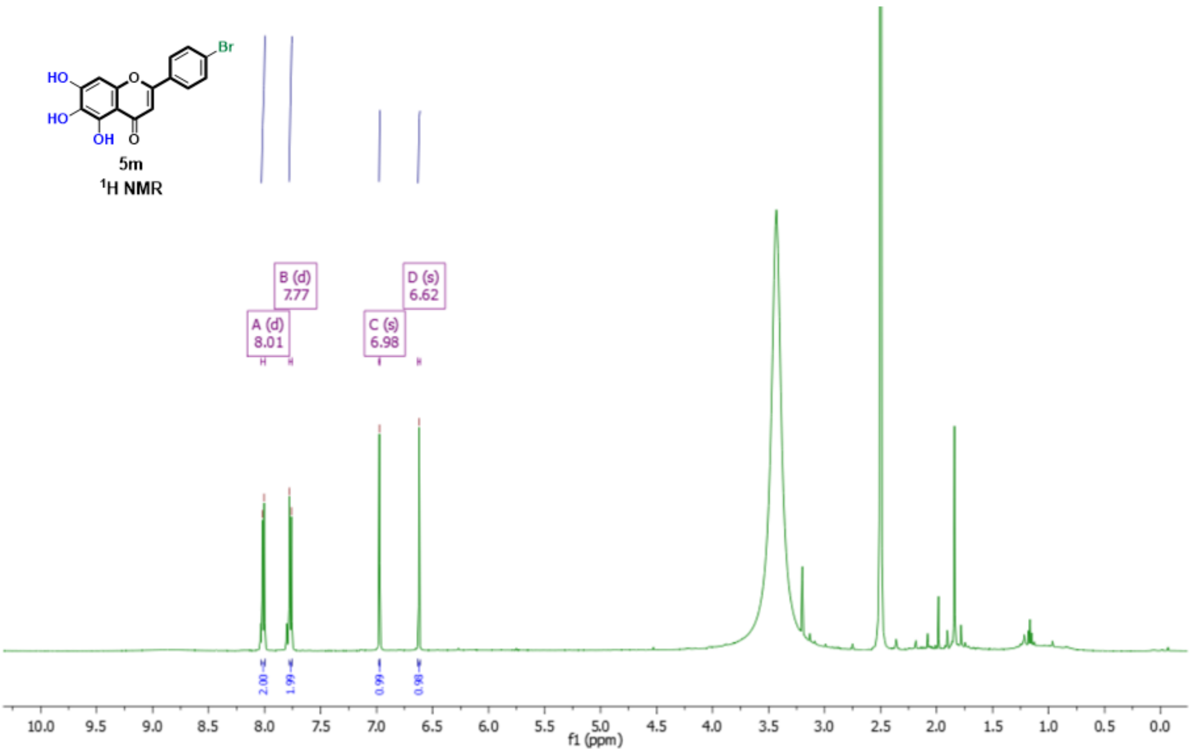

Figure S44 ^{1}H NMR of **5m**

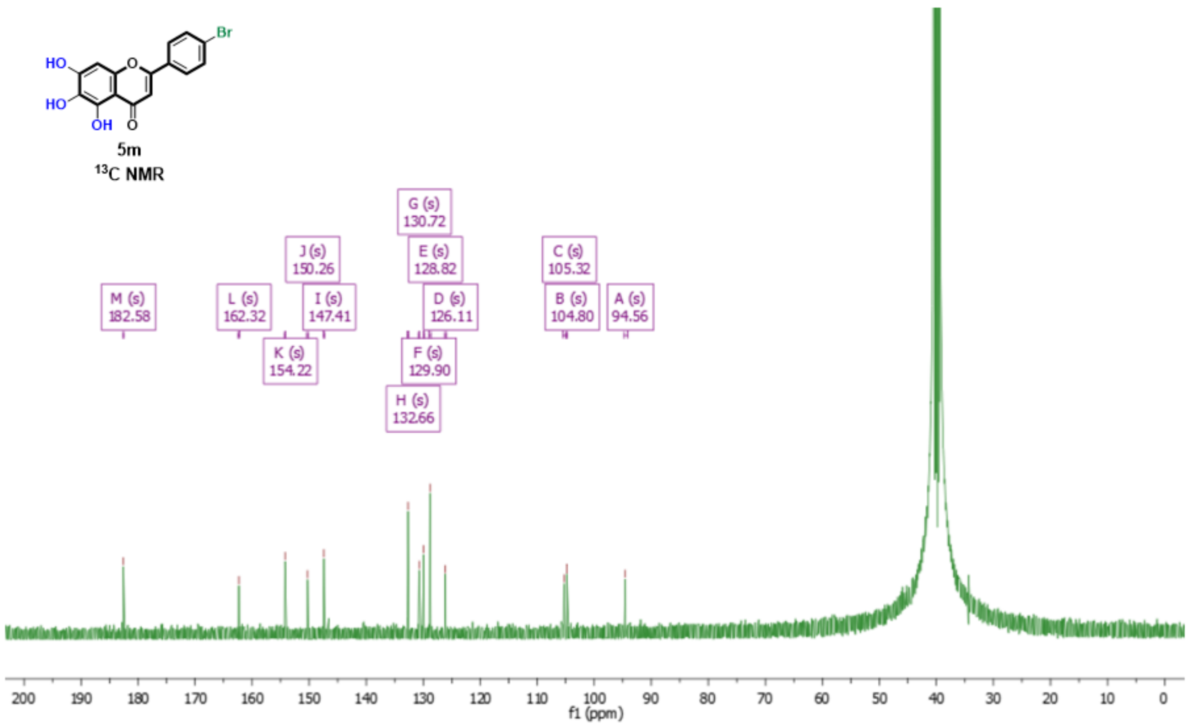

Figure S45 ^{13}C NMR of **5m**

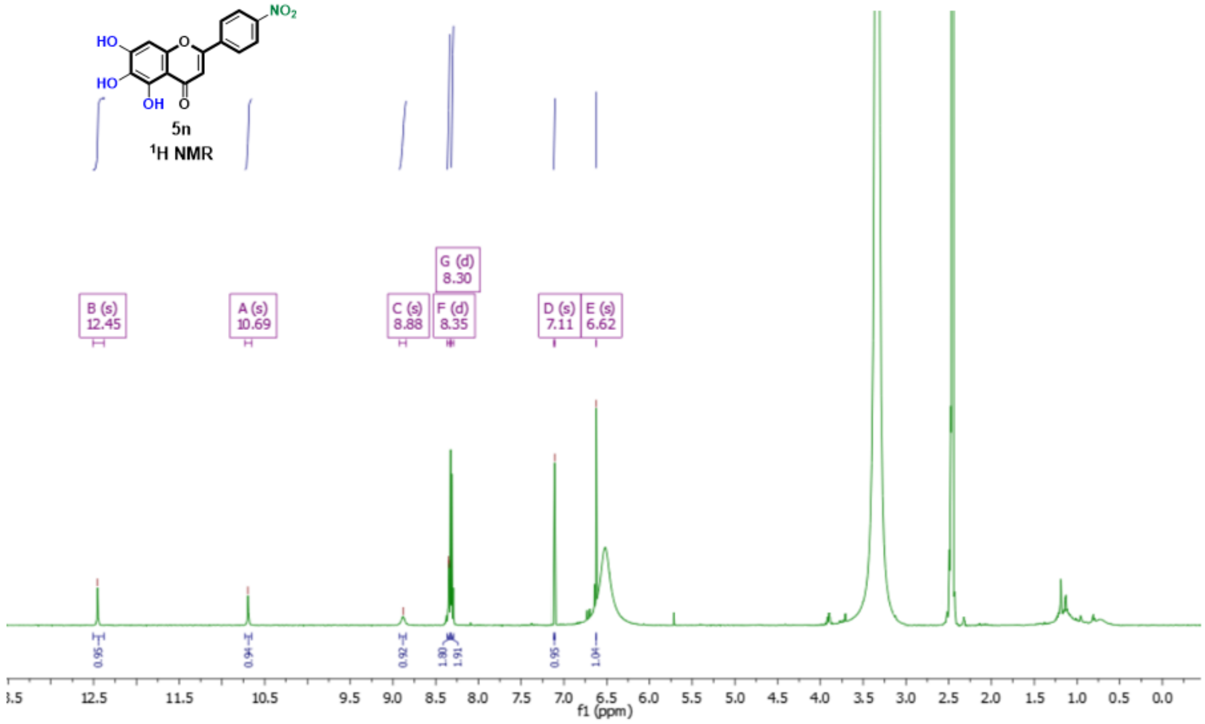

Figure S46 ^{1}H NMR of **5n**

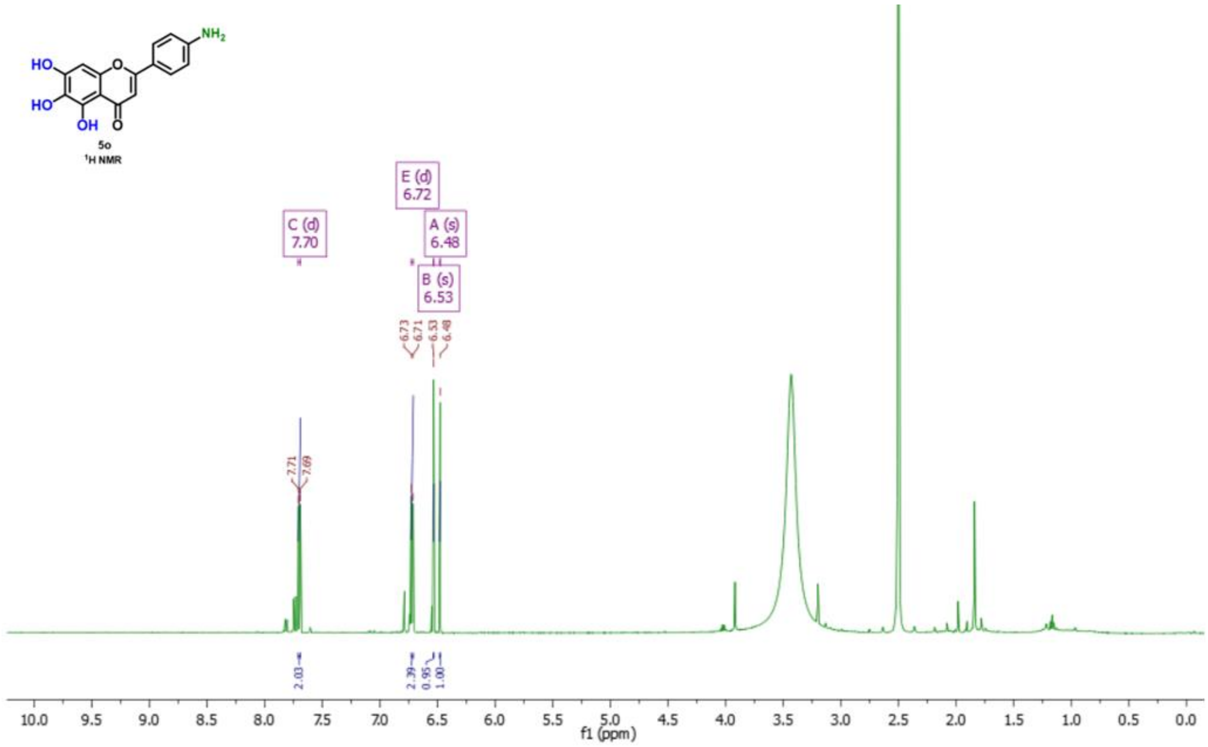

Figure S47 ^{1}H NMR of **5o**

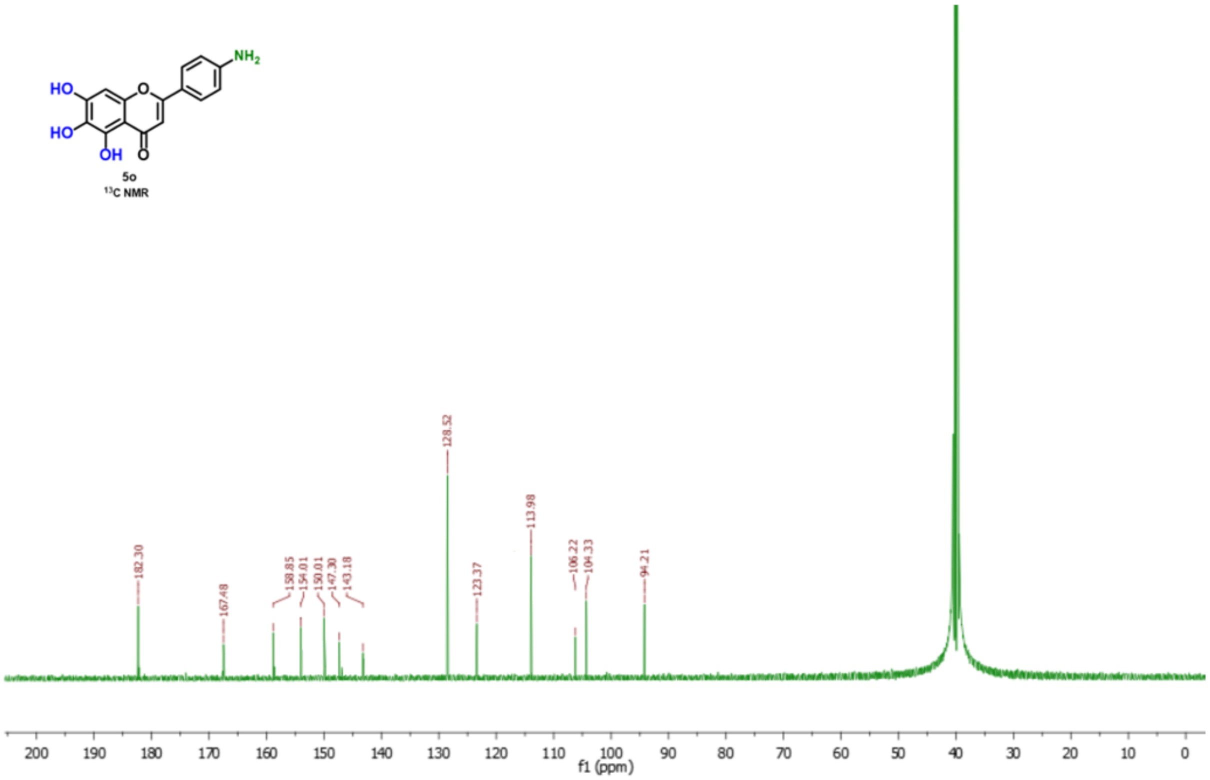

Figure S48 ^{13}C NMR of **5o**

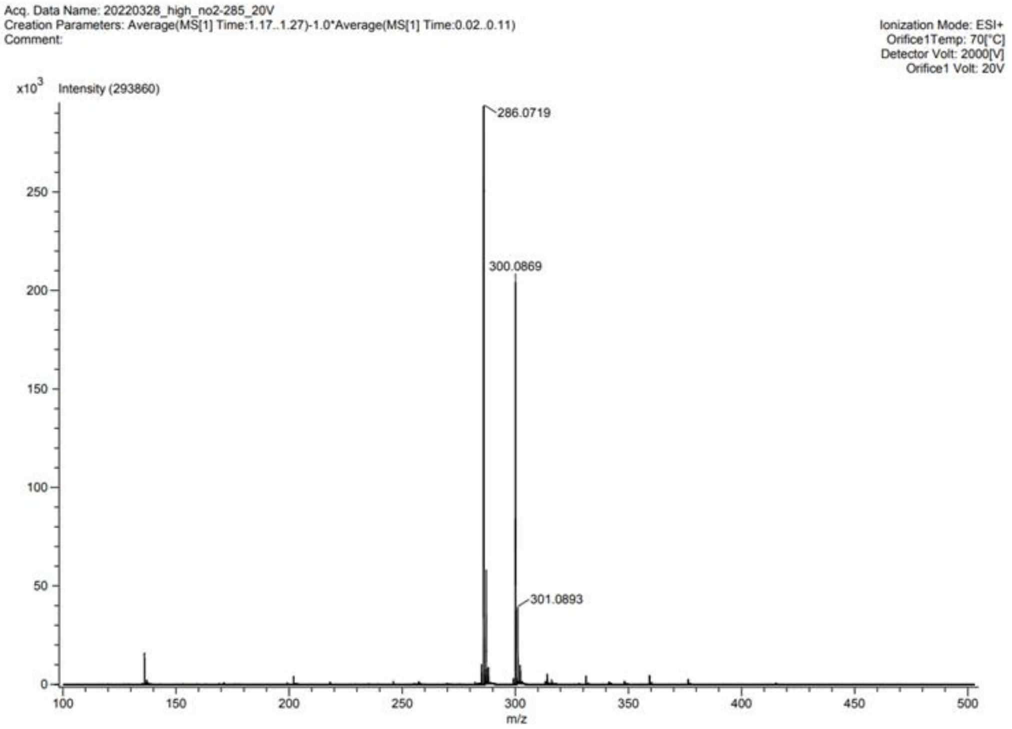

Figure S49 HSMS of **5o**

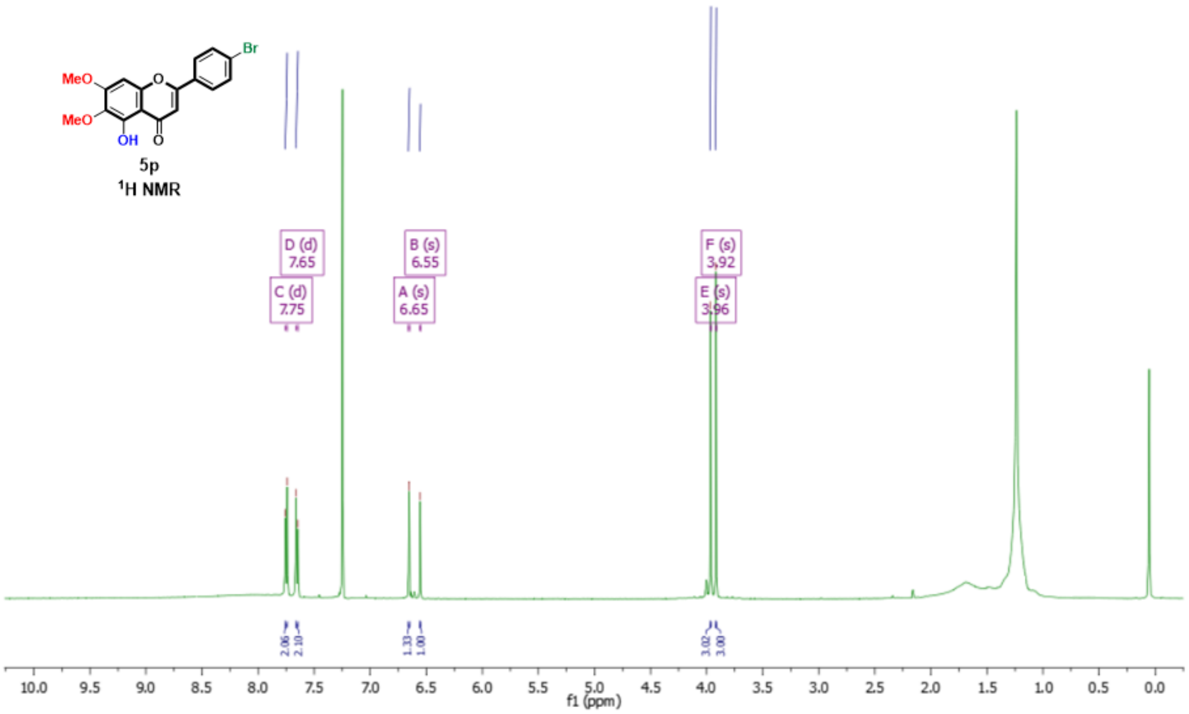

Figure S50 ^{1}H NMR of **5p**

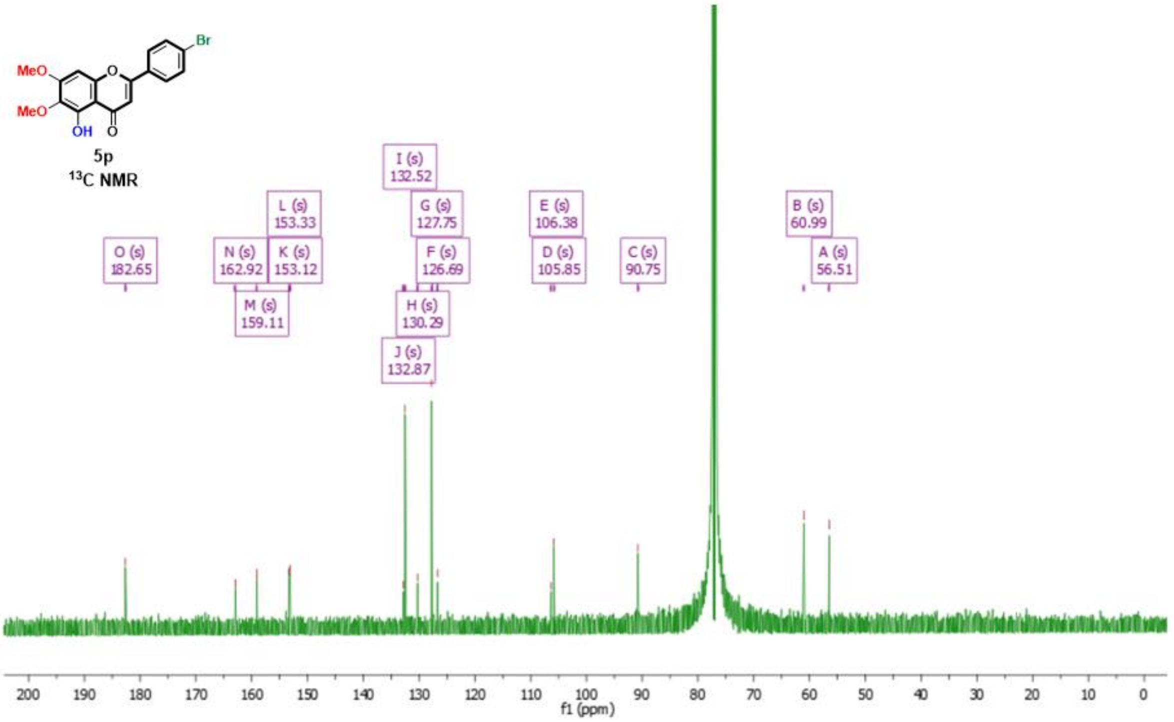

Figure S51 ^{13}C NMR of **5p**

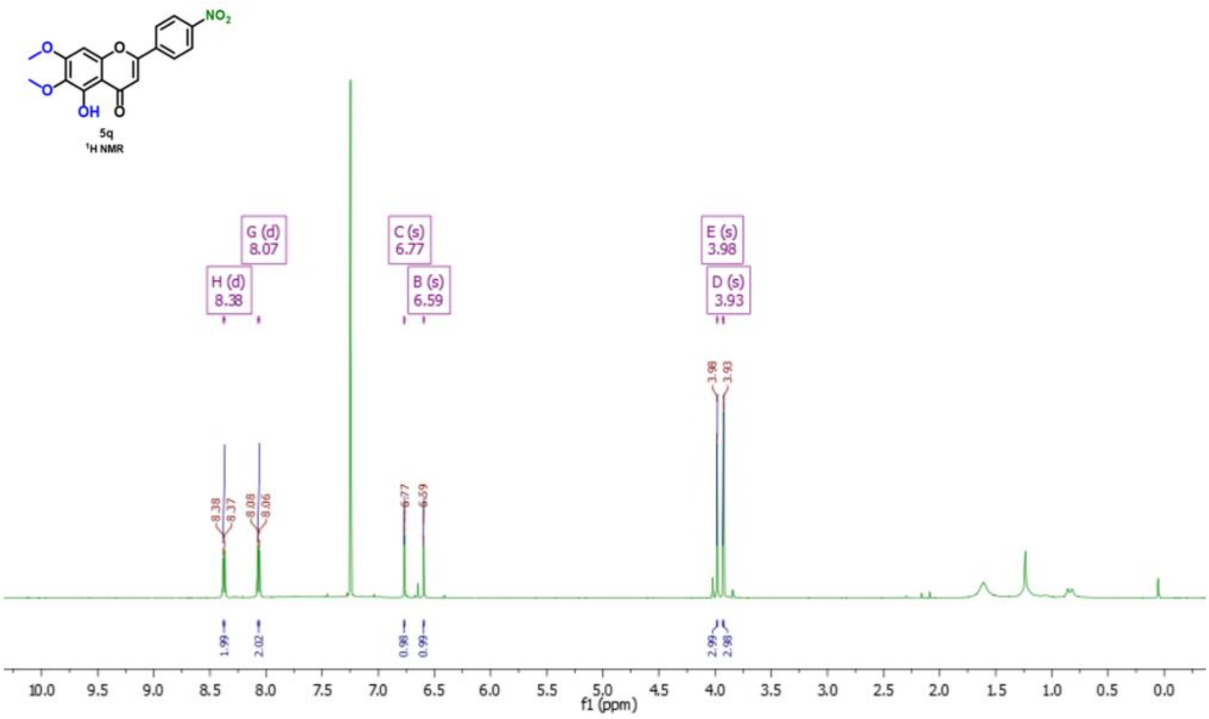

Figure S52 ^{1}H NMR of **5q**

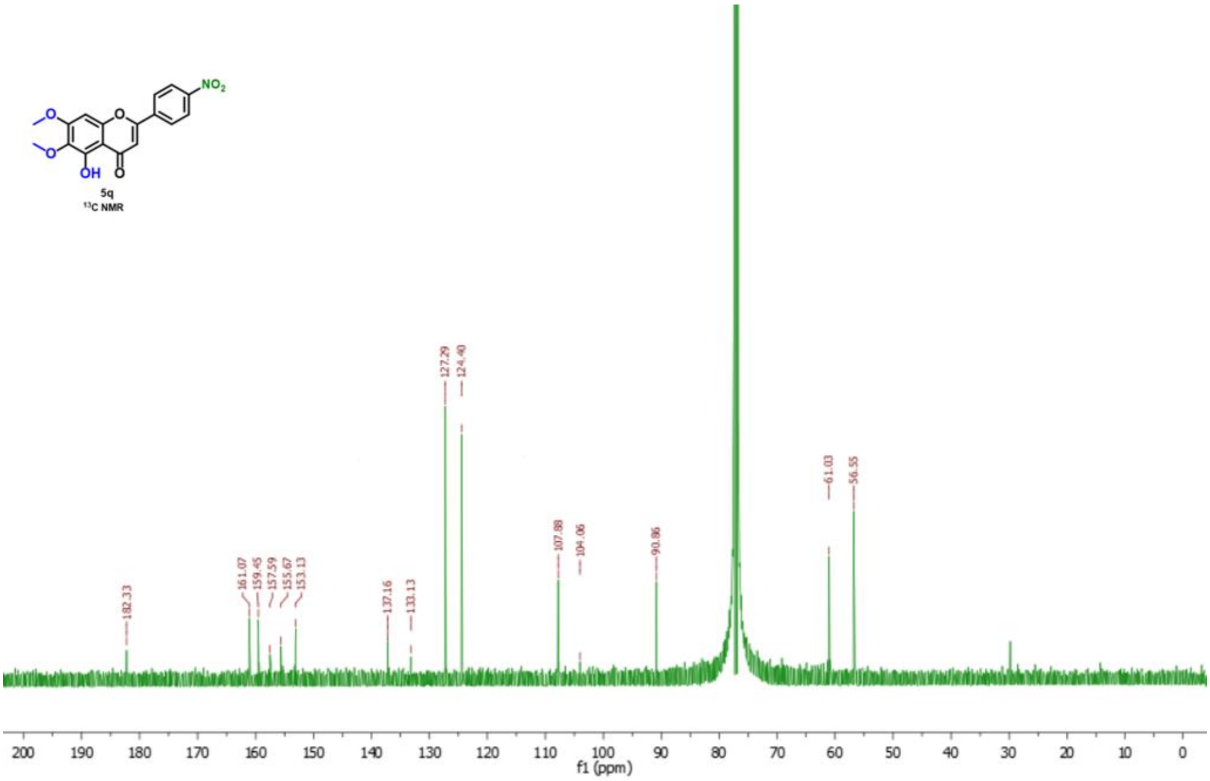

Figure S53 ^{13}C NMR of **5q**

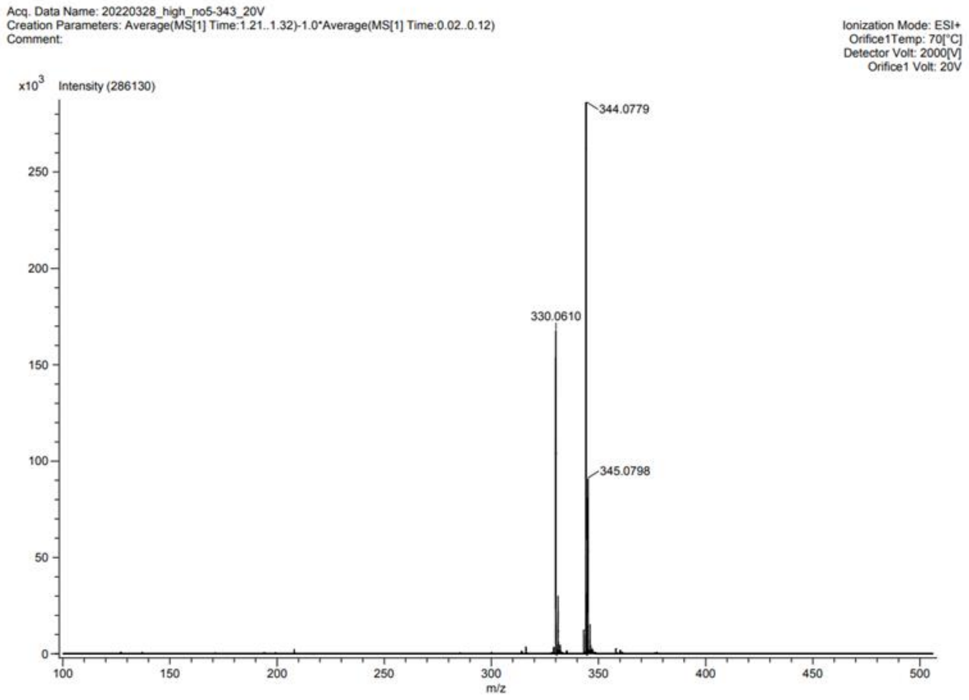

Figure S54 HSMS of **5q**

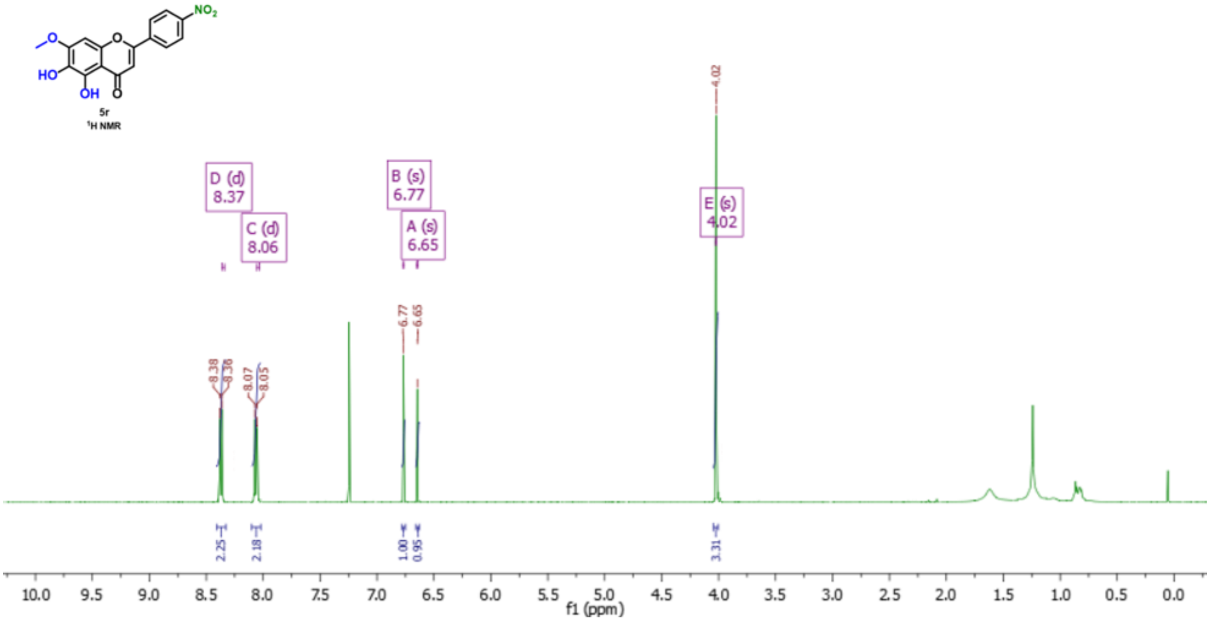

Figure S55 ^{1}H NMR of **5r**

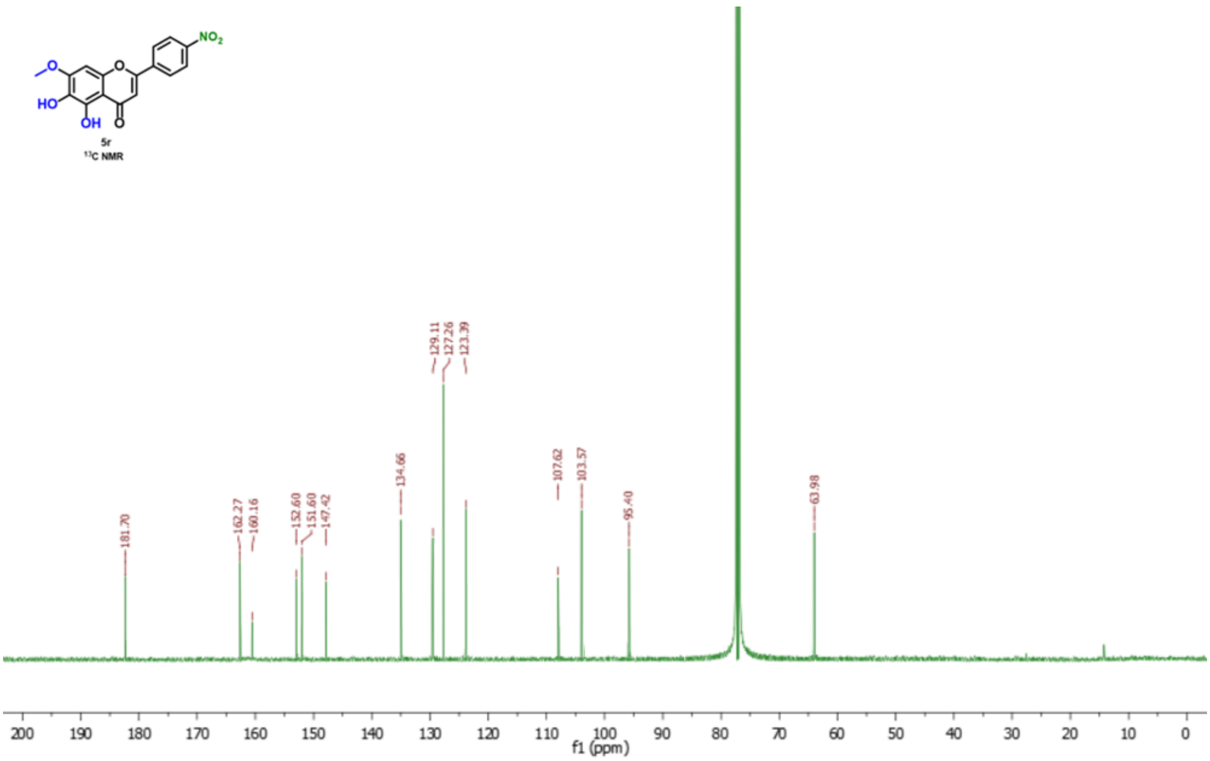

Figure S56 ^{13}C NMR of **5r**

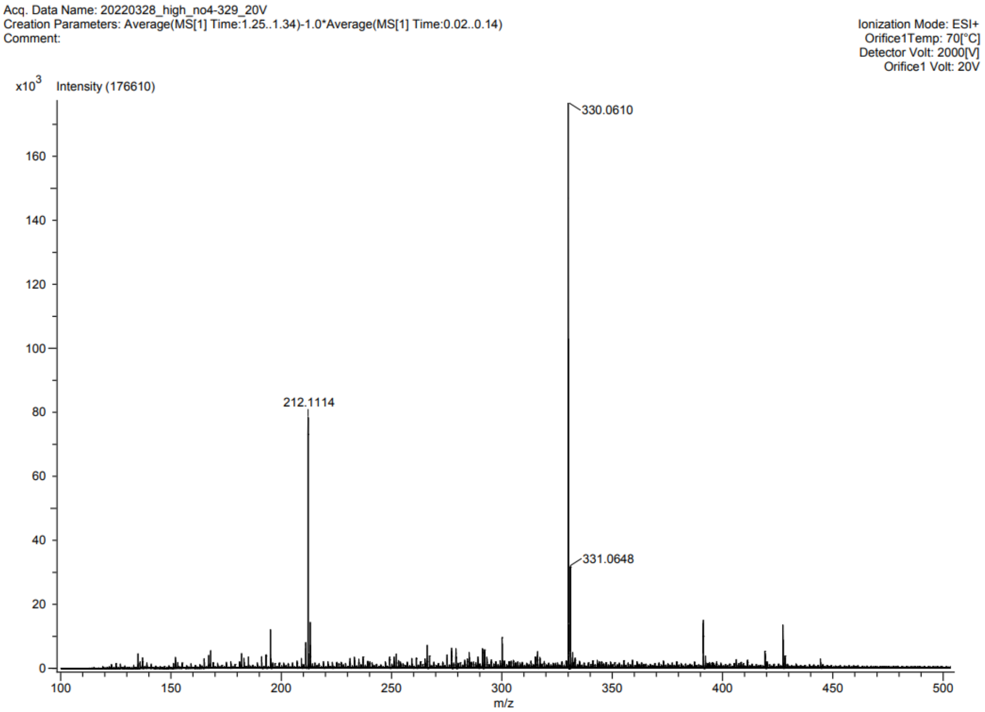

Figure S57 HSMS of **5r**
